# Farming System and Nematodes Affect the Rhizosphere Microbiome of Tropical Banana Plants

**DOI:** 10.1111/1758-2229.70155

**Published:** 2025-07-09

**Authors:** Mariantonietta Colagiero, Luis Pocasangre, Aurelio Ciancio, Isabella Pentimone, Laura Cristina Rosso

**Affiliations:** ^1^ Consiglio Nazionale Delle Ricerche, Istituto per la Protezione Sostenibile Delle Piante Bari Italy; ^2^ Escuela Agrícola Regional del Tropico Húmedo (EARTH), Limón, and Centro Agronómico Tropical de Investigación y Enseñanza—CATIE Turrialba Costa Rica

**Keywords:** 16S rRNA gene, ITS1‐2, metabarcoding, *Musa*, *Radopholus*, *Trichoderma*, *Xiphinema*

## Abstract

We studied the effects of farming systems and soil nematodes on the rhizosphere microbial profiles in three banana farming systems (conventional, barbecho and organic) compared with non‐cultivated controls. Bacterial 16S Amplicon Sequence Variants (ASV) and fungal ITS1‐2 OTUs were obtained by NGS from experimental fields in Costa Rica, each with a given farming system. Plant‐parasitic nematodes included *Meloidogyne*, *Helicotylenchus*, *Radopholus*, and other species. The banana cultivation and, to a minor extent, the field management type influenced the rhizosphere ASV and OTUs abundances, with a higher diversity found in organic versus conventional crops, with the organic control as the most biodiverse. Diversity indices showed differences for the total number of individuals (lowest in conventional banana) and rare species (highest in organic controls). Fungi differed for the highest species richness in the organic controls. Soil variables affecting microbial abundance included low Fe content and acidic pH. Nematodes were associated with microbial taxa that were specific to each herbivore species or feeding group, with omnivores/predators influencing microbial profiles mostly in the organic crop and controls. The organic management had the lowest impact on the diversity of belowground nematodes and rhizosphere microbiome, highlighting its beneficial potential in sustainable banana production and agroecosystem resilience.

## Graphical abstract

The banana crops showed a core microbiome of 77 ASV, mostly belonging to Proteobacteria, Actinobacteria, and Bacilli. Specific groups of bacteria and fungi were linked to each nematode species or feeding group, with a core microbiome of correlated taxa found only when considering nematodes by feeding groups.

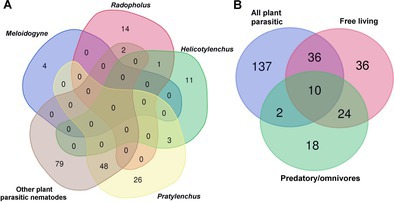

## 1 Introduction

Soil microbial diversity and ecosystem services are crucial for plants productivity and response to biotic and abiotic stress factors, varying in function of variables such as crop age and management, plant residues, soil properties, climate, and surrounding environment (Reiss and Drinkwater 2022; Beaumelle et al. 2023; Williams et al. 2023). Cultivation technology and intensification level also affect the profile composition and richness of the soil microbial communities and, indirectly, their services (Liebig et al. 2005; Hartman et al. 2018; Banerjee et al. 2019; Reiss and Drinkwater 2022).

The balance and impact of soil services largely depend on their stability and similarity. Key services for crop production include nutrient cycling, N_2_ fixation, mobilisation of soil mineral content, organic matter decomposition, as well as pest and disease regulation (Reiss and Drinkwater 2022; Goemann et al. 2021). In particular, balanced soil ecosystems, rich in beneficial antagonists, can regulate indigenous pests and pathogens. However, intensive soil exploitation and crop intensification may alter such balance, requiring further interventions to counteract the lack of a natural regulation (Reiss and Drinkwater 2022; Riascos‐Ortiz et al. 2022). Knowledge for sustainable plant protection and environment‐friendly production is hence required, in particular in intensive farming systems.

In tropical agriculture, intensive crops such as conventional export banana (*Musa* spp.) are heavily dependent on synthetic inputs to maintain actual yield levels (Brühl et al. 2023). Few banana genotypes support food production for a million people in the Tropics and provide a significant income for export‐oriented farms. Banana crops, however, are often characterized by a substantial environmental impact, causing sustainability problems including social, economic and food security issues worldwide. These constraints add to severe plant‐protection vulnerabilities due to many pests and diseases and to the narrow genetic basis of the banana crops (Valenciano et al. 2015; Bebber 2023). Moreover, plant protection practices are known to affect biodiversity, in particular in tropical agroecosystems, as long‐term applications or prolonged exposure to pesticides and fungicides negatively impact both above‐ and belowground species diversity (Goulson 2013; Brühl and Zaller 2019; Vargas 2006). Although the effects on biodiversity of water organisms have been ascertained (Salazar‐Zúñiga et al. 2019), few data are available on the impact of chemicals on the soil microbial communities, including plant endosymbionts (Brühl et al. 2023; Echeverría‐Sáenz et al. 2018; Castillo et al. 2000; Gomez‐Lama Cabanas et al. 2021).

The cultivation of banana in the Caribbean regions is mostly based on Cavendish clone (*Musa* AAA) monocultures. Common diseases include Black Sigatoka leafspot caused by *Mycosphaerella fijiensis*, controlled through repeated fungicide applications. Pests include the burrowing nematode *Radopholus similis* , managed with 2–4 annual applications of carbamate and/or organophosphate nematicides (Vargas 2006). Cavendish banana clones, resistant to Fusarium wilt, are highly susceptible to *R. similis* attacks that cause root lesions and plant toppling. This pest spread worldwide when the Cavendish clones replaced the Gros Michel variety, susceptible to the Fusarium wilt.

In Costa Rica, approximately 75 kg ha^−1^ of pesticides are applied annually to over 40,000 ha of banana monoculture (Echeverría‐Sáenz et al. 2018). Many fungicides and nematicides, mostly banned for health and environmental issues elsewhere, are still applied in plantations, with high doses and frequencies. Nematicides such as carbofuran, cadusaphos, or ethoprophos, applied in plantations, concentrate in waters and sediments threatening wildlife and environmental quality (Castillo et al. 2000). There is hence a need to implement alternative farming systems with a lower environmental impact, that is, organic or other management systems based on removal of crop residues or fallow.

Nematode communities represent a fundamental component of the banana soil and rhizosphere food webs, whose structure is influenced by the aboveground plant richness affecting herbivores and pests such as *Helicotylenchus multicinctus* (Poeydebat et al. 2017). However, not all nematodes produce a negative impact on plants. In the rhizosphere food webs, the nematode guilds usually include bacterivores, fungivores, predators, and omnivores. Free‐living bacterivore species provide a broad range of beneficial services, including recycling of nutrients, C storage, organic matter decomposition, microbial dispersal, and regulation of other soil organisms. Moreover, all nematodes interact, in their food webs, with a specific, associated, and biodiverse microbiota through processes including endosymbiosis, parasitism, feeding, and/or predation (Costa et al. 2011; de Vries and Wallenstein 2017; Elhady et al. 2018; Topalović et al. 2020; Malacrinó et al. 2021).

Studies comparing organic and conventional banana crops revealed higher numbers of macroinvertebrates and a greater biodiversity in the soil from organic plantations (Castillo et al. 2000). However, comparisons between plantations with high and low pesticide levels did not appear consistent for the microbial community structures (Vargas 2006). The situation is furthermore complicated by the limited knowledge available thus far on the biology of bacteria still unclassified (usually > 90% of total taxa), often difficult to characterise as concerns the services in which they are involved (Delgado‐Baquerizo 2019; Gschwend et al. 2021). Farming systems and practices such as the application of nematicides, the addition of organic matter and/or the presence/removal of root residues also affect nematodes guilds beneficial to plants, whereas cover crops increase their diversity (Djigal et al. 2012). Bacteria and fungi also show different dependencies on farming practices such as tillage and other management systems, respectively (Hartman et al. 2018).

Understanding the links of the soil–plant–microbiota system components with other agroecological factors is pivotal to improve banana crops resilience and minimise negative impacts. Costa Rica is a world‐renowned hotspot of biodiversity, in particular for aboveground organisms. Belowground diversity is less explored and few data are available on fundamental issues related to that is, soil microbial diversity and its conservation in banana agricultural soils. In Costa Rica, farms, often interspersed with natural rainforest ecosystems, nematicides are the most biodiversity‐impacting compounds as pests such as root‐knot (*Meloidogyne* spp.), burrowing ( *Radopholus similis* ), spiral ( *H. multicinctus* ) and lesion (*Pratylenchus* spp.) nematodes are widespread, affecting productions with significant economic and environmental costs (Vargas 2006; Roderick et al. 2012; Sikora et al. 2018; Coyne 2021).

The study of complex systems such as the plant and rhizosphere environments may benefit from advanced informative tools such as metabarcoding (Sudermann et al. 2024). There are several examples of the potential of this approach in the literature on this crop. Metabarcoding highlighted the effect of the cover crop on the natural regulation of the banana weevil *Cosmopolites sordidus* in two farming systems (Mollot et al. 2014). In Australia, metabarcoding data showed dependence of the bacterial and fungal taxa on the banana soil, genotype, and plant compartments sampled, consistently identifying common core taxa across different farm/samples and regions (Birt et al. 2022, 2023). Metabarcoding data from sub‐tropical banana farms showed a direct effect of cultivation and latitude on the rhizosphere microbiome profiles, with specific microbial subsets associated with each nematode guild (Ciancio et al. 2022). Metabarcoding was also applied to study the variations in the microbial diversity of banana plants, in relation to the compartments sampled (Gomez‐Lama Cabanas et al. 2021; Birt et al. 2022).

Aim of this study was to evaluate the effect of the banana management practices and soil nematode guilds on the rhizosphere microbiota. We sampled three fields, each following a different farming system, located in the experimental station of the EARTH University. For each farming system we also sampled grasses from adjacent control sites, free of banana roots. Our goal was to: (i) determine how (and if) the banana cultivation systems affect the rhizosphere bacterial and fungal profiles, using metabarcoding of the 16S ribosomal rRNA and ITS1‐2 genes sequence data, and (ii) measure the eventual relationships linking the soil properties and microbial components with the levels of plant‐parasitic and other nematode groups, eventually present.

## 2 Materials and Methods

The metabarcoding study was carried out in three plantations with similar climatic conditions and soil, characterized by a 3 years old cultivation of Cavendish banana *M. acuminata* (AAA group) ‘Grand Nain’, and differing by the farm management system applied. The fields (1.0 ha each, all planted with 1650 plants/ha) were located within the experimental station of the EARTH University (Las Mercedes, Guácimo, Limón, Costa Rica, http://www.earth.ac.cr), in a fragmented tropical forest environment, with geographic coordinates: 10° 13′ 08″ N and 83° 36′ 30″ W (‘barbecho’ and conventional fields, 34 m elevation above sea level), and 10° 13′ 26″ N and 83° 35′ 15″ W (organic field, located at 2.35 km from the other fields, 31 m elevation above sea level). The farms were characterized by similar climatic conditions due to their proximity, with a tropical climate typical of the Atlantic Caribbean zone, and 28°C as annual mean temperature for the area (range 22°C–34°C), an average 84% HR (range 80%–90%), and an annual rainfall > 3500 mm (mean = 3609 mm). The banana plantations were renoved using the same planting material. The practices investigated were as follows: (i) a “conventional” cultivation management (herein coded as CV), based on traditional practices applied in the region, with 8–9 months of growing cycle with two applications of nematicides (oxamyl alternated to fluopyram), fungicides (one treatment with fungicides per week for controlling Black sigatoka, application performed by airplane with low altitude flights) and six annual applications of synthetic fertilisers applied in soil (total *N* = 400 kg/ha, *p* = 60 kg/ha, K = 500 kg/ha), and an application per year of CaCO_3_ (1 ton/ha); (ii) the “barbecho” system (herein coded BRB), characterised by a conventional management plus the adoption of a fallow period before the banana plantation replanting, in which the banana plants and residues are completely removed and the banana crop is re‐planted after a non‐cultivation period of 6 years; (iii) an “organic” crop (herein coded OR), including 9–11 months of growing cycle with plant management carried out without any application of pesticides or chemical fertilisers, relying on organic fertilisation, and no fallowing. The organic field was fertilised with an organic liquid fertiliser obtained from the leaching of harvested banana bunch raquis. Every 2 weeks, 1 L of this leaching fluid was applied per plant (pH = 7.75, EC = 13.3 dSm/m, *p* = 31, *K* = 6194, Ca = 269, Mg = 77, Fe = 5.7, C = 2.1, Zn = 0.2, Mn = 0.7, NO = 28 ppm, means of three replicates). Additionally, 3 kg of a compost based on the banana rachis (solid residues from the leaching process) were also applied per plant, at the same time schedule.

### 2.1 Sampling, Nematode and Soil Analyses

A total of 30 samples were collected in August 2019, under the canopy of adult banana plants or from grass (controls). Five replicated banana plants from five replicated plots within each farming system field were sampled at the post‐harvest stage, collecting 1 L of soil around roots. For each farming system, five additional samples were also collected from around the roots of grass growing at a distance of 10–15 m from the sampled banana plants and used as comparative adjacent controls (without banana roots). The grasses were mostly Poales (i.e., *Rotboellia colchicinensis* and *Cyperus rotundos*). For sampling, the soil was collected at a depth of 15–20 cm after exposing the roots of banana or grasses with a hoe, collecting soil and 10–20 cm long root fragments in a clean plastic bag. The samples were then stored at 4°C and processed within 36 h for nematode extraction using Cobb's sieving and decanting technique. For metabarcoding, an aliquot of soil was then stored at −80°C until RNA extraction. The nematodes collected on the finer sieve were then concentrated in a final 100–150 mL water volume for subsequent counting in a 2 mL counting chamber with a light microscope at 40×, in three replicated countings per sample.

The nematodes were classified by their feeding behaviour as free‐living (bacterial and fungal feeders), omnivorous/predatory (mononchids and other dorylaims), or plant‐parasitic (Tylenchida, a Trichodorid and herbivorus Dorylaimida). For identification at the species or genus level of the plant‐parasitic nematodes, the specimens were placed on a slide in a temporary water mount and then examined with a light microscope at higher magnifications (100–250×). For this purpose, the nematodes were hand‐picked from the suspension and then examined on a temporary slide. The nematode densities (in 100 mL soil) were classified in the mapping file for the bioinformatic analyses as medium (M, within a 10% confidence interval around all samples mean), and lower (L) or higher (H) when falling below or above the 10% mean confidence interval (Table S1A) (Djigal et al. 2012; Ciancio et al. 2022). For each sample, a 10 mL aliquot of rhizosphere soil was stored at 4°C for a 2 days and then at −80°C for subsequent RNA extraction and metabarcoding sequencing.

Chemical soil analyses (performed for each sample by the Soil, Leaf and Water Laboratory of EARTH University) showed similar conditions as concerning soil type (volcanic), with a sandy‐clay texture and a moderately acidic to neutral pH, with a range 5.0–7.2 (6.5–7.2 for ORG, 5.0–6.0 for BRB and 5.4–6.0 for CV samples). Soil pH was classified as acidic (AC, 5.1–5.5), moderately acid (MAC, 5.6–6.0), slightly acid (SAC, 6.1–6.5), or neutral (N, 6.6–7.3). For metabarcoding analyses, the soil content of P and other elements (K, Ca, Mg, Na, Fe, Cu, Zn, Mn, B, S) was coded as low (L), medium (M) or high (H) when their values were < 80% of their means, within a 20% interval around the means or higher, respectively (Table S1B).

### 2.2 Metabarcoding Data Production

The collected soil samples were subjected to metabarcoding analysis to determine the effect of the farming systems on the bacterial and fungal soil profiles. Total RNA was extracted from 2 g of rhizosphere soil collected from banana roots or from adjacent control roots for each sample (30 extractions) using the RNeasy PowerSoil Total RNA kit (Qiagen, UK—MoBio Laboratories Inc.), following the manufacturer instructions. The concentration of the isolated RNA was determined by measuring the absorbance at 260 nm with a Nanodrop spectrometer. Subsequently, the extracted material was converted to cDNA using the SuperScript IV (Invitrogen, USA), following the manufacturer protocol and according to the Illumina sequencing protocol. The integrity of the synthetized cDNAs was confirmed by electrophoresis in a 1.5% agarose gel. The cDNA was then purified using a Qiaquick PCR Purification kit (Qiagen, UK), and then stored at –80°C until sequencing. The cDNA was used for PCR amplification of the bacterial 16S rRNA gene and the fungal internal transcribed spacer (ITS1) regions.

Amplification and sequencing of the hypervariable region V3–V4 (Van de Peer et al. 1996; Baker et al. 2003; Clarridge 2004; Takahashi et al. 2014) of the 16S rRNA gene were performed to characterize the bacterial composition of the samples. 16S rRNA gene sequencing allows detection of known or unclassified species within environmental samples, present in the reference database (Yang et al. 2002; Liu et al. 2007, 2008; Caporaso et al. 2010). The 16S rRNA gene primers 341F (5′‐CCTACGGGNGGCWGCAG‐3′) and 805R (5′‐GACTACHVGGGTATCTAATCC‐3′) were selected for affinity, flanking conserved motifs, and were used to amplify the amplicons for bacteria identification (Herlemann et al. 2011; Klindworth et al. 2013). For the ITS1 region, primers ITS1 (5’‐TCCGTAGGTGAACCTGCGG‐3’) and ITS4 (5’‐TCCTCCGCTTATTGATATGC‐3’) were used (White et al. 1990).

The library preparation and sequencing were carried out through a commercial service (IGA‐Technology Services, Udine, Italy, www.igatechnology.com), using a MiSeq sequencing system (Illumina), run in paired‐end with a 300‐bp read length.

### 2.3 Bioinformatic Workflow

The 16S rRNA gene sequence reads were processed with QIIME2 (Quantitative Insights Into Microbial Ecology, ver. 2, pipeline qiime2‐2021.11) (Bolyen et al. 2019). Low‐quality sequences were removed after importing the demultiplexed sequence data. The DADA2 method for Amplicon Sequence Variants (ASV) inference was used to process the 16S rRNA gene amplicon data, producing a feature table containing high‐resolution ASV (Callahan, McMurdie, et al. 2016; Callahan, Sankaran, et al. 2016). The taxonomy was assigned in the next step, based on the exact correspondence between ASV and reference strains present in the database used, in this case the Greengenes dataset (file gg‐13‐8‐99‐515‐806‐nb‐classifier.qza, ver. 2013_8) (DeSantis et al. 2006; McDonald et al. 2012).

Due to the reduced coverage of ASV databases for fungi, PandaSeq (https://github.com/neufeld/pandaseq) and QIIME1 were used to produce the ITS1‐2 Operational taxonomic units (OTUs) table (Caporaso et al. 2010; Masella et al. 2012). PandaSeq was used to assemble the single read contigs, merging the forward and reverse reads of the raw sequences, with the following parameters: sequences with unidentified nucleotides = filtered; length of overlapping region = 5–50 nt (min‐max); lengths of contigs (min‐max) = 540–600 nt (Feibelman et al. 1994; Claesson et al. 2010). For each sample (except a single BRB sample), the single fasta format file of high‐quality assembled sequences was obtained by merging data and then used as input for processing with QIIME 1.9 (Caporaso et al. 2010). The OTUs assigned through the implementation of UCLUST (https://doi.org/10.15156/BIO/2938079) (Kõljalg et al. 2013; Edgar 2010; Nilsson et al. 2019), applying a 97% identity threshold to discriminate at the species level (Caporaso et al. 2010). An OTUs.biom file was then constructed using as a reference file the UNITE data set for fungi (ver. 9.0).

For both ASV and OTU tables, an equal variance two‐sided *t*‐test (*p* ≤ 0.05) with a 95% confidence interval and effect size filters (difference between proportions = 0.8 or ratio of proportions = 2) was applied when comparing data of sample groups with STAMP (Statistical Analysis of Metagenomic Profiles, ver. 2.1.3) (Parks and Beiko 2010; Parks et al. 2014) (http://kiwi.cs.dal.ca/Software/STAMP). Comparisons included samples grouped by the following variables, included in the data mapping file: crop (banana or control), farm management system (conventional, barbecho or organic), sample management system (conventional, barbecho or organic banana, and corresponding controls), density levels of *Meloidogyne*, *Radopholus*, *Helicotylenchus*, and of other plant‐parasitic, free‐living or omnivorous/predatory nematodes, pH, P and other soil element contents. The entire samples were used as parent level, applying a two‐tailed Student's *t* test at different taxonomic profile levels, with other comparative statistics. To keep unclassified taxa in the analyses, their higher levels were identified in the hierarchy (and eventually represented in STAMP plots) by using the ASV or OTU available codes as tags for the genus or species unclassified levels, and using the lower available classification for subsequent levels. Heatmap or bar plots of only significantly different ASV or OTUs (ANOVA, with 0.95 post hoc Tukey–Kramer test, filtering threshold: *p* ≤ 0.05) were shown with trees produced with average neighbour UPGMA and a 0.65 dendrogram clustering threshold. Principal Component Analysis (PCA) for ASV or OTUs samples was also performed, with STAMP default parameters.

The ASV and OTU tables were used for further analyses with R ver. 4.1.2 (R Core Team 2013), running in R Studio (ver. 1.4.1106). The library *mctoolsr* was used to produce the samples Bray–Curtis dissimilarity matrices and MDS plots, the taxa summary tables for the whole dataset or for selected taxa or sample groups, including permutational multivariate analysis of variance (PERMANOVA) (Leff and Fierer 2013; Leff 2016). Further R libraries used included *ggplot2* (Wickham 2016) and *psych* (Revelle 2017) for Spearman's correlations, calculated after filtering the ASV at a sequence sum for all samples ≤ 150 and the OTUs at a mean of sequences per sample < 5. The OTUs and ASV dataset were also filtered for presence in at least five samples, to eliminate poorly represented taxa (Barberán et al. 2012). Pearson's correlations and diversity indices were calculated using PAST, with Tukey's pairwise comparisons (Hammer et al. 2001). Venn diagrams were produced using an online service (http://bioinformatics.psb.ugent.be/webtools/Venn) (Heberle et al. 2015).

## 3 Results

### 3.1 Microbials Links With Nematode Guilds

The nematodes found in the banana rhizosphere soil included second stage juveniles (J2) of *Meloidogyne* sp., as well as adult and juvenile stages of *H. multicinctus* , *R. similis* , and *Pratylenchus* sp. Other taxa present in the fields included two *Xiphinema* spp. ( *X. brasiliense* and *X. longicaudatum*) and an unidentified criconematid. Densities of *Meloidogyne* sp. were highest in the organic banana plants, whereas *R. similis* and *H. multicinctus* were highest in conventional banana crops, with *H. multicinctus* showing more uniform levels. *Xiphinema* spp. were found only in the controls of the organic and barbecho crops that also showed the highest numbers of criconematids. Free‐living and omnivorous/predatory nematodes peaked in the organic controls. The omnivorous/predatory nematodes showed a high density also in the rhizosphere of organic banana plants (Table S1A).

Correlations were performed on a dataset of 510 taxa (129 bacteria and 381 fungi) and 18 soil variables, for 29 samples (sample BRB4 was eliminated due to a low number of fungal taxa). Pearson's correlations showed that the numbers of *Meloidogyne* sp. and of predatory/omnivore nematodes were correlated to some soil chemical variables. The *Meloidogyne* J2 were significantly linked to pH and to the K, Mg, P, and Cu content of soil, likely due to higher soil fertility and root abundance. *Radopholus similis* showed a positive correlation with *Helicotylenchus* and the soil boron content, whereas *Pratylenchus* sp. was correlated with other plant‐parasitic species (*Xiphinema* spp., criconematids) and the soil Na content. *Xiphinema* spp. and criconematids were positively correlated with the soil extractable acidity and inversely related to its boron content. The free‐living nematodes were linked to the predatory/omnivores and to P, Fe, and Mn soil content. Finally, the predatory/omnivores also showed correlations with soil pH and with most elements, excluding Na and boron, and an inverse relationship with S (Table S1A,C).

No significant difference was found among the maturity indexes (MI) of the six sampled fields (one‐way ANOVA, data not shown). However, the banana samples of each farming system showed index values higher than those of their corresponding controls (Table S1A).

Pairwise and Bonferroni‐adjusted Spearman's correlations (*p* < 0.05) showed specific sets of fungi and bacteria which appeared linked to a given nematode species or feeding group. *Meloidogyne* sp. had the lowest number of correlated taxa, including bacteria such as *Azospira restricta* , *Parasegitibacter luojiensis*, and *Corynebacterium pilosum* . *Radopholus similis* showed a higher number of correlated taxa (22), including *Wickerhamiella* sp., three *Aspergillus* species, and unclassified members of Rozellomycota, Agaricomycetes, and SH0249586. *Helicotylenchus multicinctus* showed 22 correlations mostly with fungi (i.e., *Aureobasidium pullulans*, *Curvularia coicis*, *Tetracladium furcatum*, *Trichoderma asperellum*, *Cladosporium herbarum* and other unclassified taxa), and only one bacterium, *Priestia (Bacillus) megaterium*. Higher numbers of significant correlations were found for *Pratylenchus* sp., *Xiphinema* spp., and criconematids, predatory/omnivores, and free‐living (Table S1C), indicative of multiple services deployed in these interactions.

**FIGURE 1:**
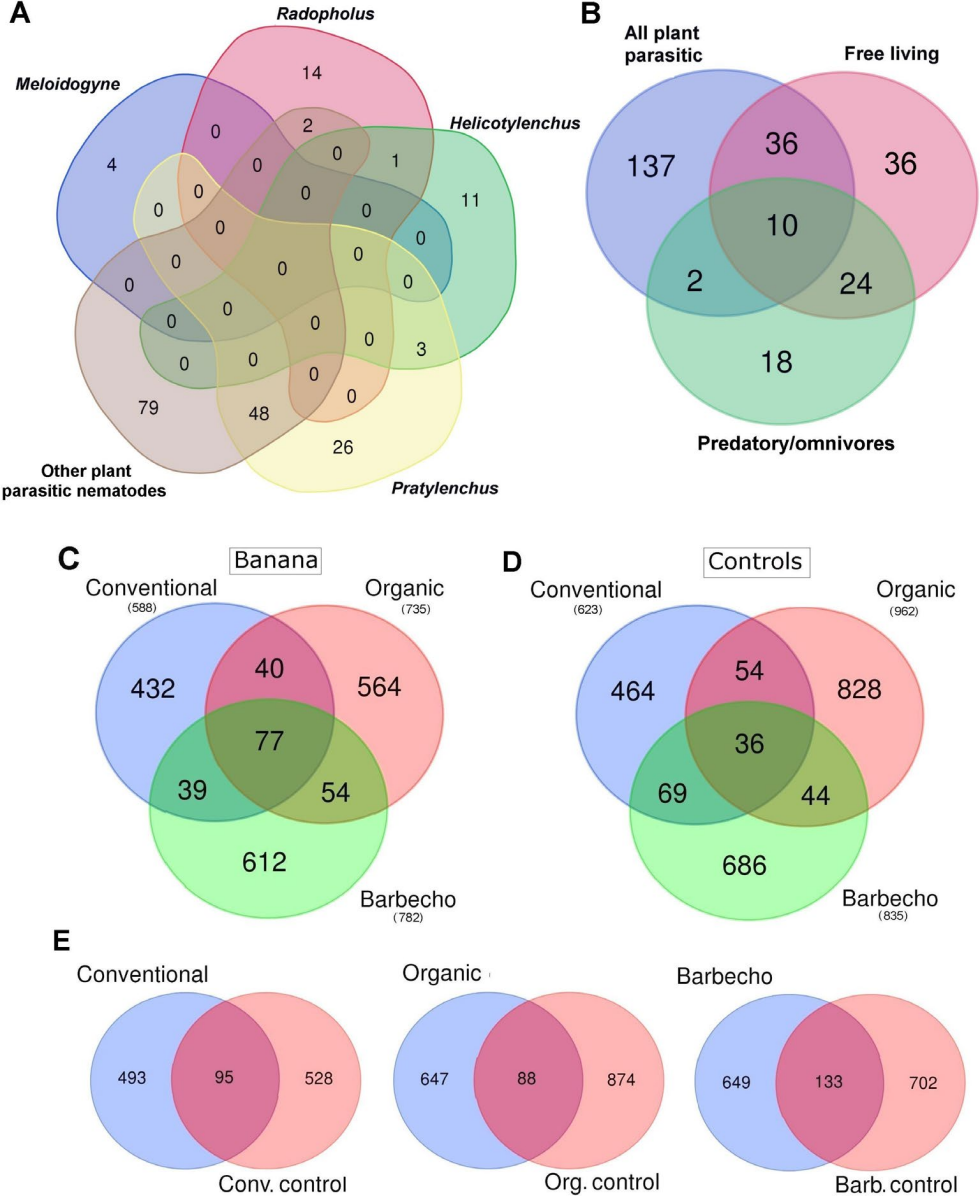
Venn diagrams for the fungal and bacterial taxa correlated with plant‐parasitic nematodes (A) and nematode feeding groups (B). Repartition of all ASV taxa in the three different farming systems for the banana rhizosphere (C) and the adjacent control samples (D) and, for each system, in the banana samples vs the corresponding controls (E).

Given ensembles of bacteria and fungi showed specific correlations with nematode guilds, and no shared microbiome was found among correlated taxa. *Pratylenchus* showed a higher number of ASV or OTUs in common only with *Xiphinema*, criconematids and only 3 ASV with *Helicotylenchus* (Figure 1A). When the nematodes were classified by feeding groups, a shared microbiome could be identified formed by 10 taxa only, with most species for the free‐living that were in common with herbivores and predatory/omnivores (Figure 1B). The shared fungi included the leaf pathogens (*Curvularia heteropogonis*), *Candida* sp., *Penicillium* sp., the root endophytes *Pseudophialophora tarda* and *Serendipita* sp., with two further unclassified taxa. The shared bacteria included only unclassified members of Microbacteriaceae, Enterobacteriaceae and Rhizobiales.

### 3.2 Bacteria Metabarcoding

A total of 3633 ASV were obtained from all samples after filtering the entries without classification at the phylum and upper level, those unassigned and those identified as “mitochondria” or “chloroplast”, and those whose total sequences sum (all samples) was ≤ 30, finally joining the sequence numbers for some redundant species. The unclassified bacterial entries included a total of 3025 ASV (83.2%). The three banana farming systems showed a total of 1818 ASV (Figure 1C). The shared microbiome included 77 ASV (4.2% of total), with 18 unclassified genera and 37 unclassified species mostly belonging to Proteobacteria (50.6%, with 24 unclassified), Actinobacteria (16.9%, with 2 unclassified) and Firmicutes (Bacilli, 13%, with 3 unclassified) (Table S2A).

Metabarcoding data showed an effect of the farming systems and the banana cultivation. The organic and barbecho farming systems were characterised by a higher richness and diversity of ASV, with more than 40% of the total that were present in each system, whereas a lower frequency was found in the conventional samples (32.3%, Figure 1C).

We also examined the controls, to check for an effect of the farming system independent of the presence of the banana plants. The control samples, collected from grasses in sites adjacent to the banana plants but without banana roots, showed a total of 2181 ASV. Consistently with the banana samples, also the organic and barbecho farming systems controls showed a bacterial diversity higher than that of the conventional system. The highest number of ASV was found in the organic control samples (44.1% of total, Figure 1D). However, the shared microbiome of controls was lower, when compared with the banana plants, with only 36 common ASV (1.6% of total, with 8 genera and 17 unclassified ASV). The control samples, whose nematode populations included two *Xiphinema* spp., showed presence of *Ca*. ‘Xiphinematobacter’ (phylum Verrucomicrobia), a bacterial lineage of endosymbionts of *Xiphinema* spp. No *Xiphinema* sp. was found in the banana samples nor any *Ca*. ‘Xiphinematobacter’ was present in the corresponding microbiomes (Table S2B).

The shared microbiomes of the three banana (77 taxa, Figure 1C) and the three control groups (36 taxa, Figure 1D) shared only 17 ASV in common, considered as ubiquitous, including, among others, *Priestia* (*Bacillus*) *megaterium* and *Nitrospira calida*, with seven unclassified taxa (Figure S1A, Table S2C). When each banana system was compared with its corresponding control, the barbecho samples were the most uniform, showing the highest number of shared ASV (Figure 1E).

#### 3.2.1 Effects of Banana Cultivation

**FIGURE 2:**
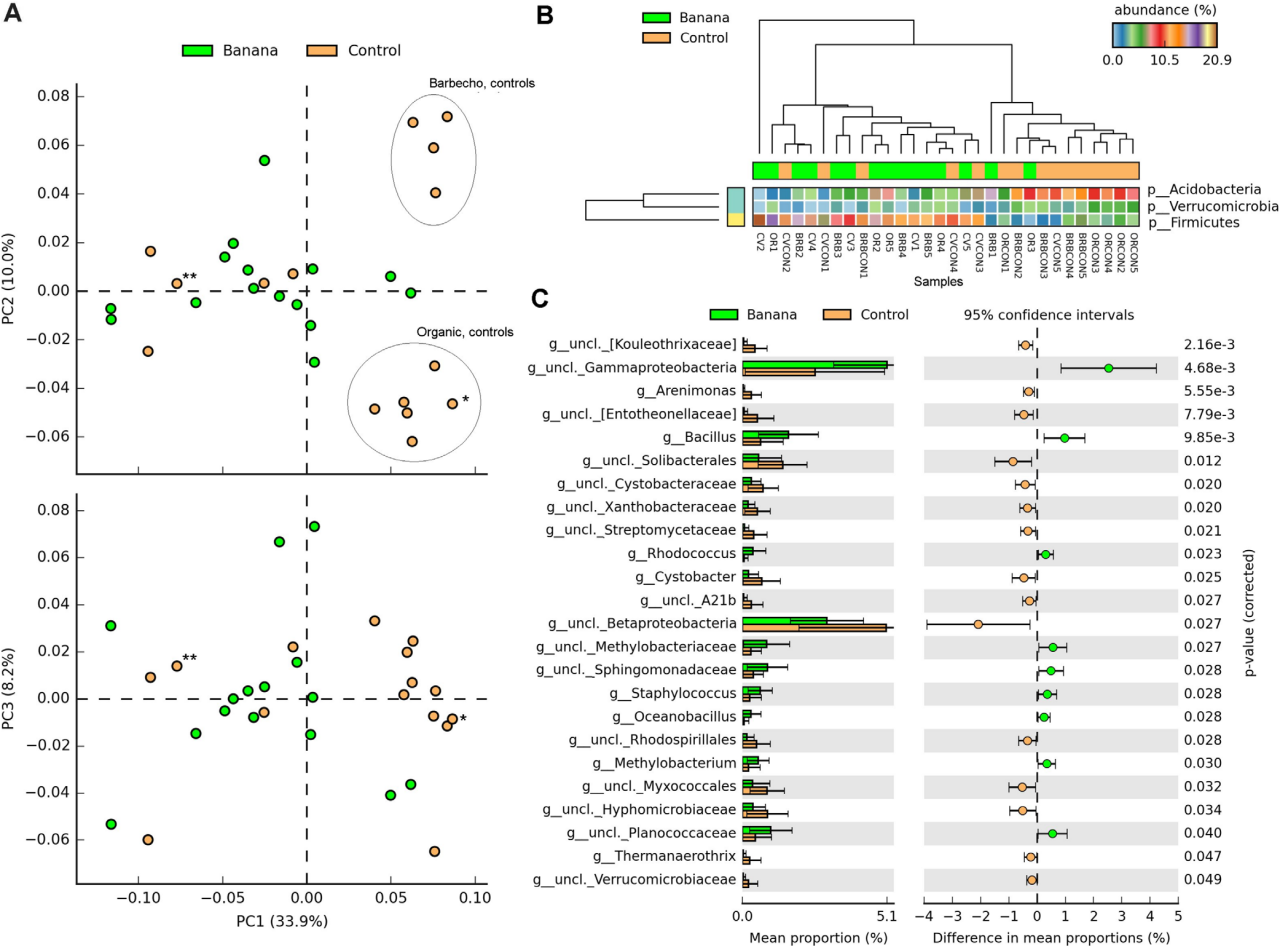
Samples representation, based on 16S rRNA gene ASV, considering cultivation (all banana samples vs all controls, species level), on the first two PCA plans (PC, principal component, with % of variance). Plots show the separation of organic and barbecho controls from the banana plant samples (green), with the exception of a barbecho (**) and a conventional (*) control samples (A). Heatmap showing phyla significantly different among all controls and banana samples (B). Unclassified taxa most differentially represented among control and banana samples, at the genus level (C, uncl. = unclassified).

We compared all the banana versus the control samples. The cultivation showed an effect on the rhizosphere bacterial profiles that differed from those of the controls. PCA showed a separation of the control samples (more evident at the genus or species level), whereas no clear separation could be found among the different banana crop samples. The three first PCA axes, explaining 52.1% of variance, distinguished most organic and barbecho controls from the other samples, whereas most conventional controls grouped with the banana samples (Figure 2A). The differences between the banana and control samples involved a higher representation, in the banana samples, of Actinobacteria, Firmicutes, and unclassified Gammaproteobacteria, whereas the controls showed other, more abundant and diverse taxa (Table 1, Figure 2B,C).

**TABLE 1:** More abundant ASV taxa in the banana and the control samples.

Banana	Control
Alphaproteobacteria ( *Methylobacterium mesophilicum* ) Actinobacteria (*Rhodococcus* sp.) Firmicutes (Bacilli: *Bacillus* spp., unclassified Planococcaeae, *Staphylococcus saprophyticus, Oceanobacillus* spp.) Gammaproteobacteria (Enterobacteriaceae, unclassified)	Acidobacteria (unclassified Solibacterales) Actinobacteria (unclassified Steptomycetaceae) Alphaproteobacteria (unclassified Rhodospirillales and Hyphomicrobiaceae) Betaproteobacteria (unclassified A21b) Chloroflexi Deltaproteobacteria ( *Cystobacter fuscus* , unclassified Entotheonellaceae, Cystobacteriaceae and Myxococcales) Gammaproteobacteria (*Arenimonas oryzeterrae*) Verrucomicrobia

The top 10 taxa followed this repartition, showing a higher representation of Actinobacteria, Bacilli, and Gammaproteobacteria in the banana rhizosphere, whereas the Betaproteobacteria were more common among the controls, both at the class and family levels (Figure S2A,B). These taxa, however, were all unclassified at the genus level (data not shown). The top 10 taxa also showed a higher abundance of Bacilli in each farming system when compared with its corresponding control, a higher frequency of Betaproteobacteria in the organic control and of Planctomycetia in the barbecho control (Figure S2C).

No difference was found for the ASV diversity indices among the sample groups, except for the number of individuals that differed when comparing the conventional banana vs the organic control (Tukey's pairwise comparisons, *p* = 0.034; means = 7627 vs 14,395, respectively). However, comparing samples by treatment (field type) showed several diversity indices in the conventional management that were significantly lower than those of the organic and barbecho systems (Table S2D).

PERMANOVA showed that cultivation induced significant differences (FDR, *p* ≤ 0.05, all banana samples vs. all controls). Other significant differences were shown for the organic control versus both the barbecho control and the conventional banana samples, for the densities of other plant‐parasitic nematodes (*Xiphinema* spp., criconematids) and for the presence/absence of omnivorous/predatory nematodes (Table S3). Few significant differences were shown by PERMANOVA for pH and soil P content, including an effect of acidic vs neutral pH (not significant after FDR application), and an effect of high versus low P content (*p* ≤ 0.006) (Table S3).

#### 3.2.2 Effect of Nematode Guilds

We compared ASV abundance in samples by considering the nematodes and their density levels. Most differences were found for the omnivore/predatory nematodes and other ectoparasites (*Xiphinema* spp., criconematids). The bacteria differentially represented at the family or genus levels between samples grouped by omnivore/predatory nematodes were members of Enterobacteriaceae, Gemmataceae, Thermogemmatisporaceae, Bacillaceae and further unclassified taxa. Significant differences could not be found for *R. similis* , *Meloidogyne* and *H. multicinctus* . The other ectoparasites (*Xiphinema* spp., criconematids) showed members of Enterobacteriaceae, Rhodobacteraceae, Microbacteriaceae, Fusobacteria (*Psychrilyobacter* sp.), *Bacillus* sp. and other unclassified taxa in low density samples (Table S4). Members of Bacilli were associated with low density/absence of *Xiphinema* and criconematids, and high density/absence of *H. multicinctus* . Low frequencies of Bacilli were also associated with low numbers of omnivorous/predatory nematodes, whose absence was associated with a higher representation of Gammaproteobacteria (Figure S2D–F).

**FIGURE 3:**
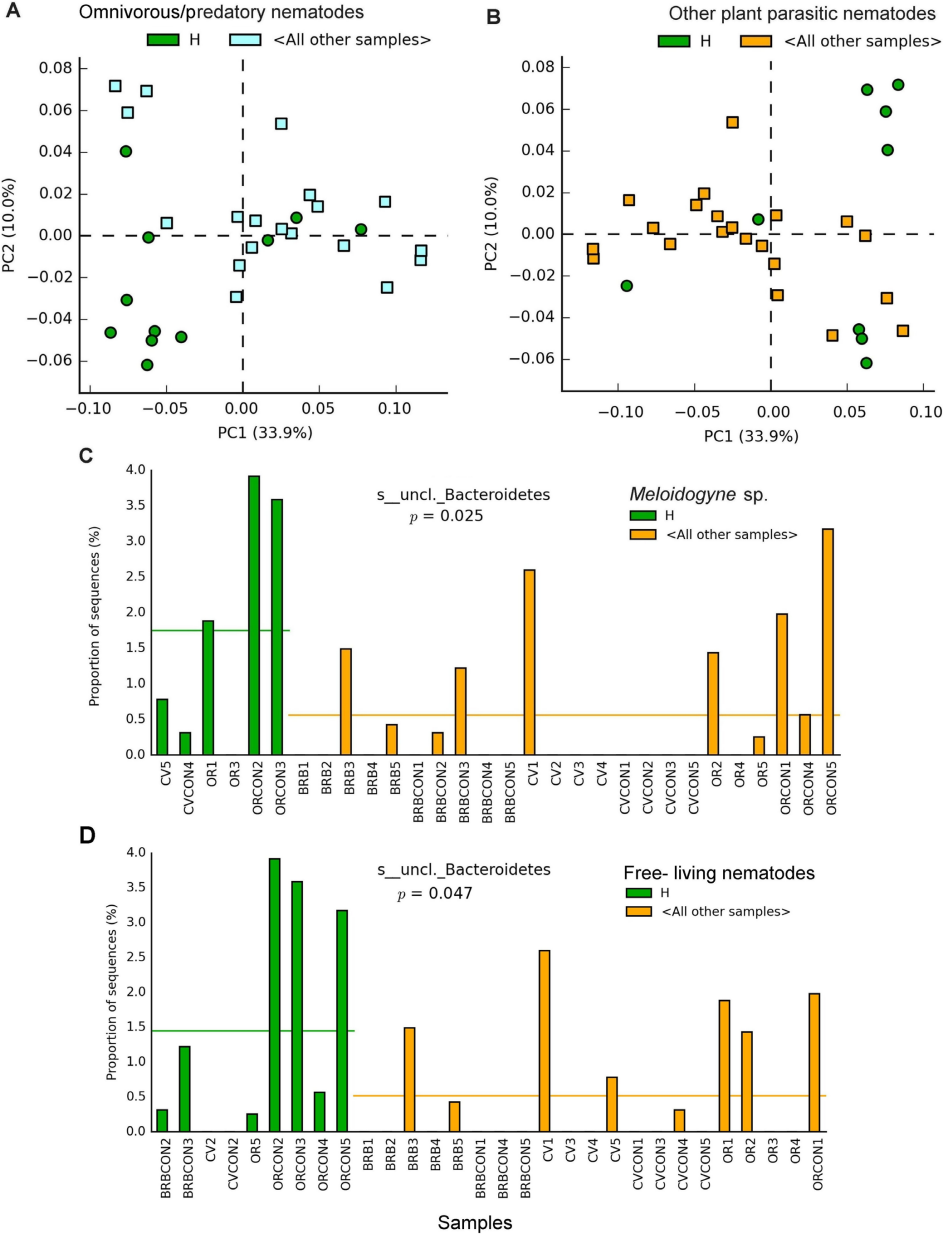
PCA of the 16S rRNA gene ASV sample profiles, classified at the genus level by nematode groups, on the first and second components plan (PC, principal component, with % of variance). Plots separate a subset of samples with high numbers of predatory nematodes (A), and those with highest densities of other plant‐parasitic species (criconematids, *Xiphinema* spp.) (B). Samples with high densities of *Meloidogyne* sp. J2 and free‐living species (nematodes 100 mL soil^−1^) showed a differential representation for an unclassified *Bacteroidetes* (phylum Chloroflexi) (C, D). H = high density, above the 10% mean confidence interval. Samples tags: BRB = barbecho; CV = conventional; OR = organic; CON = control.

PCA showed an effect of nematodes on the samples ASV profiles, most evident at the genus level when grouping samples with high numbers of omnivorous/predatory species. This group included all organic controls with only a conventional banana sample (Figure 3A). Similarly, the microbial profiles of samples with higher densities of criconematids and *Xiphinema* spp. distinguished the barbecho controls from the other groups (Figure 3B). The first PCA axes could not separate samples in relation to free‐living or *Meloidogyne* J2 densities. However, a difference could be found in the samples with *Meloidogyne* for an unclassified *Bacteriodetes* sp., more represented in samples with high densities of J2. This taxon was also more represented in samples with high densities of free‐living or omnivorous/predatory nematodes (Figures 3C,D and S3B,E).

Different effects were observed for a number of bacteria when classifying the samples by nematode feeding groups and density. Most differences for *R. similis* were found for a few ASV, including *Petrimonas* sp. and two unclassified taxa (EW055 and a *Candidate* phylum Dormibacteraeota, formerly ad3 clade), found in the samples with highest numbers of burrowing nematodes. The samples in which *R. similis* could not be detected showed the occurrence of unclassified Xantomonadaceae (Figure S3A). A different set of bacterial taxa was instead more represented in relation to the other plant‐parasitic nematodes (*Xiphinema* spp. and criconematids), with a higher abundance of Gemmataceae at high densities, and of *Sphingomonas*, *Bacillus* spp. and Enterobacteriaceae in samples with a few nematodes or none (Figure S3D). No difference in bacterial abundance was found in relation to the density of *H. multicinctus* .

#### 3.2.3 Effect of Farming

A higher bacterial diversity was found in both the barbecho and the organic management systems, compared with the conventional farming, as shown by that is, the number of taxa and individuals, or the Shannon, Margalef and other indexes (Table S2D). This effect was less evident when comparing the banana and the control samples separately. However, the barbecho and organic controls showed a higher diversity and abundance of ASV communities (observed features), although only the organic control was significantly different for individuals from the conventional crop (*p* = 0.034, Table S2D).

**FIGURE 4:**
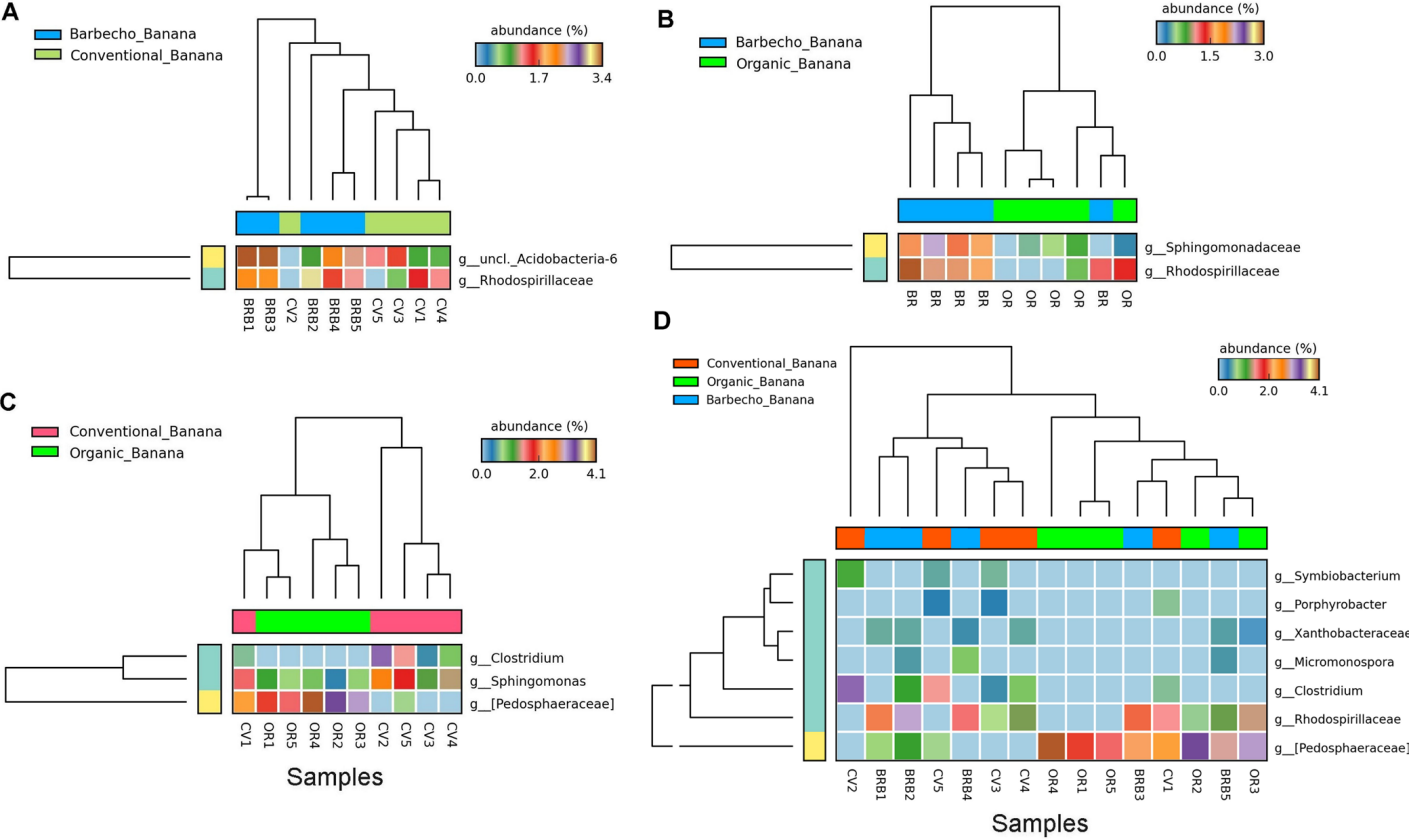
Heatmaps showing the abundance of bacterial taxa differentially represented at the genus level, when comparing barbecho vs conventional (A), barbecho vs organic (B) and conventional vs organic (C) banana crops. Differential abundance of ASV in the three banana farming systems, at the genus level (D, effect size = *η* ^2^).

PERMANOVA confirmed that the conventional management differed from both the barbecho and the organic systems (*p* = 0.047 and *p* = 0.019, respectively) (Table S3). However, few differences in taxa representations were found when comparing the banana sample groups of the three farming systems with each other, including that is, unclassified genera of Acidobacteria, Rhodospirillaceae, Sphingomonadaceae, Pedosphaeraceae, *Clostridium* or *Sphingomonas* spp. (Figure 4A–D). Organic banana had a higher abundance of Verrucomicrobia and Cyanobacteria, unclassified at the genus level, with those under barbecho enriched in Actinobacteria (*Micromonospora* sp.), whereas conventional plants showed a higher abundance of Clostridia (Figure S1C).

**FIGURE 5:**
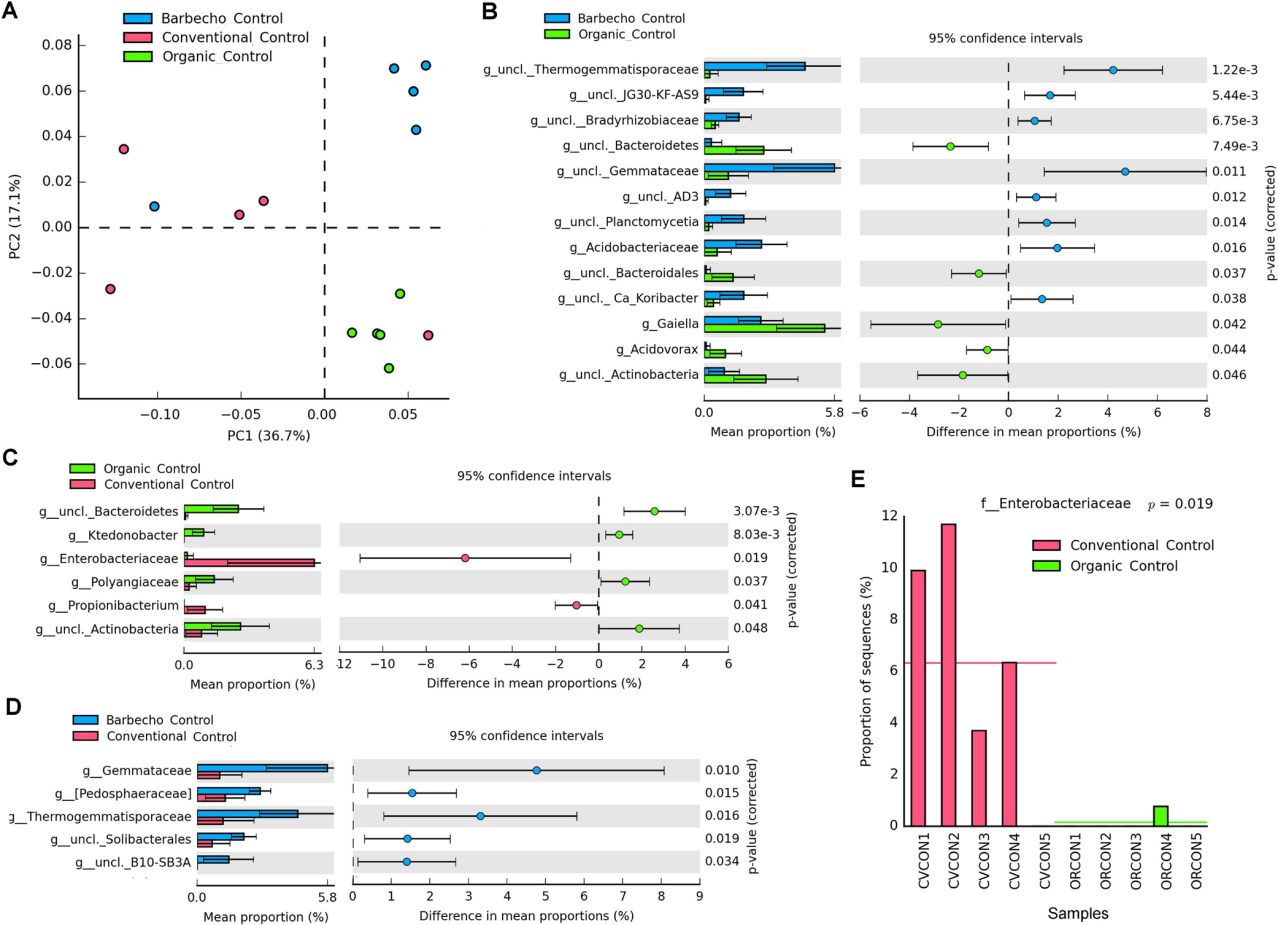
Separation of the three farming systems control samples on the first 2 PCA axes (A). Differential abundance of ASV sequences in the comparison of barbecho vs organic controls (B), and in the conventional vs organic (C) or barbecho controls (D). Frequency of unclassified Enterobacteriaceae between the conventional and organic controls (E).

We also compared the controls among them. PCA clearly distinguished the three groups on the first two axes (accounting for > 50% of variance) (Figure 5A). Significant differences involved a higher number of ASVs including that is, members of Enterobacteriaceae, Gemmataceae, or [Pedosphaeraceae]. The barbecho controls showed a higher abundance of taxa when compared with both the organic and the conventional controls. Main exceptions included unclassified Bacteriodetes, unclassified Actinobacteria, and *Gaiella* sp., more abundant in the organic than the barbecho controls, and an unclassified Enterobacteriaceae, more abundant in conventional controls (Figure 5B–E).

**FIGURE 6:**
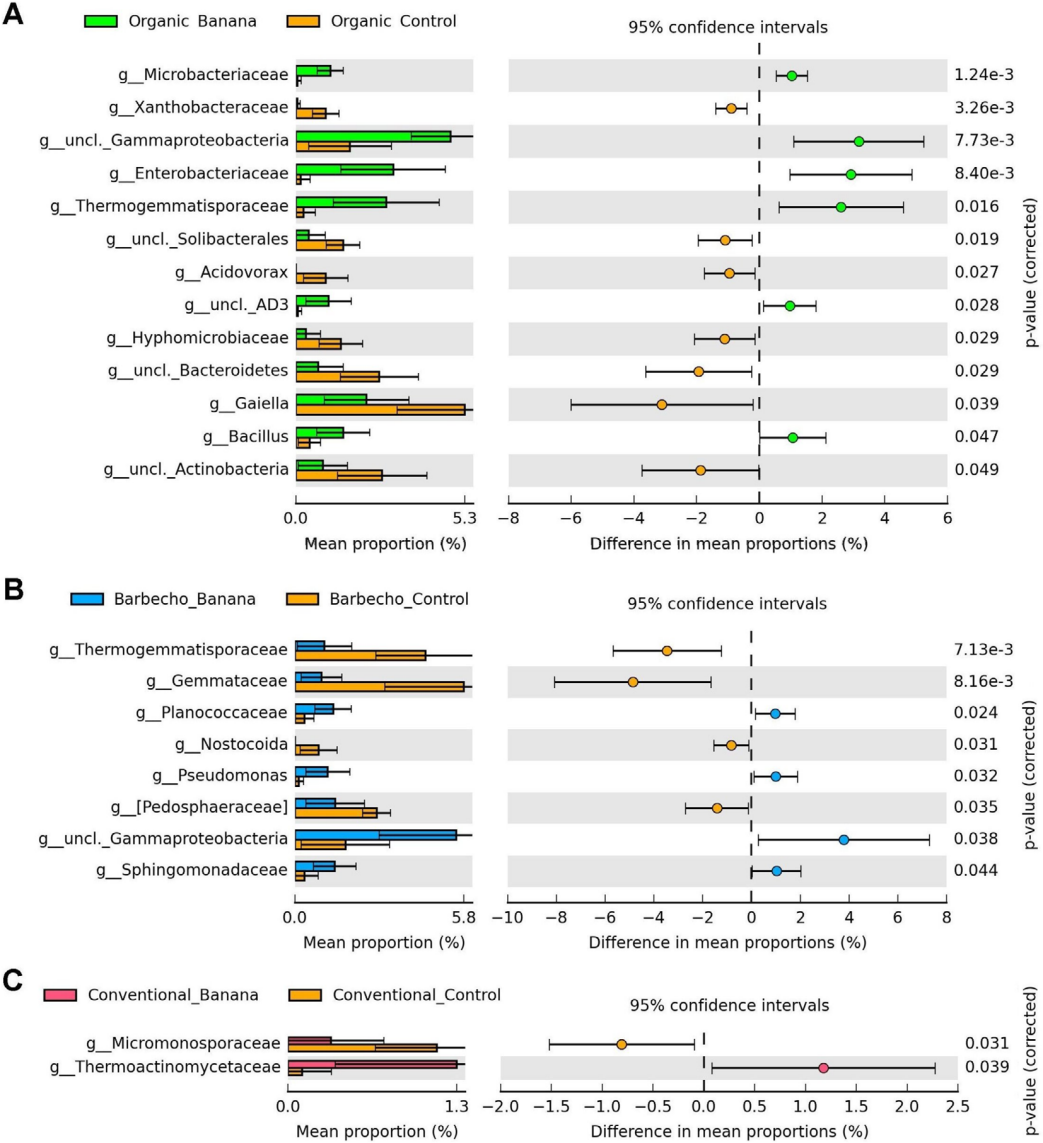
Differential representation of ASV when comparing the organic banana (A), barbecho (B) and conventional crops (C) with their corresponding controls at the genus level.

**FIGURE 7:**
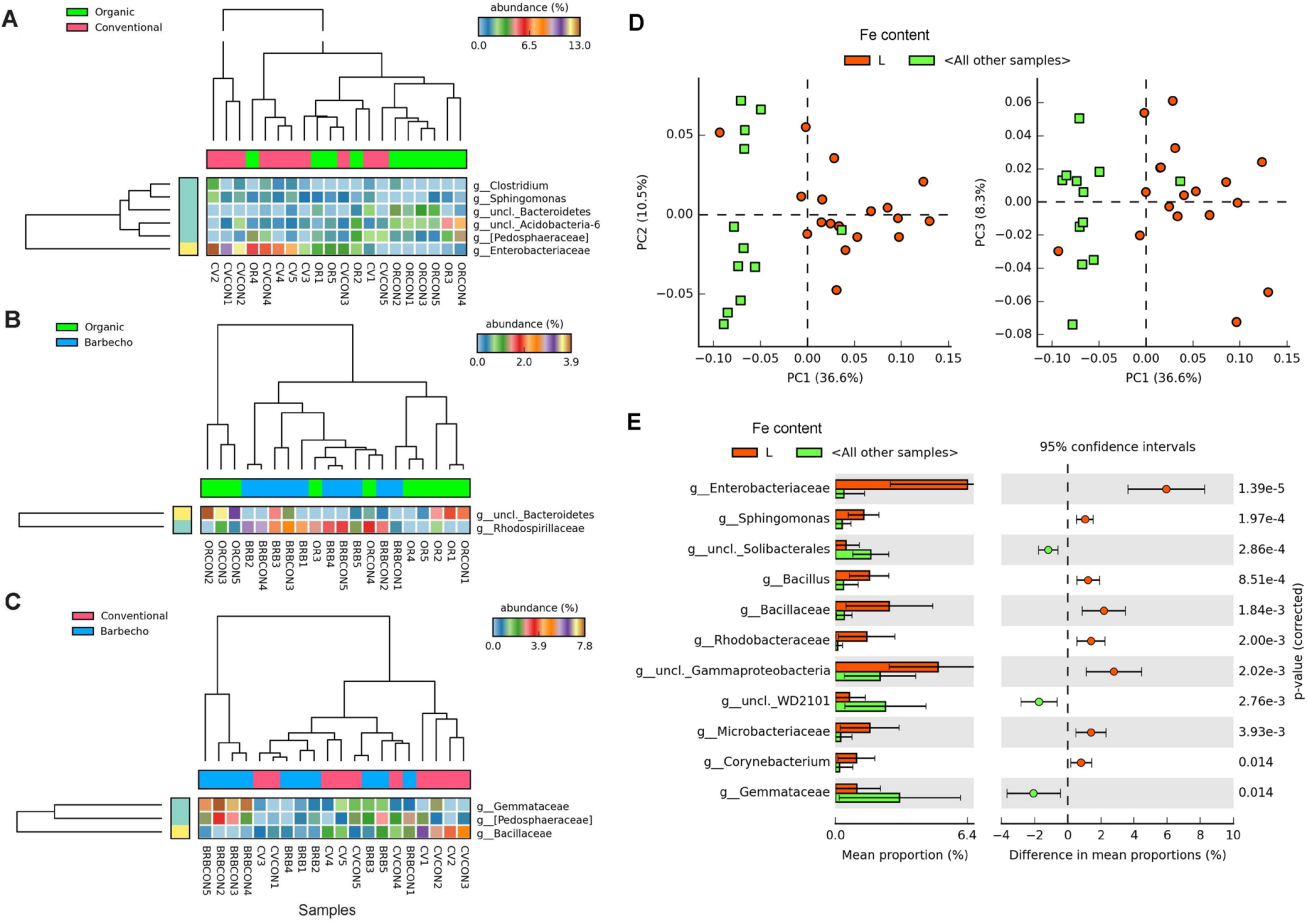
Differentially represented ASV when comparing samples pooled by farm management system, for organic vs conventional (A), organic vs barbecho (B) and conventional versus barbecho (C). Representation of samples classified, at the family level, by their Fe content (D) using the first three PCA axes (L = lower than 80% of mean, 77.6 ppm), and corresponding error bar plot of ASV, at the genus level (E).

We also compared the banana with their corresponding control samples. Barbecho and organic banana samples showed a differential representation involving a higher number of ASV, indicative of an effect due to the cultivation system, less evident for the conventional farming system (Figure 6A–C). The comparisons by management (pooling all samples by farming system) showed a higher number of ASV differentially represented between organic and conventional systems, mostly involving taxa such as *Sphingomonas* sp. and unclassified Bacteriodetes, Acidobacteria and Pedospheraceae, more abundant in the organic field (Figure 7A–C).

#### 3.2.4 Effect of Soil Properties

When the samples were classified by their soil elemental composition, a significant effect on the bacterial profiles was observed only for the Fe content, with a clear separation of the samples with a low Fe content (< 77.6 ppm, 80% of mean) on the PCA plots (Figure 7D). All the other elements did not allow any clear PCA separation, at low or high elemental contents. The most represented ASV at low Fe content were unclassified members of Enterobacteriaceae, Gammaproteobacteria, and Bacillaceae, together with species of *Bacillus*, *Sphingomonas*, and *Corynebacterium* (Figure 7E).

Soil pH showed only a few effects on the ASV representation. Only acidic soils (pH range: 5—5.5) showed a higher frequency of a few taxa (Gemmataceae, Thermogemmatisporaceae, Acidobacteriaceae, and an unclassified JG30‐KF‐AS9). Similarly, the soil P content also had a minor effect, as indicated by NMDS that showed only a separation of the organic control samples from the others (Figure S3F). High P levels positively affected an unclassified *Bacteriodetes* and a member of Actinobacteria, with an opposite effect on an unclassified member of Enterobacteriaceae (Figure S2G).

### 3.3 Metabarcoding of Fungi

The ITS1‐2 raw sequence data were used to produce OTUs because the ASV classifications previously obtained did not yield enough resolution to characterise the fungal diversity and its distribution among samples (data not shown). A total of 936 fungal OTUs were obtained from all samples, except a poorly representative barbecho control that was filtered. The redundant OTUs that were classified at the same species or hypothetical species (sh) level were also joined. The OTUs were then filtered below an all samples sum < 5 sequences. The 620 OTUs that were unclassified at the species level (66.2% of total) were kept in the database using the “SH” or “unclassified” code for identification, with 561 OTUs (59.9%) showing the term “*incertae sedis*” at the genus level. The OTUs tagged with this term were 132 (14.1%), 324 (34.6%), 349 (37.2%) and 490 (52.3%) at the phylum, class, order, and family levels, respectively.

**FIGURE 8:**
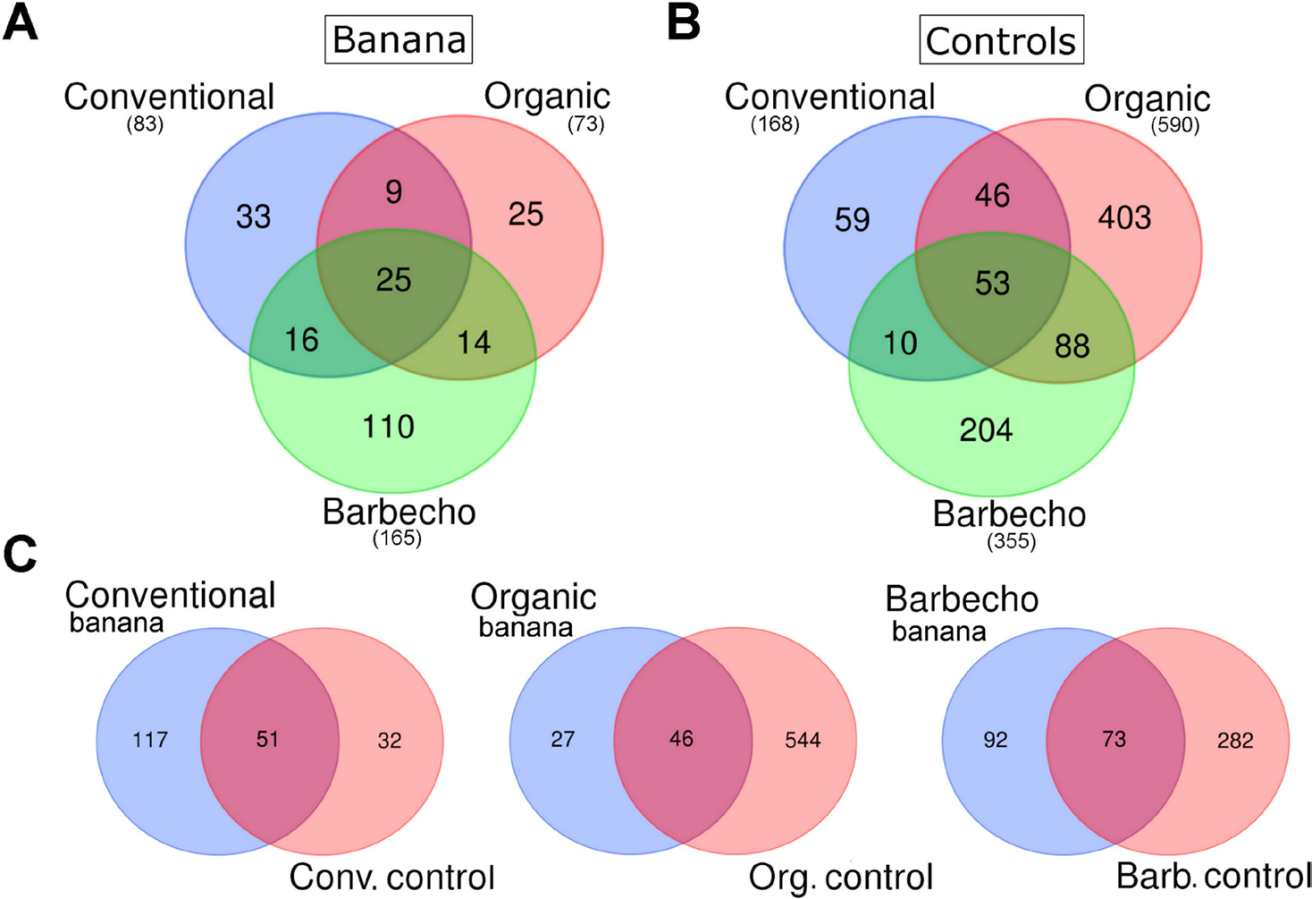
Venn diagrams showing the fungal OTUs repartition in the three different banana farming systems (A), in their adjacent control samples (B) and when compared with their control in each farming system (C).

The three banana farming systems showed a total of 232 fungal OTUs. The barbecho cultivation system included most OTUs (71.1% of total), whereas lower and almost equivalent frequencies were found for the conventional and organic crop samples (35.7% and 31.4%, respectively) (Figure 8A). The control samples had a higher richness of taxa, with a total of 863 OTUs. The organic and barbecho controls included more OTUs (68.3% and 41.1% of total, respectively) than the conventional control samples (19.4%) (Figure 8B). The whole organic farming system (including both the banana and control samples) showed the highest number of OTUs (*n* = 617), followed by the barbecho (*n* = 447) and conventional systems (*n* = 200, Figure 8C).

Taxa summaries showed that farming and management had the most significant effects on fungi, whereas *Radopholus* and the other plant parasites (*Xiphinema* sp., criconematids) showed most effects among nematodes. Samples with low numbers of *Meloidogyne*, omnivorous/predatory, and *H. multicinctus* were associated with a few fungi, such as *Hyphodontia setulosa* (Agaricomycetes), members of Eurotiomycetes, and *Arachnion* sp., respectively. Samples with high numbers of *Radopholus* were associated with *Hyphodontia* and *Phaeotremella*, whereas the highest levels of free‐living nematodes showed a higher representation of Pleosporales. Samples with high levels of the other plant parasites showed higher frequencies of unclassified Onygenales (*inc. sedis*) and Clavariaceae (*inc. sedis*), whereas *Trichoderma* and *Cladosporium* spp. were more represented in samples with low or no nematodes (Table S5).

The fungal shared microbiome of the banana rhizosphere samples was composed of 25 OTUs (2.6% of total), including *Alternaria prunicola*, *Aspergillus glabripes*, *A. destruens*, *A. penicillioides*, *Candida* sp., *Cladosporium cladosporioides*, *C. herbarum*, members of the *Fusarium oxysporum* complex, *Malassezia globosa*, *M. restricta*, *Penicillium* sp. (closest: *P. polonicum* ), *Trichoderma asperellum*, *T. lixii*, *Ulvella* sp., and *Umbelopsis* sp., with 5 further unclassified OTUs. The shared microbiome of the control samples showed a higher number of OTUs (53), of which 18 were in common with the banana shared microbiome. These ubiquitous genera included *Alternaria*, *Aspergillus*, *Cladosporium*, *Candida*, *Fusarium*, *Trichoderma*, *Malassezia*, and others, unclassified (Table S6A–C).

Few differences were found for the diversity indices among the sample groups, mostly found when comparing the number of taxa of the organic control with both the conventional and the organic crop, and with the conventional control. Other differences were found for the higher Margalef, Fisher alpha, and Chao‐1 indices of the organic control versus the organic and the conventional systems (Tukey's pairwise comparisons, *p* < 0.05) (Table S6D). No difference was found for the diversity indexes among the three farming systems, except the number of individuals, highest in barbecho and lowest in the conventional system (Table S6D).

PERMANOVA pairwise comparisons showed an effect of cultivation (banana vs. controls) and type of management (barbecho vs conventional crops) on the fungal OTUs. Further, less significant differences were found by comparing the barbecho control versus the two other controls and the conventional crop. The presence/absence of omnivorous/predatory nematodes and other plant parasites (*Xiphinema* spp. and criconematids) also showed some effects. Only one significant difference was shown by PERMANOVA for pH, when comparing samples with acidic versus neutral pH, whereas no significant difference was found among samples for the P content of soil (Table S7).

**FIGURE 9:**
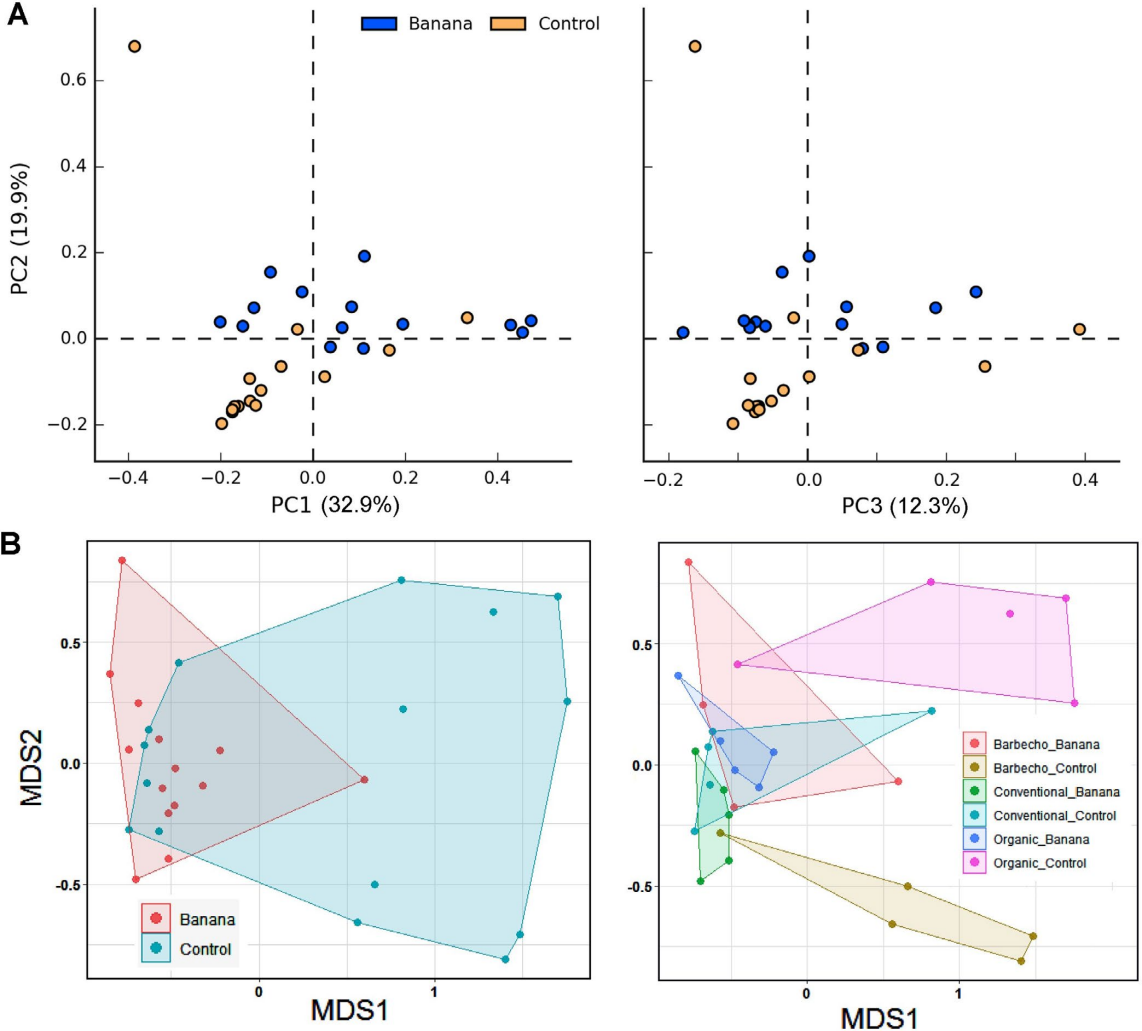
First two PCA plans showing the overlap of the pooled samples when comparing banana and control fungal OTUs data (A). NMDS first plan for banana and management system samples compared with controls (B).

The PCA plans showed a partial overlap for samples classified as banana crop or controls, at the family and genus levels, with most control samples clustering together on the first two PCA plans (Figure 9A). A clear sample separation was shown by NMDS, but mostly among controls (Figure 9B). The other samples overlapped on the PCA plans when classified by the three different farming systems and crop management, or by all the other variables related to nematodes (data not shown).

#### 3.3.1 Effect of Cultivation

**FIGURE 10:**
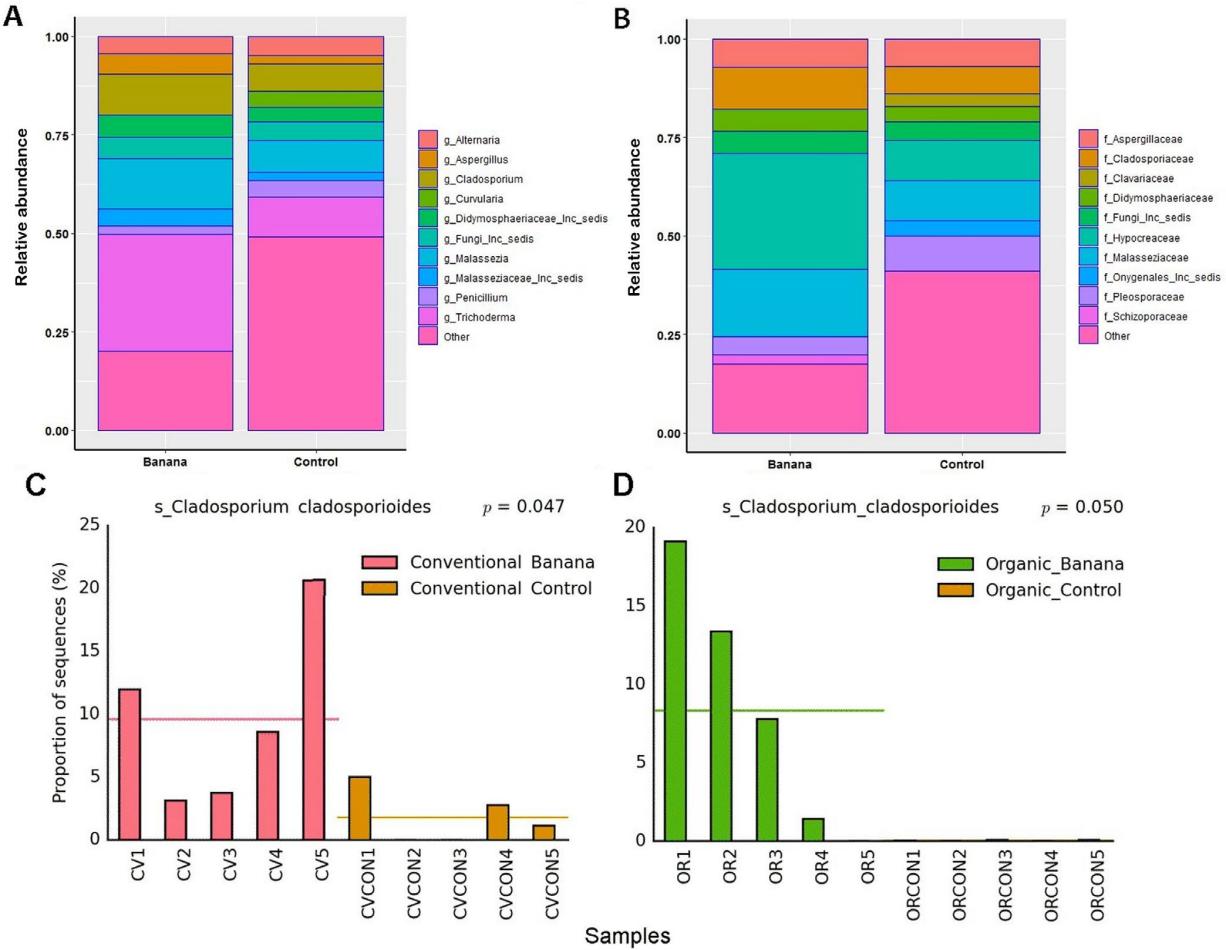
Top 10 taxa observed when comparing banana with control samples, at the genus (A) and family (B) levels. Relative abundance of *Cladosporium cladosporioides* in conventional (C) and organic (D) banana rhizosphere, compared with the corresponding controls.

A number of antagonistic and biocontrol agents such as *Metarhizium* and *Trichoderma* were among the OTUs differentially represented when comparing the banana vs the control samples. *Trichoderma* spp. were more frequent in the banana rhizosphere, with the common saprotroph *Cladosporium* and the opportunistic skin parasite *Malassezia* sp. also more abundant in the organic banana rhizosphere (Figures 10A–D, S4A–C). Organic banana also showed a higher representation of unclassified members of Phaeotremellaceae, Symbiotaphrynaceae and Rozellomycota (Figure S4D). No significant difference was found for the nematophagous fungi found in samples, such as *Arthrobotrys paucus*, *Dactylella leptospora*, *Dactylella* sp. and *Drechmeria campanulata*. A higher abundance of Chytridiomycota was instead found among the controls, together with Hypocreaceae, represented by *Trichoderma* sp. (Figure S5A). Chytridiomycota were also more abundant in barbecho compared with conventional samples, with a higher representation of Archaeorhizomycetes and Laboulbeniomycetes (obligate fungal parasites of arthropods), as well as members of Onygenales (keratin‐degrading and animal pathogens). An unclassified member of Dydimosphaeriaceae was more prevalent in the conventional banana (Figure S5B).

A number of Basidiomycota (Tremellomycetes) and Ascomycota (including unclassified taxa) were differentially represented when comparing all organic versus all conventional samples at the class level. Members of Ascomycota were also enriched at the family and genus levels. Members of the *Fusarium oxysporum* complex were more represented in organic samples, whereas members of *Malassezia* (*M. restricta*) were more frequent in the conventional ones (Figure S5C). Organic samples showed a higher frequency of Tremellomycetes when compared with barbecho. Barbecho showed a higher abundance of Archaeorhizomycetes and Laboulbeniomycetes, with unclassified Onygenales and Pyxidiophorales (Figure S5D).

The organic system showed a higher abundance of Sordariomycetes, in particular for members of Hypocreaceae and *Trichoderma*, compared with its corresponding control (Figure S5E, Additional file 3). Unclassified Capnodiales, a group of sooty mould fungi, was enriched in the organic and conventional banana, whereas the genus Archaeorhizomyces was more represented in the barbecho control (Figure S4E–G).

The controls from the barbecho and conventional farming systems showed a differential abundance of fungal profiles and were clearly separated on the three PCA axes (Figure S6A). Comparing each banana crop management with its controls showed a difference only for barbecho, with a higher representation of Chytridiomycota and unclassified Archaeorhizomycetales in its control and of Sordariomycetes in the banana rhizosphere (Figure S6B). The controls of the three farming systems were then compared with each other. A higher abundance of Archaeorhizomycetales and Onygenales was found in the barbecho control than in the conventional control, which showed a higher frequency of Malasseziales, mostly *M. restricta* (Figure S6C). Comparing the organic with the conventional controls showed a higher frequency of Ascomycota, Pezizomycotina, and Tremellales in the former, with a higher representation of members of the *Fusarium oxysporum* complex (Figure S6D). The barbecho control showed, when compared with the organic control, a higher representation for a few taxa including unclassified members of Onygenales and Archaeorhizomycetales (Figure S6E).

#### 3.3.2 Effect of Nematode Guilds

No effect on the fungal OTU profiles was observed in relation to the density levels of free‐living and plant‐parasitic nematodes, except for *Xiphinema* and criconematids. When compared with all the other samples, these nematodes showed, at high densities, a higher representation of Chytridriomycota, Onygenales, Nectriaceae, and *Fusarium* spp. and a significant, lower frequency of Sordariomycetes and Hypocreales, which were mostly represented by *Trichoderma* spp. (Figure S7A). High numbers of omnivores/predatory nematodes showed an increase of Mortierellomycota and Peziomycotina, with more represented taxa such as *Ulvella* sp., *Curvularia heteropogonis*, and members of the *F. oxysporum* complex. *Malassezia restricta* was more prevalent in samples with low numbers of omnivores/predators, non‐acidic pH, and medium‐low nematode densities (Figure S7B,D).

#### 3.3.3 Effect of Farming System and Soil

**FIGURE 11:**
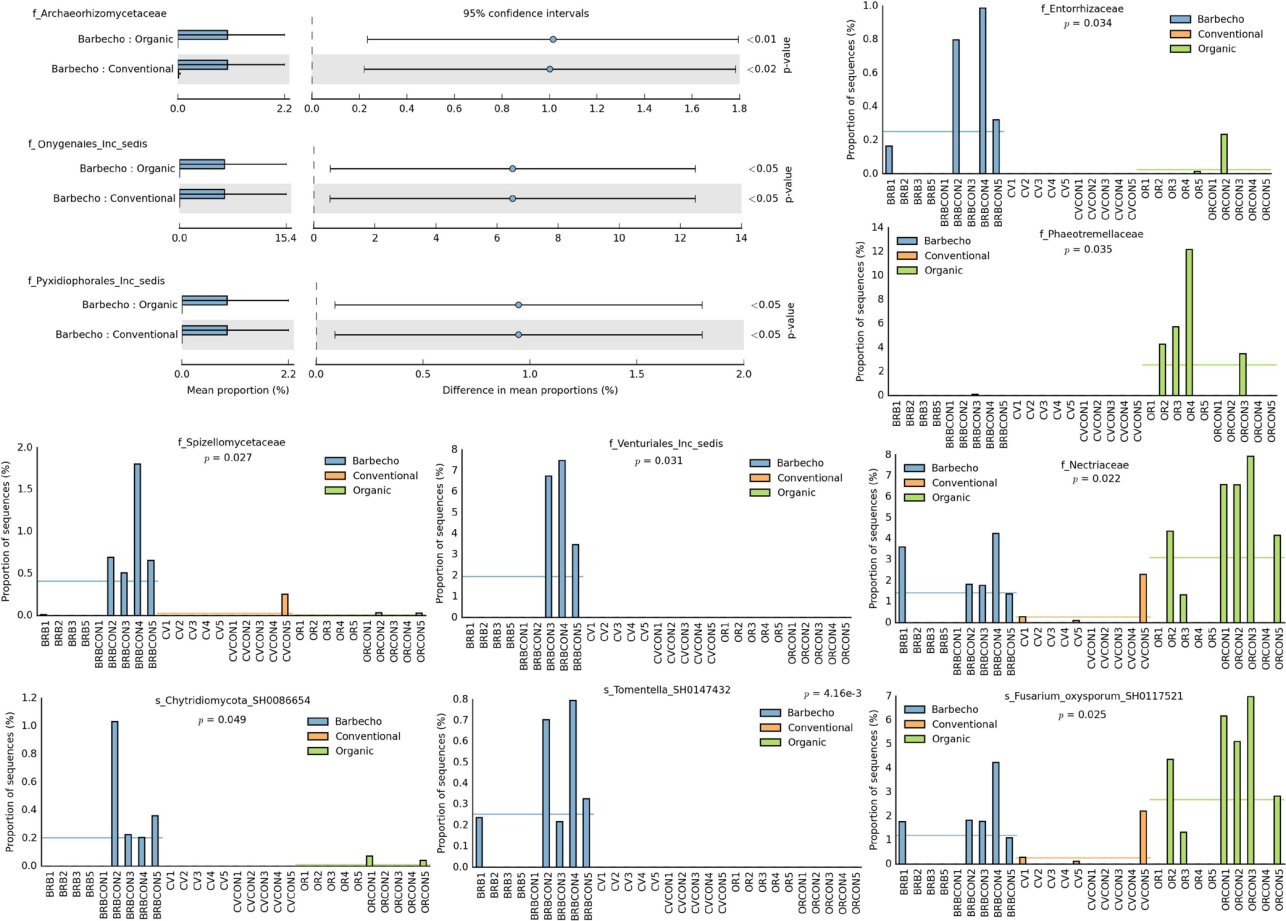
Fungal taxa enriched by farming system, at different taxonomic levels.

No difference in the fungal OTUs representation was found when the samples grouped in the three banana farming systems were compared with each other. Similarly, the control samples of the three farming systems overlapped and could not be clearly separated on the three first PCA plans (data not shown). The control samples, however, appeared to be more affected by the farming system applied, as they showed some differences for a reduced number of taxa when compared with each other. When no effect size was applied to the data set, fungi enriched by farming system were mostly observed in the barbecho control, including unclassified Chytridiomycota and the ectomycorrhiza *Tomentella* sp. Nectriaceae, with members of the *F. oxysporum* complex, were found in barbecho and organic farming systems but were almost absent from the conventional samples (Figure 11).

High P content favoured a higher representation of Pezizomycotina, Rozellomycota, and Mortierellales. *Ulvella* sp., *C. heteropogonis*, and members of the *F. oxysporum* complex were the more frequent taxa in high P samples, together with *Papiliotrema laurentii* (Figure S7C). Members of Chytridriomycota were also more represented at acidic pH, with some Onygenales and Agaricales, mostly unclassified at the lower levels, and Malasseziales (*M. restricta*), more represented at acidic soil pH (Figure S7D).

The comparison of controls showed a differential representation for a few taxa only. Members of Tremellomycetes, Sordariomycetes, and Pezizomycotina (*Ulvella*) were more represented in the organic control, whereas the barbecho control mostly favoured unclassified Onygenales (Figure S8A–D). The conventional control showed only a higher frequency of Malasseziaceae and Aspergillaceae when compared with the barbecho control, and a higher abundance of *Malassezia* when compared with the organic control. In this comparison, the barbecho control showed a higher frequency of *Ulvella* and *Fusarium* sp., with an unclassified Ascomycete (Figure S8E,F).

### 3.4 Associations of Bacteria and Fungi

The effect exerted by the farming system on the species associations was tested by comparing all the taxa in the banana and in the control samples. *Trichoderma*_SH0097788 was the most represented in both the banana crop and controls, with a mean of 6331 and 8256 sequences per sample, respectively. Filtering at 5 mean sequences per sample yielded two datasets with 299 taxa for the banana and 514 for the control samples. The correlated taxa (Spearman's *rho*), in common between both sample groups, were 34 (Figure S9A). After filtering the OTUs and ASV present in < 5 samples, the correlations calculated among taxa in the banana plants showed 160 significant scores involving 68 OTUs/ASV (86 positive and 74 inverse correlations, 7% of total, *p* ≤ 0.05) (Table S8). The correlations among taxa showed a higher species representation in the controls, with 117 OTUs/ASV and 1670 significant correlations (1042 positive and 628 inverse, 24% of total, *p* < 0.05) (Table S9A,B, Figure S9B,C). The shared taxa included mostly fungi of genera *Cladosporium*, *Trichoderma*, *Malassezia* and *Aspergillus*, and bacteria of genera *Bacillus*, *Sphingomonas*, *Staphylococcus*, *Koribacter, Ilumatobacter*, *Reyranella*, and other unclassified (Table S9C).

## 4 Discussion

Most effective factors influencing the distribution, diversity and composition of the soil microbial communities related to specific environmental traits include climate, soil properties, and anthropic effects due to plant cultivation and management (Liebig et al. 2005; Hartman et al. 2018; Singh et al. 2023; Labouyrie et al. 2024). In this study we determined, in a tropical environment, the impact of banana cultivation and management type on the rhizosphere microbiota. We also evaluated the number and composition of nematode feeding groups, together with further soil constituents. Comparisons with adjacent controls allowed the identification of effects linked to cultivation. The microclimates of the farms (located only a 1–2 km apart, within the perimeter of the EARTH University station) were similar, allowing the study of factors restricted to cultivation and management. The effect of confounding factors such as climate or extra‐farm variables appeared also minimal, due to the proximity and similarity of the fields.

### 4.1 Effect of Cultivation

PERMANOVA showed that the Gran Enana cultivation was effective on a specific, although reduced, number of bacterial taxa. Members of Gammaproteobacteria, Firmicutes (*Bacillus* spp.) and Sphingomonadaceae were more abundant in the banana rhizosphere, whereas Betaproteobacteria were more represented among the controls. This shift appears related to cultivation. A higher Gammaproteobacteria diversity was reported in response to soil pollution, linked to changes in gut microbiomes of soil invertebrates (Zhang et al. 2021). A higher diversity of these bacteria was also reported in Fusarium‐wilt infested banana fields in Costa Rica as an indicator of plant health (Köberl et al. 2017). Pesticides use, plant health and soil pollution, all linked to plant protection actions, likely explain the higher frequencies of Gammaproteobacteria observed in the banana rhizosphere.

Additionally, the abundance of specific taxa was indicative of a niche specialisation. The enrichment with Sphingomonadaceae in banana rhizosphere is consistent with a response to pesticide or fungicide stress, as these bacteria can metabolise pollutants (Leys et al. 2004). Also, the higher representation of Bacilli in the banana samples appears linked to cultivation and plant health, as these bacteria are involved in various services, such as pest control, plant health, and nutrition (Saxena et al. 2020).

Further factors such as soil chemical composition also appeared to be involved in the shift between crops and controls. An unclassified Gammaproteobacteria was more represented, together with an unclassified member of Enterobacteraceae, in samples with low Fe content (Figure 7). Iron deficiency and plant nutritional status are known to influence the rhizosphere microbiome, eliciting different metabolic strategies deployed by plants to overcome Fe shortage through that is, increased root exudation (Pii et al. 2016).

A previous study showed a higher abundance of Rhizobiales and Solibacterales in the Gran Enana rhizosphere compared with controls, enriched in Actinomycetales and Sphingomonadaceae (Ciancio et al. 2022). The bacterial microbiome of the banana rhizosphere is consistent with data from other studies that reported Proteobacteria as major components of banana cv Williams, followed by Bacilli and other lineages (i.e., ad3, *Nitrospira*, *Sphingomonas* and unclassified Bradyrhizobiaceae) (Birt et al. 2022). Most of these taxa were found in this study, except for a lower representation of Acidobacteria. Together with *Gaiella*, *Solibacter* and *Bacillus*, they appear to be ubiquitous and constitutive core members of the endo‐ and rhizosphere microbiome of field‐grown *Musa* spp. (Birt et al. 2022; Navarrete et al. 2015).

The presence of banana roots also affected the abundance of fungi such as members of *Aspergillus*, *Cladosporium*, and *Trichoderma*. The first two genera include plant pathogens, whereas *Trichoderma* includes several plant‐beneficial, endophytic and/or mycoparasitic species. Data showed hence a detectable trend in the banana rhizosphere due to cultivation, with the occurrence of pathogenic taxa and the concomitant recruitment of beneficial, antagonistic species (i.e., *Bacillus*, *Trichoderma*). This is confirmed by a higher representation of Chytridiomycota found in controls (compared with banana samples), in barbecho (compared with the conventional system), and at more acidic pH (Figure S5A,B,D). These zoosporic fungi are present in a wide range of habitats and include parasites as well as saprotrophs (Powell 2020). They are involved in nutrient release and parasitism in aquatic food webs rich in organic matter, and as such, they appear as possible indicators of microhabitat changes induced by cropping and/or fungicide treatments.

### 4.2 Effect of Farming System

Comparisons by farming systems indicated few significant differences for the bacteria mean diversity (Table S6E), observed also when comparing individual samples. Averaged indices showed the highest total number of individuals in the organic control, whereas the conventional banana crop had the lowest (Table S2D). Other studies showed that in banana plants the bacterial diversity differed mostly by the part examined, rather than by other factors such as genotypes (Birt et al. 2022). Data from other crops (wheat) also showed that cropping affects the composition rather than the richness of root and soil bacterial profiles, with taxonomically diverse microorganisms in root and soil that were differentially sensitive to cropping practices such as tillage (Hartman et al. 2018). Data from our study suggest that differences due to banana cultivation and management were mostly due to a subset of few but specialised taxa, associated either with the banana or the grass controls.

The three farming systems showed differential enrichments for taxa accounting for specific services that were affected by the practices applied. The barbecho and organic fields had a bacterial diversity higher than conventional farming (Table S2D). These differences were more evident for the controls than for the banana plants, as the controls showed the highest bacterial diversity and abundance. The fungi were instead characterised by a less diverse distribution among the farming systems, with few differences for diversity indexes differing mostly for the highest richness of species in the organic controls, however independent of the farming system type (Table S6D,E). The barbecho, however, showed an enrichment of some specific fungi, including members of the plant pathogen *F. oxysporum* complex, that was instead almost absent from the conventional field (Figure 11).

Each farming system showed an enrichment of a number of specific taxa. The organic crop was enriched in Verrucomicrobia and Cyanobacteria, whereas higher representations were found in the barbecho for Actinobacteria and in the conventional management for Clostridia (Figure S1C). Members of Verrucomicrobia reflect changes in soil fertility levels and a deficiency of nutrient availability (Singh et al. 2018). This response is consistent with the organic fertilisation used in the organic field and the lack of synthetic fertilisers. The Cyanobacteria, that include N_2_‐fixing phototrophic bacteria, are abundant in the tropics and are sensitive to the stress due to pesticides, that were absent in the organic system (Megyes et al. 2021). The Actinobacteria, involved in organic matter and plant decomposition, were higher in the barbecho field, likely resulting from the six‐year fallow period applied in this system. This is consistent with the dominance reported for several Actinobacteria in similar cropping systems that is, fallow soil of maize crops (Bao et al. 2021; Were et al. 2023). Microbial functions concerning the decomposition of organic matter were also more represented in the barbecho system, with enriched taxa involved in the decomposition of animal proteins (Onygenales) and manure (Pyxidiophorales). The barbecho system also showed the highest incidence of Rhodospirillaceae (purple non‐sulfur, photosynthetic bacteria), of unclassified members of Didymosphaeriaceae (saprotophic fungi) and of *Trichoderma* spp. (Figure 5, Table S5). The differential representation of *Trichoderma* spp. by banana cultivation and farm management is consistent with a specific association of these fungi within the plant compartments as endophytes. This observation was confirmed by the frequent isolation, in the study sites, of *T. asperellum* and other *Trichoderma* spp. endophytic in banana corms (data not shown).

Differing from the barbecho and the organic systems, the conventional field showed an enrichment in Clostridia. Members of this taxa were reported as associated with a low incidence of Fusarium wilt among banana plants, following soil disinfestation (Riva et al. 2022), consistent with the low representation of the *F. oxysporum* complex members found in the conventional field (Figure 11).

Some fungi, *Malassezia globosa* and *M. restricta*, two skin‐inhabiting parasites of mammals, showed a higher abundance in the conventional samples and were found with other Malasseziaceae also in the rizhosphere of controls (Figures 10A,B, S5C and S6C,D). *Malassezia restricta* was positively correlated with *Ktedonobacter racemifer* , a Gram‐positive aerobic species known to produce specific enzymes involved in fatty acids oxidation (Munday et al. 2016). Both *M. restricta* and *M. globosa* were reported in association with nematodes collected from forest soils (i.e., the fungal‐trichome feeder *Malenchus* spp.) or other free‐living species (Renker et al. 2003). *Malassezia* spp. were also found as main constituents of the olive seed core microbiota, likely due to a dependence on the host tissues for lipid supply (Wentzien et al. 2024). An effect of these fungi on nematodes cannot be excluded, considering that nematodes rely on saturated fatty acids and lipids as main energy reserves (Selvan et al. 1993).

### 4.3 Effects on Functional Services

A number of species involved in services such as pest/pathogen regulation or plant nutrition were found in the fields examined. Apart from Ascomycetes and Basidiomycetes, different Rozellomycota showed specific and positive correlations with nematode pests such as *R. similis* , *Pratylenchus*, omnivorous/predatory and other ectoparasitic or free‐living nematodes. Other Rozellomycota showed further positive or negative links with pH, positive correlations with other elements underpinning soil fertility such as P, and an inverse correlation with Na (Table S1C, Figure S7C). Rozellomycota are obligate pathogens of eukaryotes from soil or aquatic habitats and include poorly explored taxa found at different pH conditions, also in tropical soils (Tedersoo et al. 2017). Although their impact on plant health is difficult to quantify due to the lack of specific experimental data, their occurrence is indicative of a healthy soil condition because of their obligate parasitism. Members of Rozellomycota were also found to be more represented in the rhizosphere of banana plants in other subtropical fields (Ciancio et al. 2022). However, no distinction by cultivation and farming system could be observed for these fungi in this study, suggesting a ubiquitous trophic niche behaviour. Microorganisms involved in plant nutrition included, among fungi, unclassified members of Glomeraceae and *Rhizophagus* sp. Bacteria included members of Bradyrhizobiaceae and Rhizobiaceae such as *Rhizobium daejeonense* and *R. multihospitium* . Some unclassified Bradyrhizobiaceae showed an inverse correlation with unclassified Malassetiaceae, members of Enterobacteriaceae, and other bacteria such as *Oceanobacillus caeni* and *Blastococcus aggregatus* (Table S8). Due to the lack of taxonomic identification at the species level, the role of these Bradyrhizobiaceae in N_2_ fixation cannot be fully ascertained. All these taxa were present, however, with a low abundance, and no differential representation by farming systems could be found.

Plant nutrition and protection were the most important services provided by the bacteria and fungi encountered, but many associations appeared specific, as shown in a range of banana genotypes investigated with a metapangenomic approach (Singh et al. 2023). In spite of the lack of detailed information on the services provided by unclassified taxa, a plant‐protective function was inferred for a number of symbiotic species through their gene clusters (Singh et al. 2023). Other studies confirmed the occurrence of specific associations within the microbiomes of the banana plant compartments. In Fusarium wilt‐affected plants, the composition of the root and corm microbiota was mostly dependent on the bacteria and fungi present in the rhizosphere, suggesting a direct link of the rhizosphere microbiome with the disease insurgence and, possibly, plant health (Kaushal et al. 2020).

### 4.4 Effects of Nematode Guilds

A further factor examined in this study involved the changes in densities and composition of the nematode groups. Our basic hypothesis was that differences in the microbial profiles could not only depend on direct interactions of nematodes with associated microorganisms, but also on indirect effects such as the changes in root physiology and metabolism, induced by parasitism or nutrient cycling. Each nematode guild was in effect correlated to a specific subset of bacteria and fungi, with only a few taxa in common among the feeding groups. Moreover, no common taxa could be found when considering the herbivore species (Table S1C, Figure 1A,B). However, data indicated a minor impact of nematodes, as shown by PERMANOVA, on both bacterial and fungal profiles. An effect was found only for *Xiphinema* and criconematids, present mostly in the control samples, and for the presence/absence of omnivores, for all samples (Table S3). The values observed for the semi‐quantitative maturity index (MI, indicative of environmental disturbance) indicated that the nematode population structures did not differ significantly and showed no effect of stress factors such as pollutants or other disturbance sources, usually revealed by high MI values (Bongers 1999). Low MI levels (1–2), as those herein observed, are indicative of a higher incidence, among the nematode populations, of fast reproducing colonisers (i.e., microbiovorus taxa), related to higher nutrient amounts and reproduction rates, likely due to organic matter and banana plants fertilisation or, in general, soil fertility.

The nematode‐correlated bacteria included several unclassified species (Bacteroidetes or Verrucomicrobia), found in organic controls or in samples with high densities of free‐living or *Meloidogyne* sp. (Figure 3C,D). The phylum Verrucomicrobia includes endosymbionts living within the internal tissues of that is, *Xiphinema* spp., providing a metabolic benefit to the host (Sangwan et al. 2004). Further bacteria appeared indirectly linked to the nematode feeding activity that is, ad3 found in association with high densities of *R. similis* . ad3 was reported as a root inhabiting bacterium or as an indicator of root biomass (Vik et al. 2013; Billings et al. 2018). Its link with the burrowing nematodes appears likely due to the lesions produced by the pest during feeding. Among fungi, *Trichoderma* sp. (SH0060548) and *T. ochroleucum* were also positively correlated with *Pratylenchus* and other ectoparasites, whereas *T. asperellum* showed a link with *Helicotylenchus*. No correlation with nematodes was found for *T. lixii*, whereas a further *Trichoderma* sp. (SH0097788) showed an inverse relationship with other ectoparasites, a situation indicative of different, species‐specific behaviours within this lineage (Table S1C).

Low nematode levels usually result from applied nematicides or natural regulation. Several studies reported differences between diseased and healthy plants due to the effects of rhizosphere microbiomes on nematodes or other diseases. Diseased plants may promote the recruitment of beneficial microorganisms, whereas healthy plants may indicate an effective defence (Liang et al. 2024). Nematode pest regulation is known to occur through the activity of bacteria and fungi, that is, species of *Bacillus* and *Trichoderma*, which in this study were found in higher frequencies among the banana samples. The nematode‐microbial associations appear complex and differ from the microbial profiles of soil, being mostly characterised by a lower number of taxa, linked to different feeding groups (Pereira et al. 2024). Plant‐parasitic and free‐living nematodes were also reported to be associated with distinct microorganisms, including pathogens or endosymbionts (Adam et al. 2014; Elhady et al. 2017; Kanfra et al. 2018; Ciancio et al. 2022). This is consistent with the specificity of the rhizosphere microbiota correlated to each nematode species or feeding group found in this study. These associations involve nematode‐regulating microorganisms and predators that may have a potential for plant protection and pest suppressiveness (Elhady et al. 2018; Bent et al. 2008; Westphal and Becker 1999; Stirling 2011; Ferris et al. 2012; Giné et al. 2016; Topalović and Heuer 2019).

However, the bacterial and fungal species associated with the nematodes groups in this study appeared location‐dependent, as they differed from those found in Tenerife on the same banana *cv* (Ciancio et al. 2022). When considering the services of these species and the possible similarities between the two locations, all nematode guilds were correlated, in both sites, with biocontrol agents and plant pathogens as well, although their species profiles were different. Nematode correlations in Costa Rica also involved endosymbionts (not found in Tenerife), but no direct link with taxa providing N_2_ fixation. Although the two studies cannot be directly compared, due to different climatic, sampling time and experimental conditions analysed, the Costa Rica samples were characterised by a higher abundance of microbial and nematode correlated species. This allows an insight on the impact of the nematode and microbial interactions, considering the levels of microbial diversity that were higher in this study (Menhinick indexes = 1.5–1.9 in Costa Rica vs 0.4–0.9 in Tenerife, Ciancio et al. 2022). These links suggest a higher likelihood of rhizosphere service similarity and hence an increased system resilience and stability for the Costa Rica crops, sustained by the functional similarity of species (Eisenhauer et al. 2023). The reliance of rhizosphere stability on nematode‐microbial associations has some practical consequences, involving that is, the careful use of nematicides and requiring a risk evaluation for the temporary disappearance of functional components of the nematode communities, including guilds of beneficial species.

## 5 Conclusions

The banana cultivation and the farm management systems affected the rhizosphere microbial profiles, with changes induced on fungi and bacteria. The organic management appeared as more conservative of the rhizosphere species, showing a higher level of microbial diversity compared with the more detrimental conventional system, an effect also confirmed by the more biodiverse organic controls. Data also showed specific associations of distinct groups of microbial taxa with different nematode feeding groups. Free‐living and omnivorous/predatory nematodes were highest in the organic samples, confirming the lowest impact of this management system on belowground species. The response of rhizosphere communities to cultivation and farming type provides a useful insight into the effects of crop management systems and into the benefits that may be derived from organic farming, in terms of species conservation and rhizosphere services similarity. Our findings have the potential to increase the sustainability of actual banana farming through low‐impact soil ecosystem conservation practices, suitable to be applied both at the local and regional scales.

## Author Contributions

A.C. and L.P. conceived the research study, obtained the funds, planned and designed the field work, and performed sampling, the nematode identification and their countings. M.C. performed RNA extraction, related molecular biology work, and sequence data management. A.C., L.C.R., I.P., and M.C. analysed and interpreted the data and wrote the final manuscript. All authors reviewed, revised, and approved the article.

## Conflicts of Interest

The authors declare no conflicts of interest.

## Supporting information


**Figure S1.** (A): Venn diagram of the 16S rRNA gene ASV shared microbiomes of the banana plants and adjacent controls. (B): Frequency of ASV in the banana rhizosphere samples compared with the controls, at the family level. (C): ASV differentially represented in the three banana farming systems.

**Figure S2.** Frequency of top 10 bacterial taxa based on 16S rRNA gene ASV data comparing banana versus control, at the class (A) and family (B) level and, at the class level, for the different farming systems (C). Density of *Xiphinema* spp. and criconematids (D), *Helicotylenchus multicinctus* (E), and omnivorous/predatory nematodes (F). Effect of samples soil pH and P content on ASV (G).

**Figure S3.** Frequency of 16S rRNA gene ASV in samples classified by numbers of plant‐parasitic (A, B), free‐living (C), *Xiphinema* and criconematids (D) and omnivorous/predatory nematodes (E). NMDS plot of samples in relation to farming system and levels of soil P content (F).

**Figure S4.** Abundance of ASV for genus *Cladosporium* in banana samples (A) and in organic banana vs organic control (B, C). Abundance of unclassified Phaeotremellaceae, Symbiotaphrynaceae and Rozellomycota in organic banana (D). Frequency of ASV for unclassified members from Capnodiales in organic (E) and conventional (F) banana samples compared with controls, and of *Archaeorhizomyces* in the barbecho controls (G).

**Figure S5.** Frequency of fungal OTUs at different taxonomic levels, in samples classified by cropping (A) and farm management system (B, C). Comparison of organic banana crop versus organic control (E).

**Figure S6.** Comparisons of 16S rRNA gene ASV profiles among controls: PCA plots for organic and barbecho controls (A). Comparisons of barbecho controls with the banana samples and the conventional control samples, at different taxonomic levels (B, C). Comparison of organic controls with conventional and barbecho controls (D, E).

**Figure S7.** Fungal taxa representation at different taxonomic levels, based on the density of *Xiphinema* spp. and criconematid nematodes (A), omnivorous and predatory nematodes (B), soil phosphorus content (C) and soil pH (D).

**Figure S8.** Fungi representation comparing control of barbecho vs organic crops, at the class (A), order (B), family (C) and genus levels (D). OTUs representation by comparing conventional crop control with the barbecho (E) and the organic crop controls (F).

**Figure S9.** Spearman’s correlations (A) and correlations diagrams among ASVs and OTUs in the banana (B) and control samples (C).

**Table S1.** (A) Nematode densities from the different banana and adjacent control soils. (B) Soil physicochemical properties. (C) Pairwise Spearman’s correlations of bacteria and fungi with nematodes and other sample variables.

**Table S2.** (A): Common ASV taxa in the shared microbiome of samples from all banana farming systems, with frequencies of phyla also shown as a pie chart. (B): Common taxa in the shared microbiome of controls, with frequencies of phyla also shown as a pie chart. (C): taxa in common between the shared microbiomes of banana plants and adjacent controls, and frequencies of phyla in a pie chart. (D): Diversity indices of 16S rRNA gene ASV data per sample and farm management system, with means comparisons by farming system.

**Table S3.** PERMANOVA pairwise comparisons of 16S rRNA gene ASV sequence profiles in samples grouped by different classification variables, based on Bray‐Curtis dissimilarity matrix.

**Table S4.** Taxa summary for bacterial taxa differentially represented at the family or genus levels comparing samples grouped by the classification variables.

**Table S5.** Taxa summary for fungi differentially represented at the family or genus level, comparing samples grouped by the classification variables.

**Table S6.** (A): OTU taxa in the shared microbiome of the three banana crops. (B): fungal shared microbiome of the three crops controls. (C): fungi in common between the shared microbiome of the banana plants and that of the adjacent controls. (D): Diversity indices of fungal OTUs per sample and farm management system. (E): Diversity indices of fungal OTUs by field management type, and plot by farming system.

**Table S7.** PERMANOVA pairwise comparisons of ITS1 sequence profiles in samples grouped by different classification variables, based on Bray‐Curtis dissimilarity matrix.

**Table S8.** Spearman’s correlation coefficients between ASV and OTUs in all samples.

**Table S9.** Spearman’s correlations and *p* values calculated among taxa (ASV and OTUs) in the banana (A) and control (B) samples. (C): Common correlated taxa found in banana and control samples.

## Data Availability

All 16S rRNA and ITS genes sequence datasets generated for this study were deposited at the National Center for Biotechnology Information (NCBI) Sequence Read Archive, under BioProject accession number PRJNA632470, available from: https://www.ncbi.nlm.nih.gov/sra.
