# Supplementary material for: Farming System and Nematodes Affect the Rhizosphere Microbiome of Tropical Banana Plants
Source: Environ Microbiol Rep. 2025 Jul 9;17(4):e70155. doi: 10.1111/1758-2229.70155 (PMC12241448; doi:10.1111/1758-2229.70155)

Figure S1. Venn diagram of the 16S ASV shared microbiomes of the banana plants and adjacent controls (A). Frequency of ASV in the banana rhizosphere samples compared with the controls, at the family level (B). ASV differentially represented among the three banana farming systems (C).

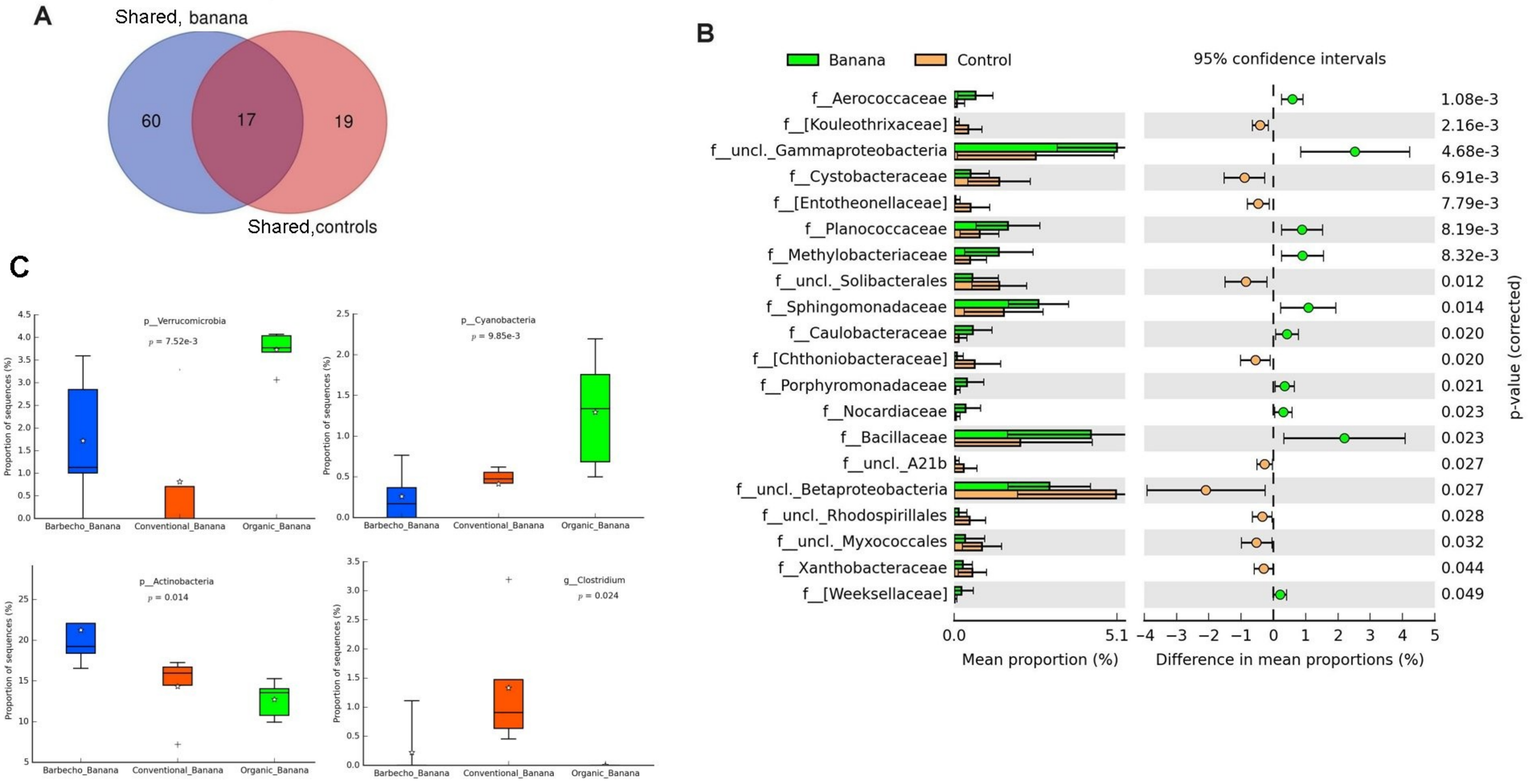

Supplement: Supplementary file 1 — Figure S1. (A): Venn diagram of the 16S rRNA gene ASV shared microbiomes of the banana plants and adjacent controls. (B): Frequency of ASV in the banana rhizosphere samples compared with the controls, at the family level. (C): ASV differentially represented in the three banana farming systems. [file EMI4-17-e70155-s001.pdf]
