# Supplementary material for: Farming System and Nematodes Affect the Rhizosphere Microbiome of Tropical Banana Plants
Source: Environ Microbiol Rep. 2025 Jul 9;17(4):e70155. doi: 10.1111/1758-2229.70155 (PMC12241448; doi:10.1111/1758-2229.70155)
Supplement: Supplementary file 2 — Figure S2. Frequency of top 10 bacterial taxa based on 16S rRNA gene ASV data comparing banana versus control, at the class (A) and family (B) level and, at the class level, for the different farming systems (C). Density of Xiphinema spp. and criconematids (D), Helicotylenchus multicinctus (E), and omnivorous/predatory nematodes (F). Effect of samples soil pH and P content on ASV (G). [file EMI4-17-e70155-s015.pdf]

Figure S2. Frequency of top ten bacterial taxa based on 16S ASV data, comparing banana *vs* control, at the class (A) and familiy (B) level and, at the class level, for the different crop sample types (C), the density of *Xiphinema* spp. and criconematids (D), *Helicotylenchus multicinctus* (E), and omnivorous/predatory nematodes (F). Effect of samples soil pH (AC = 5.5-5.5) and P content on ASV representations (G). The nematodes densities and soil P content were classified as medium (M, within a 10% confidence interval around all samples mean), lower (L) or higher (H) when below or above the 10% mean confidence interval, respectively.

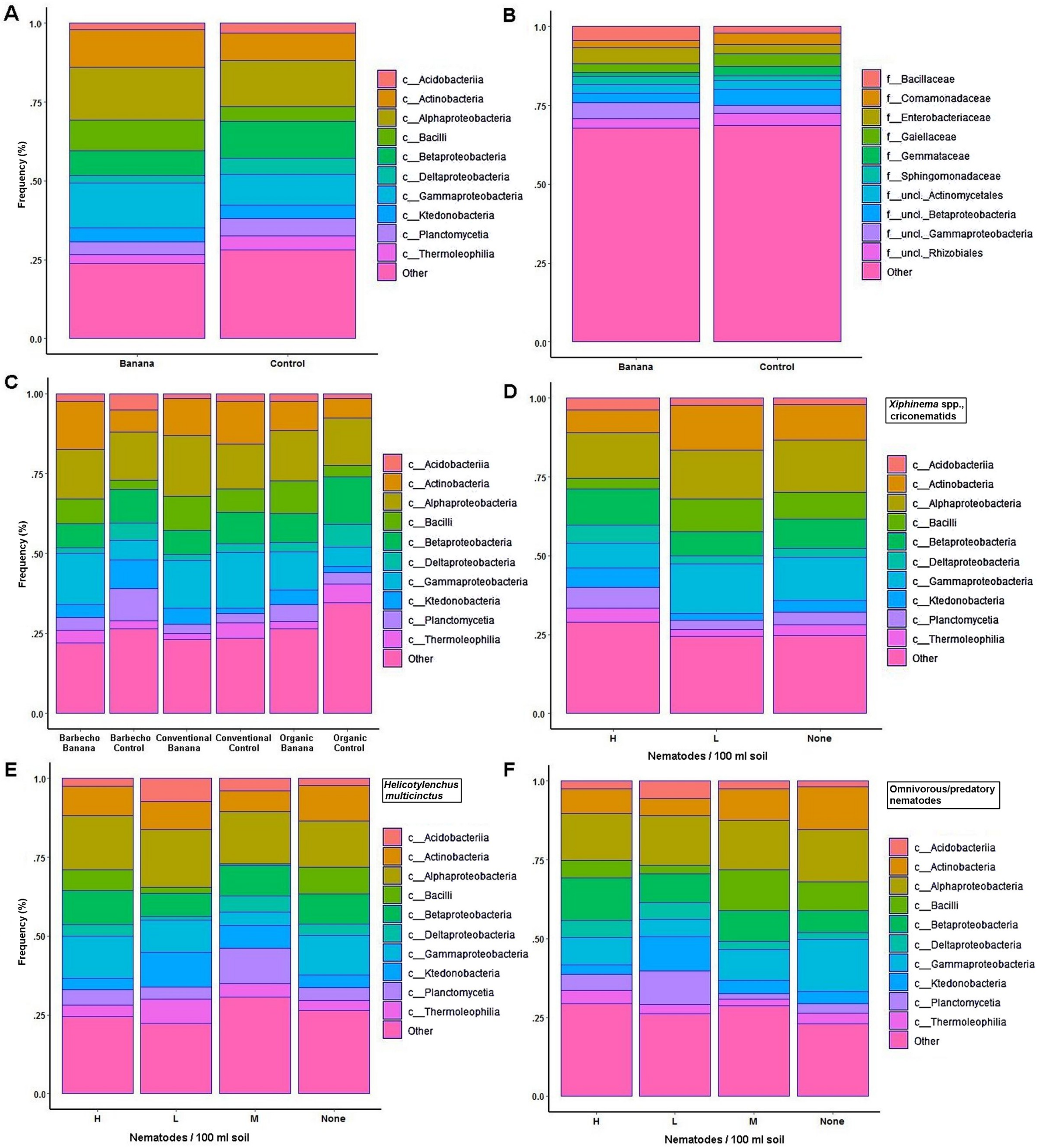

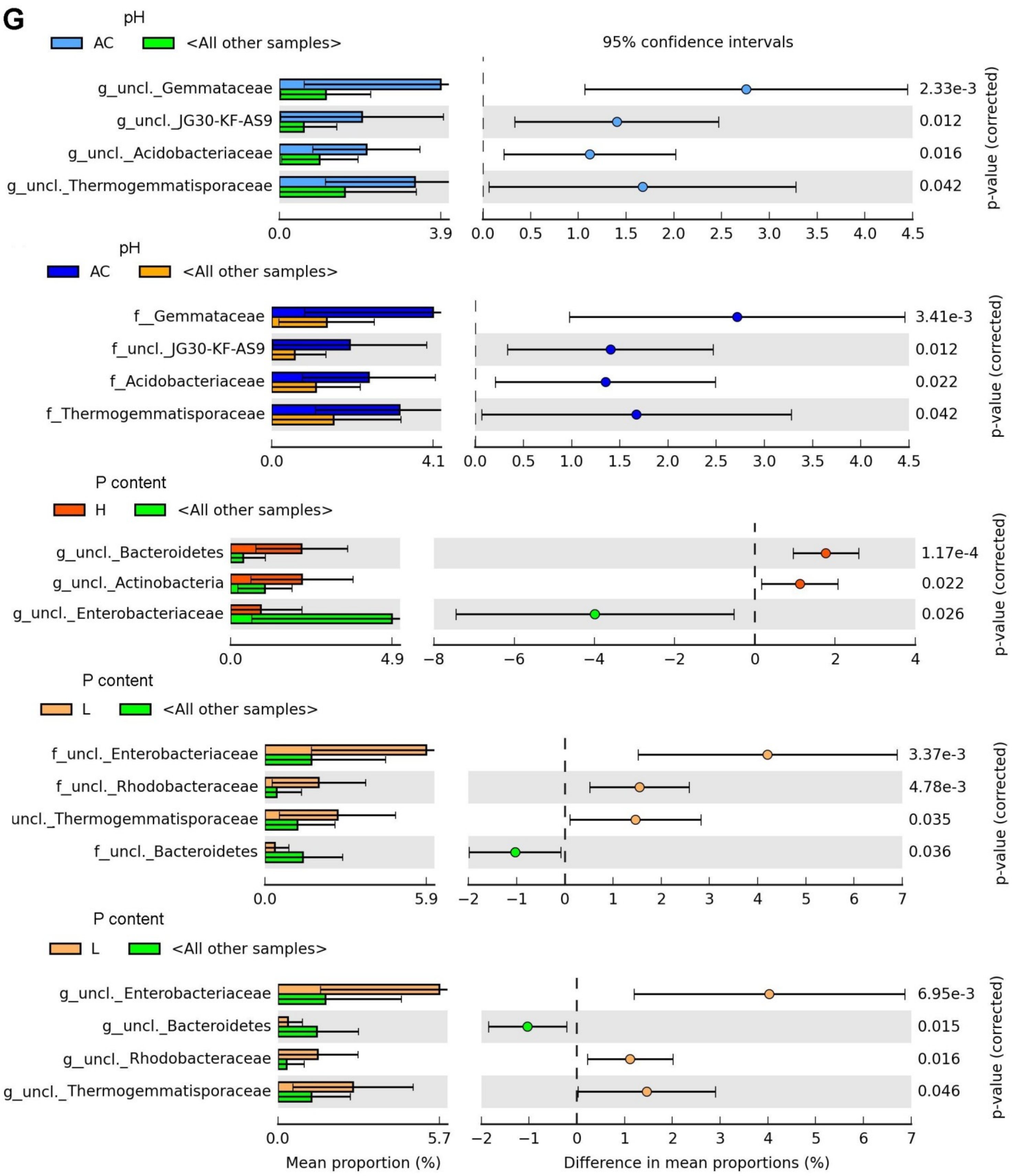
G
pH
AC
<All other samples>
95% confidence intervals
g_uncl._Gemmataceae
g_uncl._JG30-KF-AS9
g_uncl._Acidobacteriaceae
g_uncl._Thermogemmatisporaceae
2.33e-3
0.012
0.016
0.042
p-value (corrected)
pH
AC
<All other samples>
f__Gemmataceae
f_uncl._JG30-KF-AS9
f_Acidobacteriaceae
f_Thermogemmatisporaceae
3.41e-3
0.012
0.022
0.042
P content
H
<All other samples>
g_uncl._Bacteroidetes
g_uncl._Actinobacteria
g_uncl._Enterobacteriaceae
1.17e-4
0.022
0.026
P content
L
<All other samples>
f_uncl._Enterobacteriaceae
f_uncl._Rhodobacteraceae
uncl._Thermogemmatisporaceae
f_uncl._Bacteroidetes
3.37e-3
4.78e-3
0.035
0.036
P content
L
<All other samples>
g_uncl._Enterobacteriaceae
g_uncl._Bacteroidetes
g_uncl._Rhodobacteraceae
g_uncl._Thermogemmatisporaceae
6.95e-3
0.015
0.016
0.046
Mean proportion (%)
Difference in mean proportions (%)
