# Supplementary material for: Farming System and Nematodes Affect the Rhizosphere Microbiome of Tropical Banana Plants
Source: Environ Microbiol Rep. 2025 Jul 9;17(4):e70155. doi: 10.1111/1758-2229.70155 (PMC12241448; doi:10.1111/1758-2229.70155)

Figure S3. Frequency of 16S ASV in samples classified by nematode feeding group as plant parasitic (A, B), free living (C), other plant parasites (*Xiphinema*, criconematids, D) and omnivorous/predatory nematodes (E). NMDS plot showing the samples in relation to the crop type and levels of soil P content (F). The nematode or P soil levels were classified as medium (M, within a 10% confidence interval around all samples mean), lower (L) or higher (H) when below or above the 10% mean confidence interval, respectively.

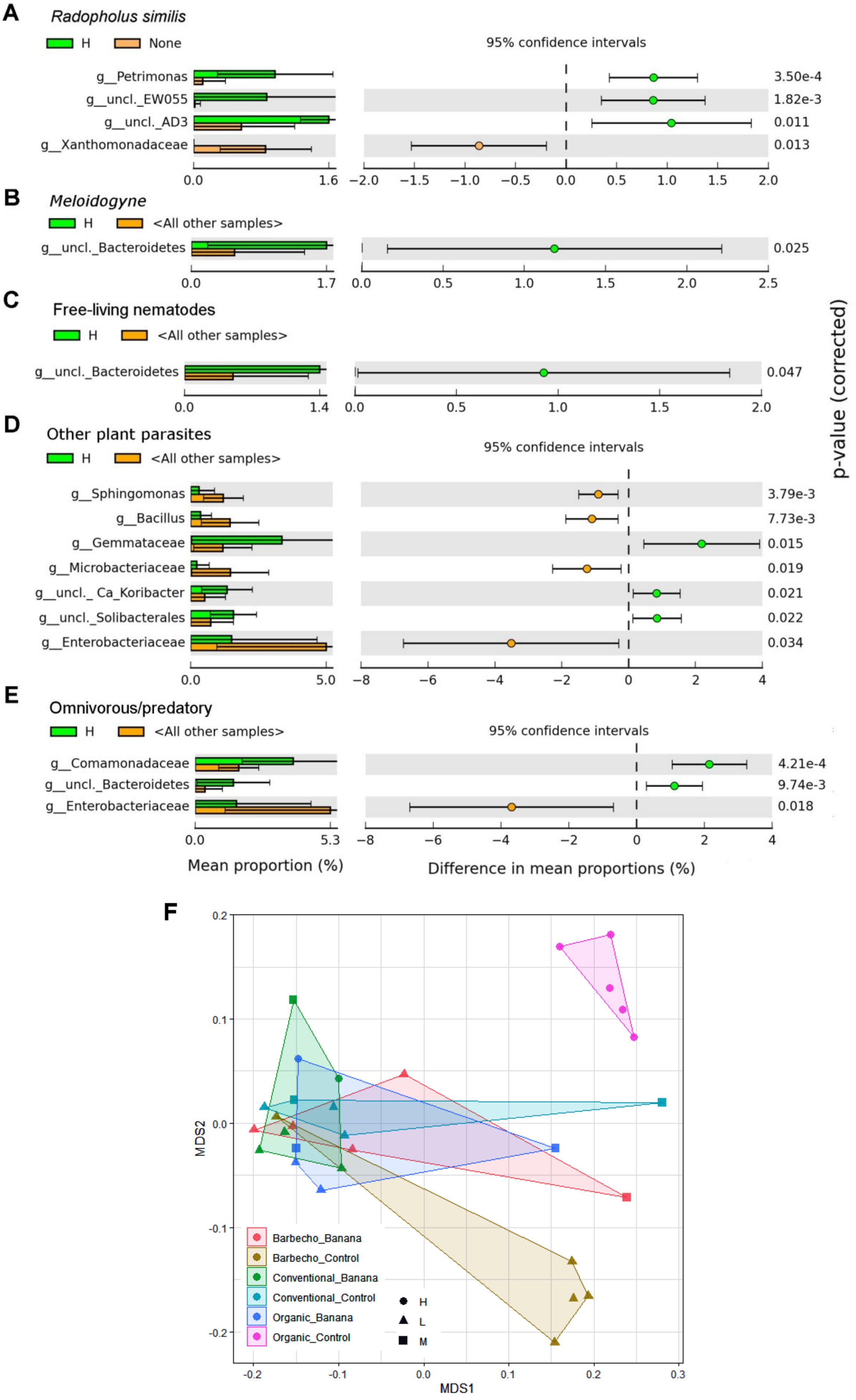

Supplement: Supplementary file 3 — Figure S3. Frequency of 16S rRNA gene ASV in samples classified by numbers of plant‐parasitic (A, B), free‐living (C), Xiphinema and criconematids (D) and omnivorous/predatory nematodes (E). NMDS plot of samples in relation to farming system and levels of soil P content (F). [file EMI4-17-e70155-s018.pdf]
