# Supplementary material for: Farming System and Nematodes Affect the Rhizosphere Microbiome of Tropical Banana Plants
Source: Environ Microbiol Rep. 2025 Jul 9;17(4):e70155. doi: 10.1111/1758-2229.70155 (PMC12241448; doi:10.1111/1758-2229.70155)

Figure S4. Abundance of ASV for genus *Cladosporium* in banana samples (A) and in organic banana *vs* organic control (B, C). Representation of uncl. members of Phaeotremellaceae, Symbiotaphrynaceae and Rozellomycota in organic banana (D). Frequency of ASV for uncl. members from order Capnodiales in organic (E) and conventional (F) banana samples compared to their controls, and of genus Archaeorhizomyces in the barbecho controls (G).

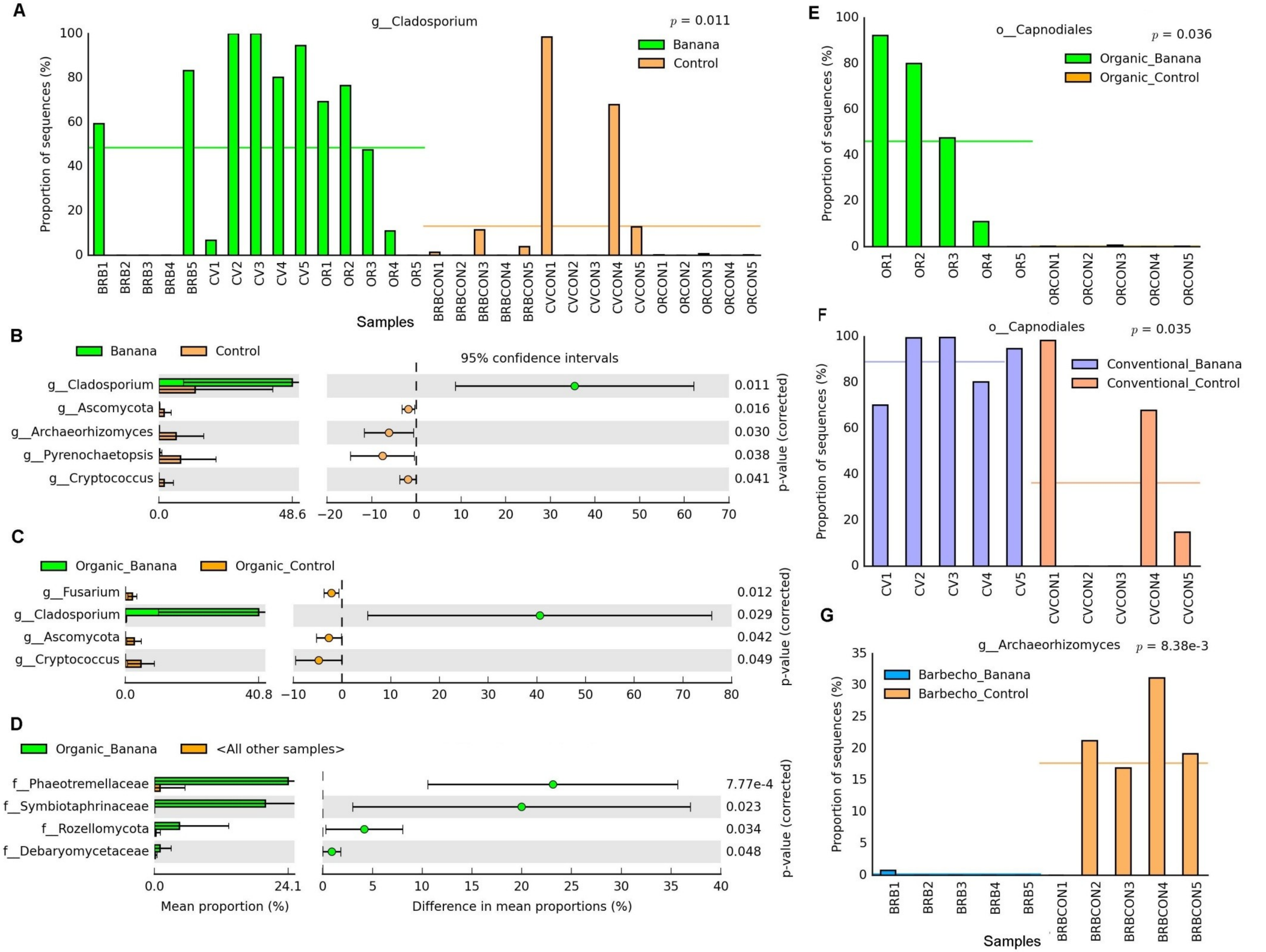

Supplement: Supplementary file 4 — Figure S4. Abundance of ASV for genus Cladosporium in banana samples (A) and in organic banana vs organic control (B, C). Abundance of unclassified Phaeotremellaceae, Symbiotaphrynaceae and Rozellomycota in organic banana (D). Frequency of ASV for unclassified members from Capnodiales in organic (E) and conventional (F) banana samples compared with controls, and of Archaeorhizomyces in the barbecho controls (G). [file EMI4-17-e70155-s014.pdf]
