# Supplementary material for: Farming System and Nematodes Affect the Rhizosphere Microbiome of Tropical Banana Plants
Source: Environ Microbiol Rep. 2025 Jul 9;17(4):e70155. doi: 10.1111/1758-2229.70155 (PMC12241448; doi:10.1111/1758-2229.70155)
Supplement: Supplementary file 5 — Figure S5. Frequency of fungal OTUs at different taxonomic levels, in samples classified by cropping (A) and farm management system (B, C). Comparison of organic banana crop versus organic control (E). [file EMI4-17-e70155-s006.pdf]

Figure S5. Frequency of fungal OTUs at different taxonomic levels, in samples classified by cropping (A) and by crop management types (B-C). Comparison of organic banana crop *vs* organic control (E)

A

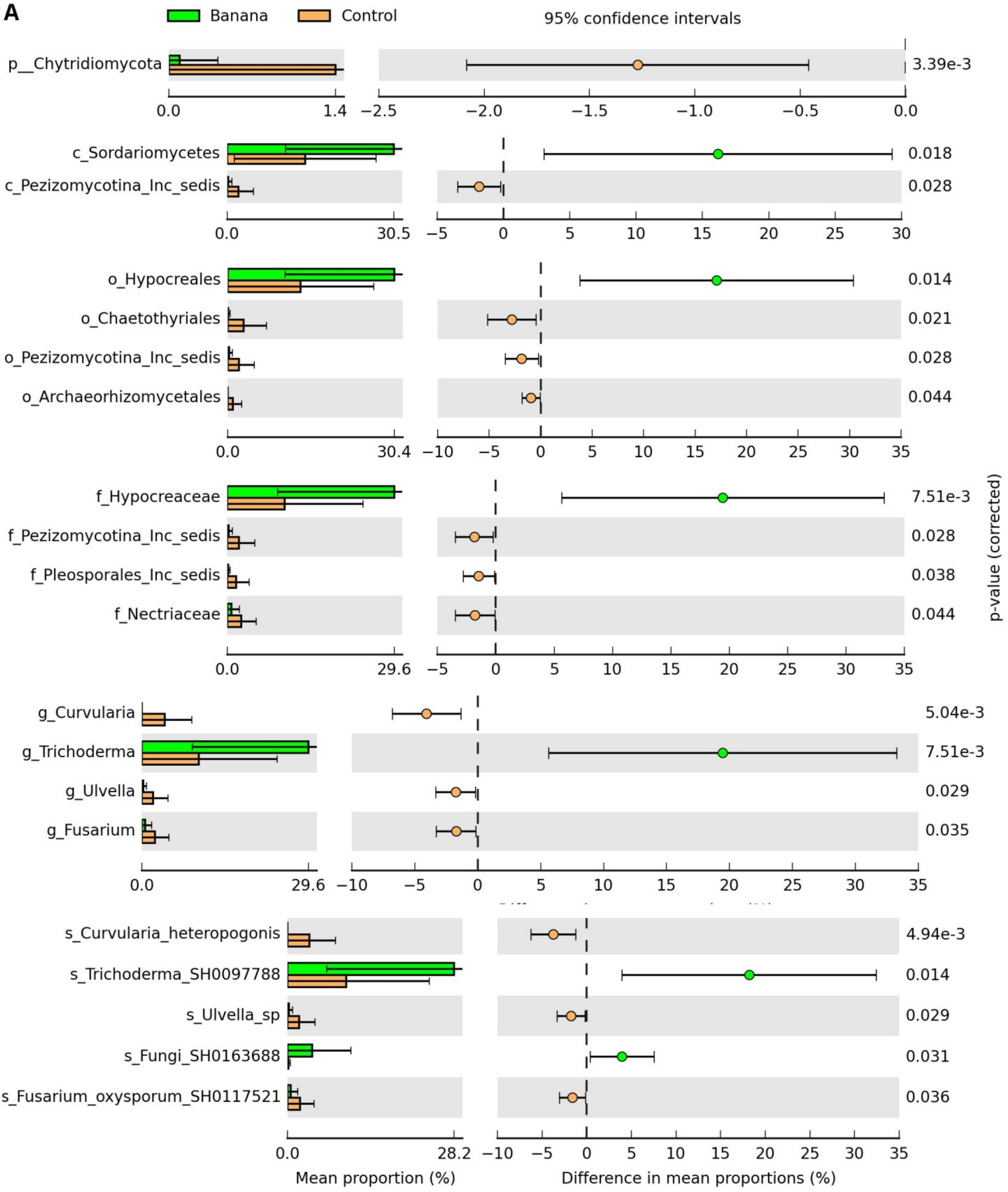

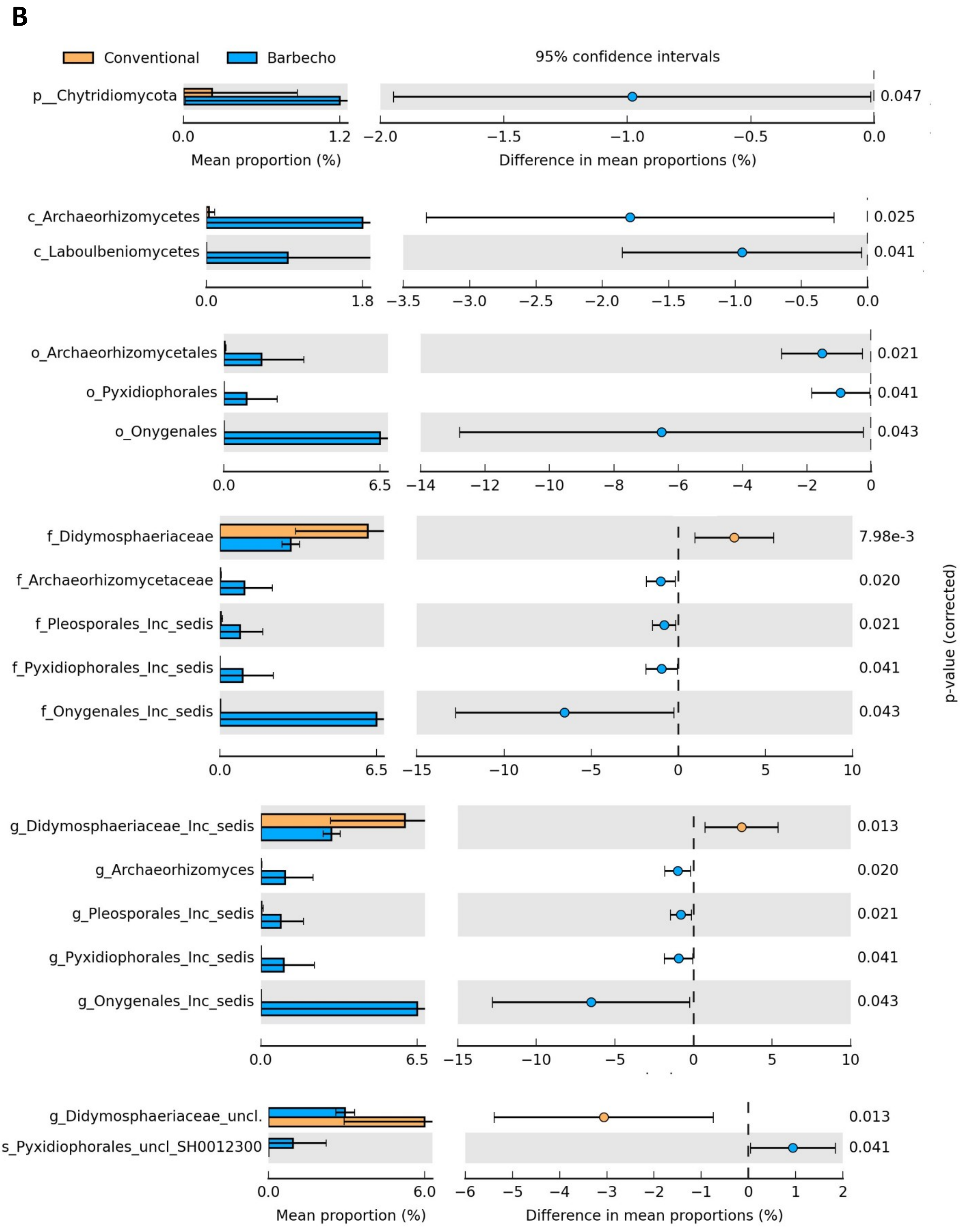
B
Conventional
Barbecho
95% confidence intervals
p__Chytridiomycota
0.047
Mean proportion (%)
Difference in mean proportions (%)
c_Archaeorhizomycetes
0.025
c_Laboulbeniomycetes
0.041
o_Archaeorhizomycetales
0.021
o_Pyxidiophorales
0.041
o_Onygenales
0.043
f_Didymosphaeriaceae
7.98e-3
f_Archaeorhizomycetaceae
0.020
f_Pleosporales_Inc_sedis
0.021
f_Pyxidiophorales_Inc_sedis
0.041
f_Onygenales_Inc_sedis
0.043
p-value (corrected)
g_Didymosphaeriaceae_Inc_sedis
0.013
g_Archaeorhizomyces
0.020
g_Pleosporales_Inc_sedis
0.021
g_Pyxidiophorales_Inc_sedis
0.041
g_Onygenales_Inc_sedis
0.043
g_Didymosphaeriaceae_uncl.
0.013
s_Pyxidiophorales_uncl_SH0012300
0.041
Mean proportion (%)
Difference in mean proportions (%)

C

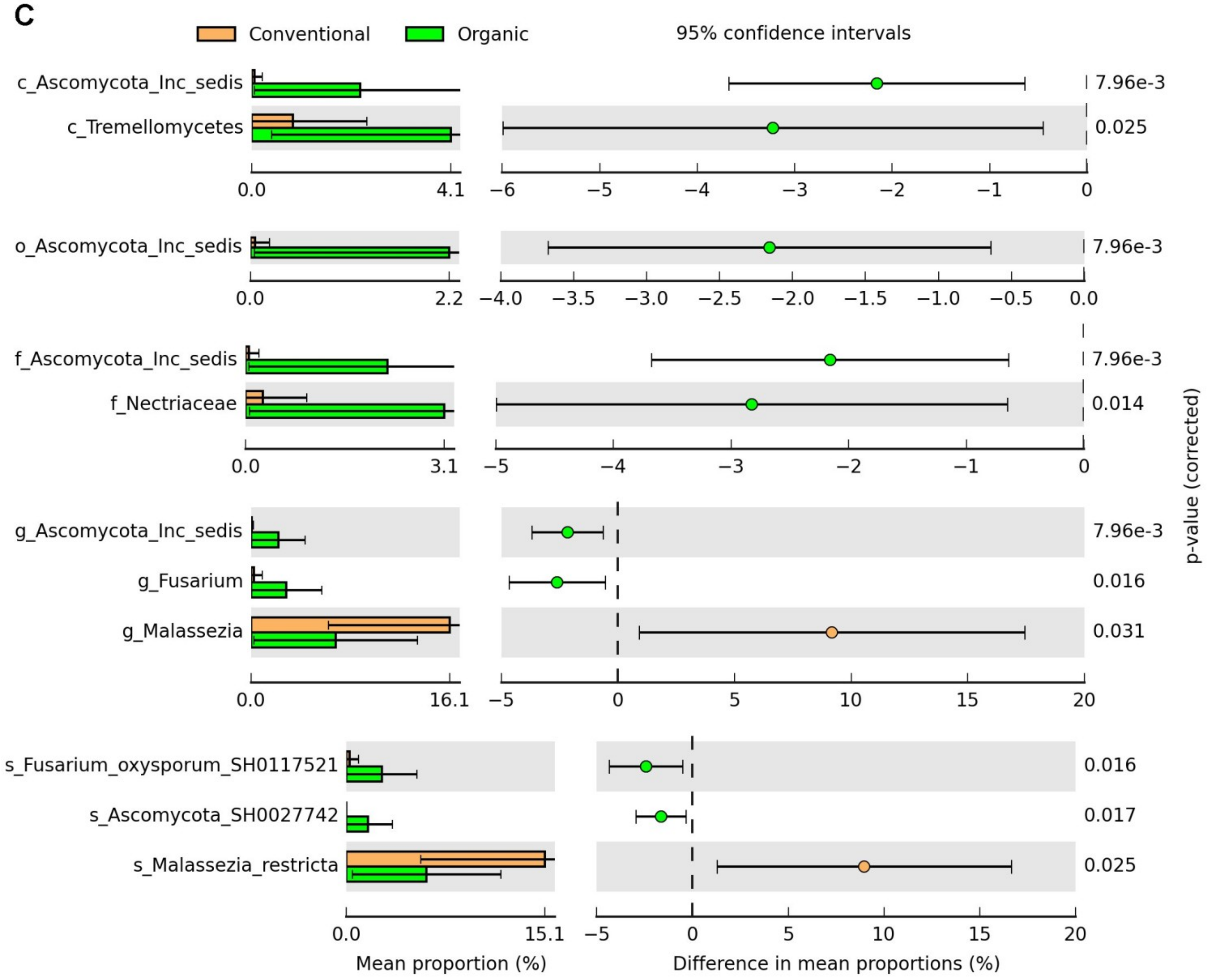

Conventional
Organic
95% confidence intervals
c_Ascomycota_Inc_sedis
c_Tremellomycetes
7.96e-3
0.025
0.0
4.1
−6
−5
−4
−3
−2
−1
0
o_Ascomycota_Inc_sedis
7.96e-3
0.0
2.2
−4.0
−3.5
−3.0
−2.5
−2.0
−1.5
−1.0
−0.5
0.0
f_Ascomycota_Inc_sedis
f_Nectriaceae
7.96e-3
0.014
0.0
3.1
−5
−4
−3
−2
−1
0
g_Ascomycota_Inc_sedis
g_Fusarium
g_Malassezia
7.96e-3
0.016
0.031
0.0
16.1
−5
0
5
10
15
20
s_Fusarium_oxysporum_SH0117521
s_Ascomycota_SH0027742
s_Malassezia_restricta
0.016
0.017
0.025
0.0
15.1
−5
0
5
10
15
20
Mean proportion (%)
Difference in mean proportions (%)
p-value (corrected)

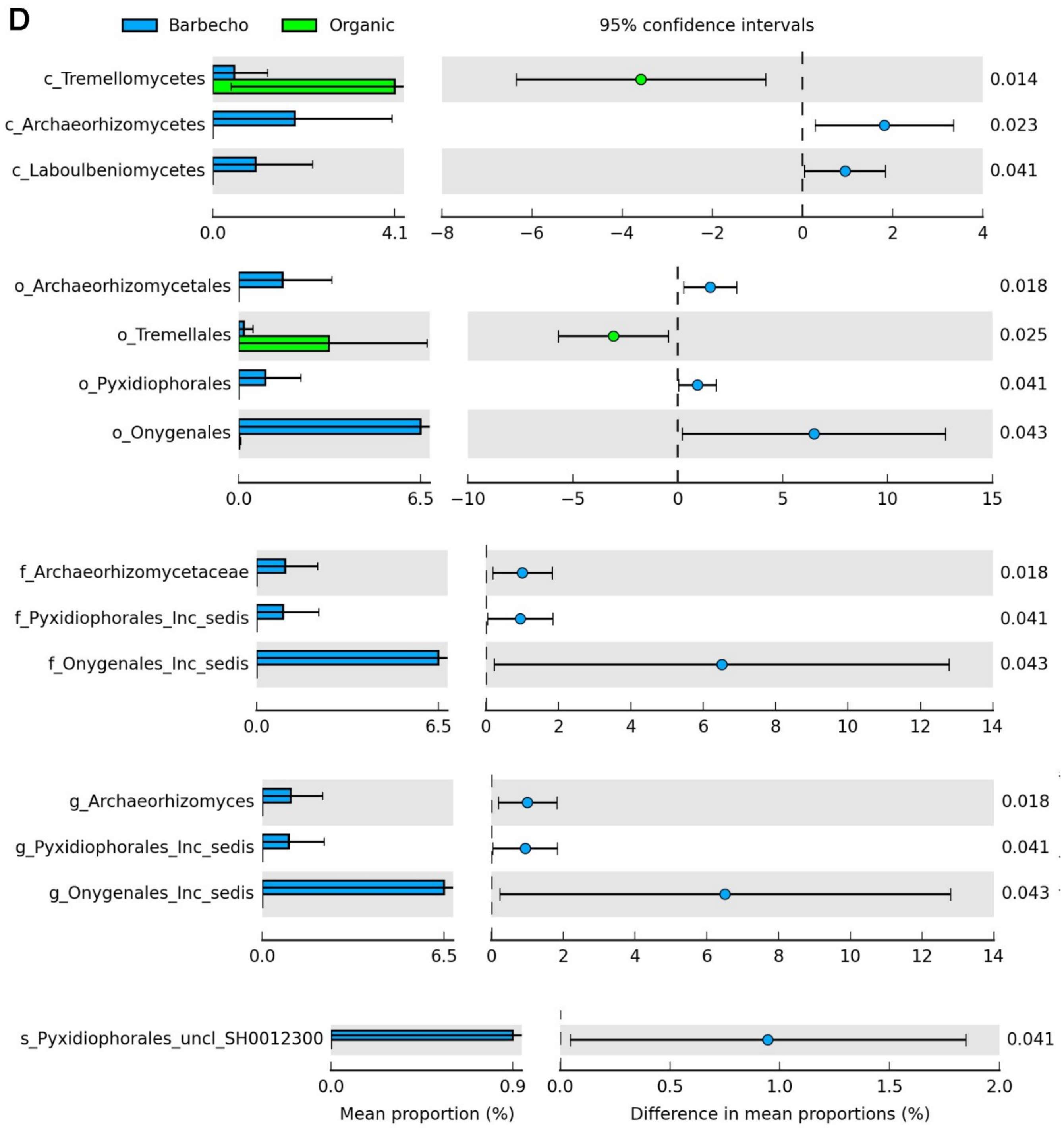
D
Barbecho
Organic
95% confidence intervals
c_Tremellomycetes
c_Archaeorhizomycetes
c_Laboulbeniomycetes
0.014
0.023
0.041
o_Archaeorhizomycetales
o_Tremellales
o_Pyxidiophorales
o_Onygenales
0.018
0.025
0.041
0.043
f_Archaeorhizomycetaceae
f_Pyxidiophorales_Inc_sedis
f_Onygenales_Inc_sedis
0.018
0.041
0.043
g_Archaeorhizomyces
g_Pyxidiophorales_Inc_sedis
g_Onygenales_Inc_sedis
0.018
0.041
0.043
s_Pyxidiophorales_uncl_SH0012300
0.041
p-value (corrected)
Mean proportion (%)
Difference in mean proportions (%)

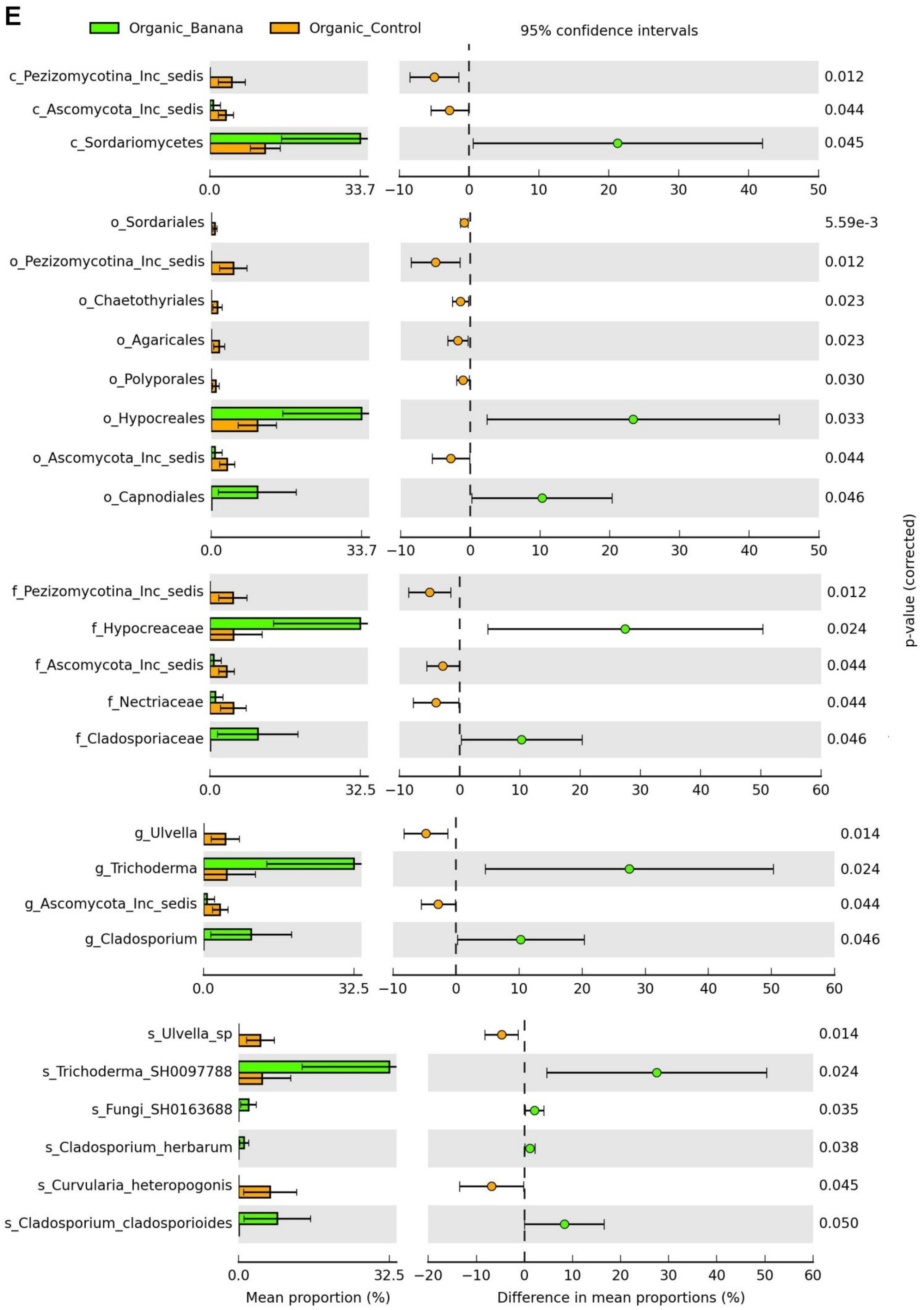

E
Organic_Banana
Organic_Control
95% confidence intervals
c_Pezizomycotina_Inc_sedis
c_Ascomycota_Inc_sedis
c_Sordariomycetes
0.012
0.044
0.045
o_Sordariales
o_Pezizomycotina_Inc_sedis
o_Chaetothyriales
o_Agaricales
o_Polyporales
o_Hypocreales
o_Ascomycota_Inc_sedis
o_Capnodiales
5.59e-3
0.012
0.023
0.023
0.030
0.033
0.044
0.046
f_Pezizomycotina_Inc_sedis
f_Hypocreaceae
f_Ascomycota_Inc_sedis
f_Nectriaceae
f_Cladosporiaceae
0.012
0.024
0.044
0.044
0.046
g_Ulvella
g_Trichoderma
g_Ascomycota_Inc_sedis
g_Cladosporium
0.014
0.024
0.044
0.046
s_Ulvella_sp
s_Trichoderma_SH0097788
s_Fungi_SH0163688
s_Cladosporium_herbarum
s_Curvularia_heteropogonis
s_Cladosporium_cladosporioides
0.014
0.024
0.035
0.038
0.045
0.050
0.0
33.7
32.5
−20
−10
0
10
20
30
40
50
60
Mean proportion (%)
Difference in mean proportions (%)
p-value (corrected)
