# Supplementary material for: Farming System and Nematodes Affect the Rhizosphere Microbiome of Tropical Banana Plants
Source: Environ Microbiol Rep. 2025 Jul 9;17(4):e70155. doi: 10.1111/1758-2229.70155 (PMC12241448; doi:10.1111/1758-2229.70155)
Supplement: Supplementary file 6 — Figure S6. Comparisons of 16S rRNA gene ASV profiles among controls: PCA plots for organic and barbecho controls (A). Comparisons of barbecho controls with the banana samples and the conventional control samples, at different taxonomic levels (B, C). Comparison of organic controls with conventional and barbecho controls (D, E). [file EMI4-17-e70155-s010.pdf]

Figure S6. Comparisons of ITS OTU profiles among control samples. PCA plots for the organic and barbecho control samples (A). Comparisons of the barbecho controls with the banana samples and with the conventional control samples, at different taxonomic levels (B, C). Comparison of organic controls with conventional and barbecho controls (D, E).

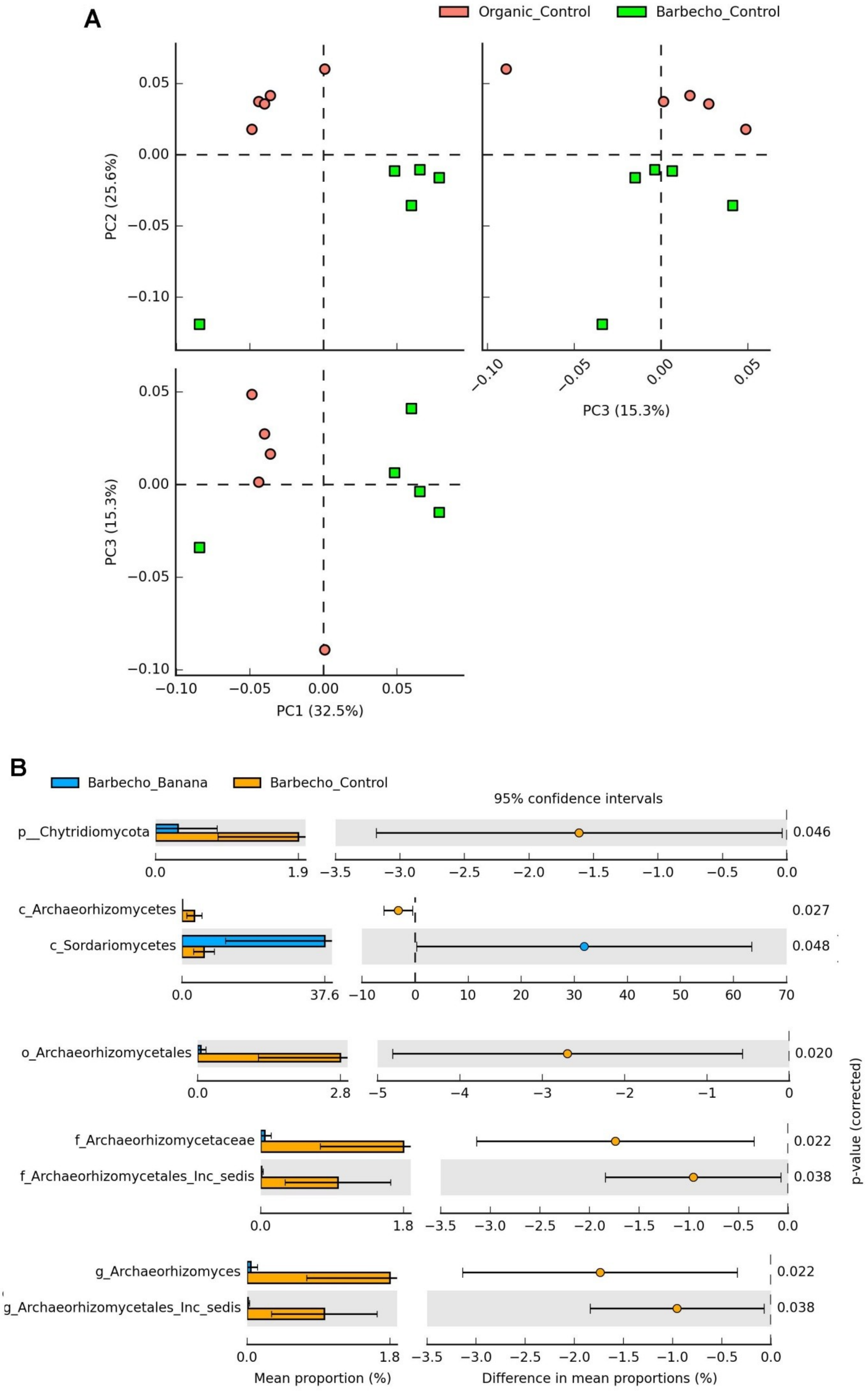

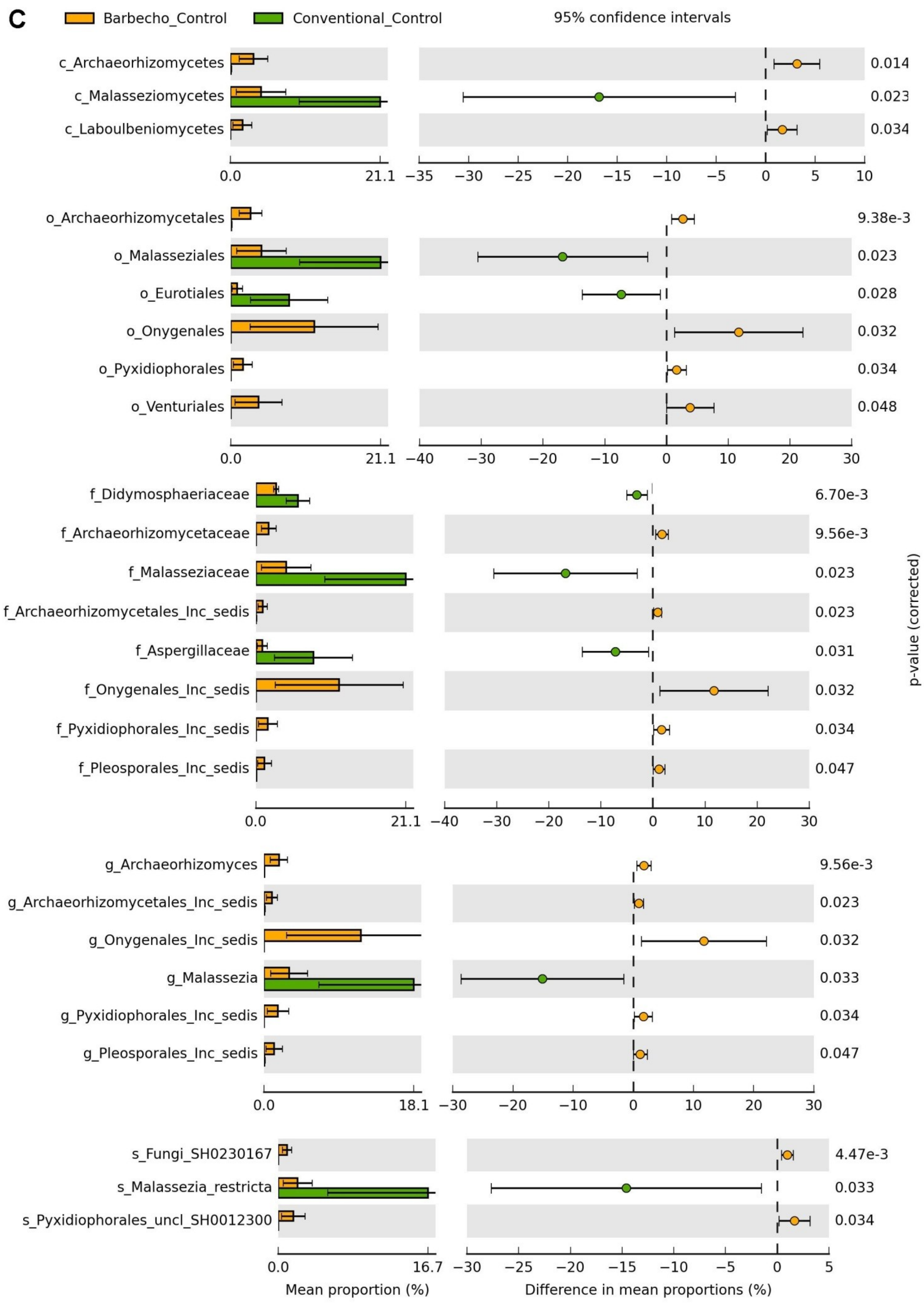

C
Barbecho_Control
Conventional_Control
95% confidence intervals
c_Archaeorhizomycetes
c_Malasseziomycetes
c_Laboulbeniomycetes
0.014
0.023
0.034
o_Archaeorhizomycetales
o_Malasseziales
o_Eurotiales
o_Onygenales
o_Pyxidiophorales
o_Venturiales
9.38e-3
0.023
0.028
0.032
0.034
0.048
f_Didymosphaeriaceae
f_Archaeorhizomycetaceae
f_Malasseziaceae
f_Archaeorhizomycetales_Inc_sedis
f_Aspergillaceae
f_Onygenales_Inc_sedis
f_Pyxidiophorales_Inc_sedis
f_Pleosporales_Inc_sedis
6.70e-3
9.56e-3
0.023
0.023
0.031
0.032
0.034
0.047
g_Archaeorhizomyces
g_Archaeorhizomycetales_Inc_sedis
g_Onygenales_Inc_sedis
g_Malassezia
g_Pyxidiophorales_Inc_sedis
g_Pleosporales_Inc_sedis
9.56e-3
0.023
0.032
0.033
0.034
0.047
s_Fungi_SH0230167
s_Malassezia_restricta
s_Pyxidiophorales_uncl_SH0012300
4.47e-3
0.033
0.034
p-value (corrected)
Mean proportion (%)
Difference in mean proportions (%)

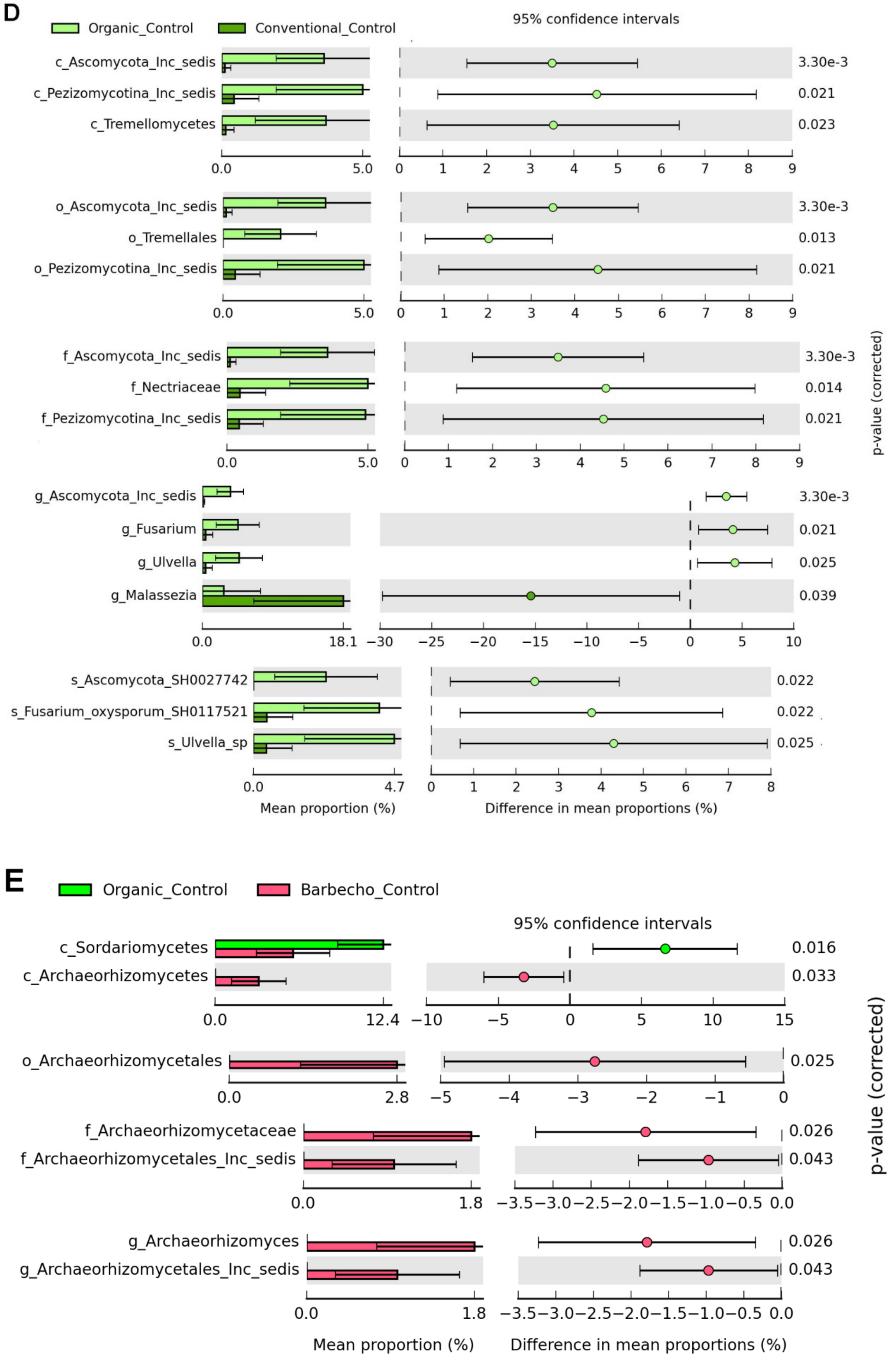

D
Organic_Control
Conventional_Control
95% confidence intervals
c_Ascomycota_Inc_sedis
c_Pezizomycotina_Inc_sedis
c_Tremellomycetes
3.30e-3
0.021
0.023
o_Ascomycota_Inc_sedis
o_Tremellales
o_Pezizomycotina_Inc_sedis
3.30e-3
0.013
0.021
f_Ascomycota_Inc_sedis
f_Nectriaceae
f_Pezizomycotina_Inc_sedis
3.30e-3
0.014
0.021
g_Ascomycota_Inc_sedis
g_Fusarium
g_Ulvella
g_Malassezia
3.30e-3
0.021
0.025
0.039
s_Ascomycota_SH0027742
s_Fusarium_oxysporum_SH0117521
s_Ulvella_sp
0.022
0.022
0.025
Mean proportion (%)
Difference in mean proportions (%)
p-value (corrected)
E
Organic_Control
Barbecho_Control
95% confidence intervals
c_Sordariomycetes
c_Archaeorhizomycetes
0.016
0.033
o_Archaeorhizomycetales
0.025
f_Archaeorhizomycetaceae
f_Archaeorhizomycetales_Inc_sedis
0.026
0.043
g_Archaeorhizomyces
g_Archaeorhizomycetales_Inc_sedis
0.026
0.043
Mean proportion (%)
Difference in mean proportions (%)
p-value (corrected)
