# Supplementary material for: Farming System and Nematodes Affect the Rhizosphere Microbiome of Tropical Banana Plants
Source: Environ Microbiol Rep. 2025 Jul 9;17(4):e70155. doi: 10.1111/1758-2229.70155 (PMC12241448; doi:10.1111/1758-2229.70155)
Supplement: Supplementary file 7 — Figure S7. Fungal taxa representation at different taxonomic levels, based on the density of Xiphinema spp. and criconematid nematodes (A), omnivorous and predatory nematodes (B), soil phosphorus content (C) and soil pH (D). [file EMI4-17-e70155-s012.pdf]

Figure S7. Fungal taxa representation at different taxonomic levels obtained by comparing samples classified by the density of *Xiphinema* spp. and criconematid nematodes (A), the density level of omnivorous and predatory nematodes (B), the levels of soil phosphorus content (C) and sample soil pH (D). For density classifications and codes see Supplementary Table S3.

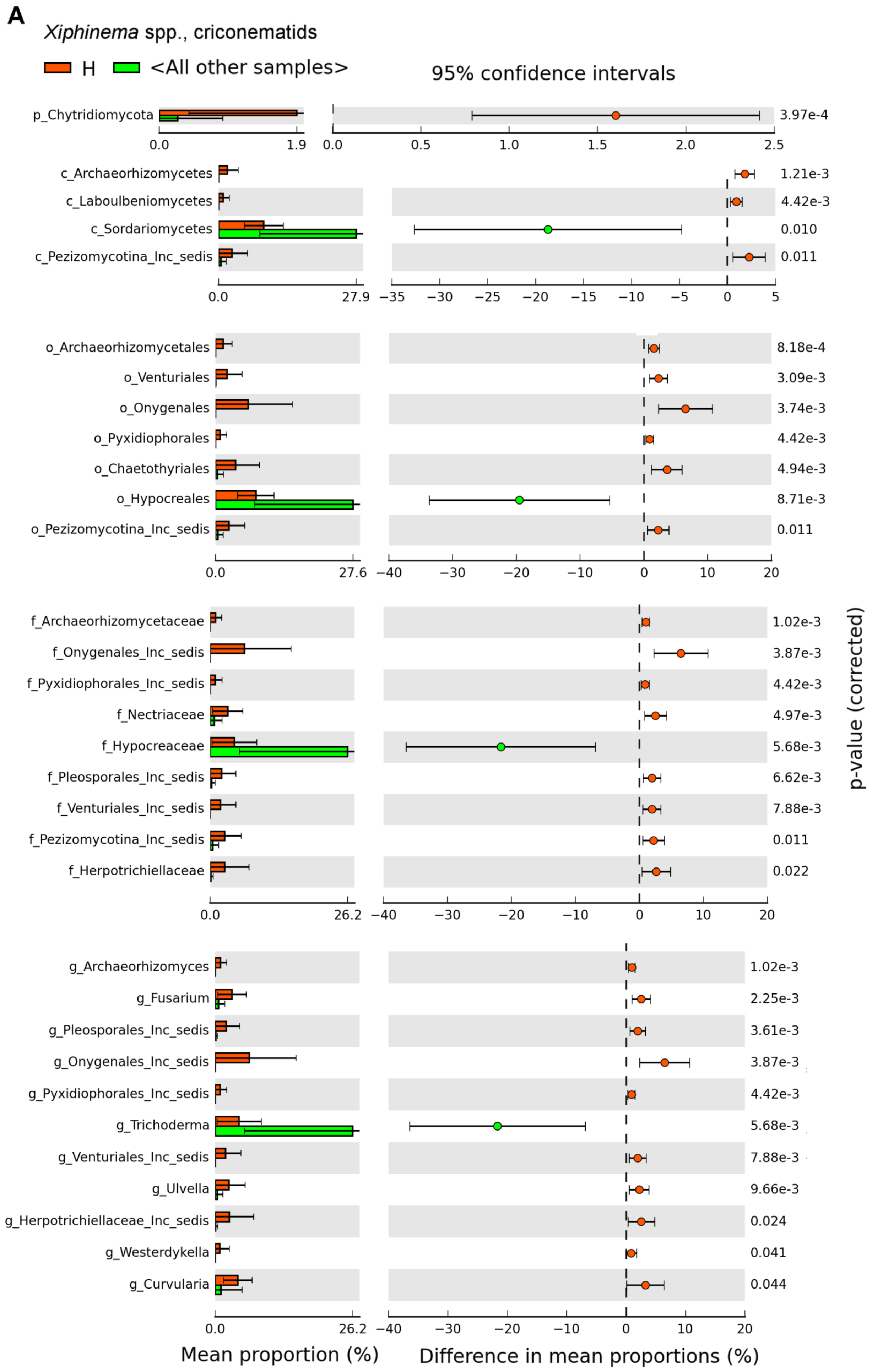

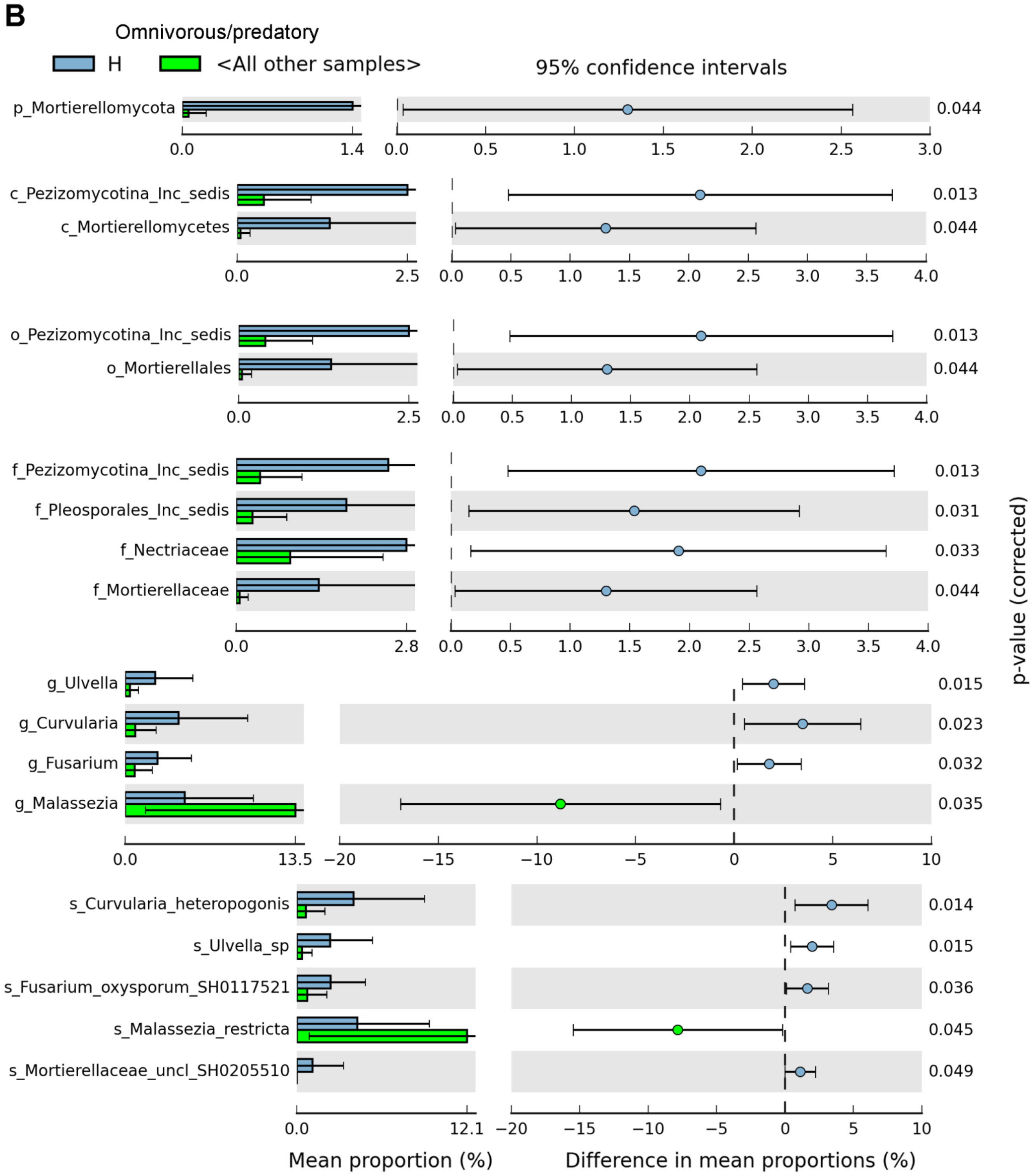

B
Omnivorous/predatory
H
<All other samples>
95% confidence intervals
p_Mortierellomycota
0.044
c_Pezizomycotina_Inc_sedis
0.013
c_Mortierellomycetes
0.044
o_Pezizomycotina_Inc_sedis
0.013
o_Mortierellales
0.044
f_Pezizomycotina_Inc_sedis
0.013
f_Pleosporales_Inc_sedis
0.031
f_Nectriaceae
0.033
f_Mortierellaceae
0.044
g_Ulvella
0.015
g_Curvularia
0.023
g_Fusarium
0.032
g_Malassezia
0.035
s_Curvularia_heteropogonis
0.014
s_Ulvella_sp
0.015
s_Fusarium_oxysporum_SH0117521
0.036
s_Malassezia_restricta
0.045
s_Mortierellaceae_uncl_SH0205510
0.049
p-value (corrected)
Mean proportion (%)
Difference in mean proportions (%)

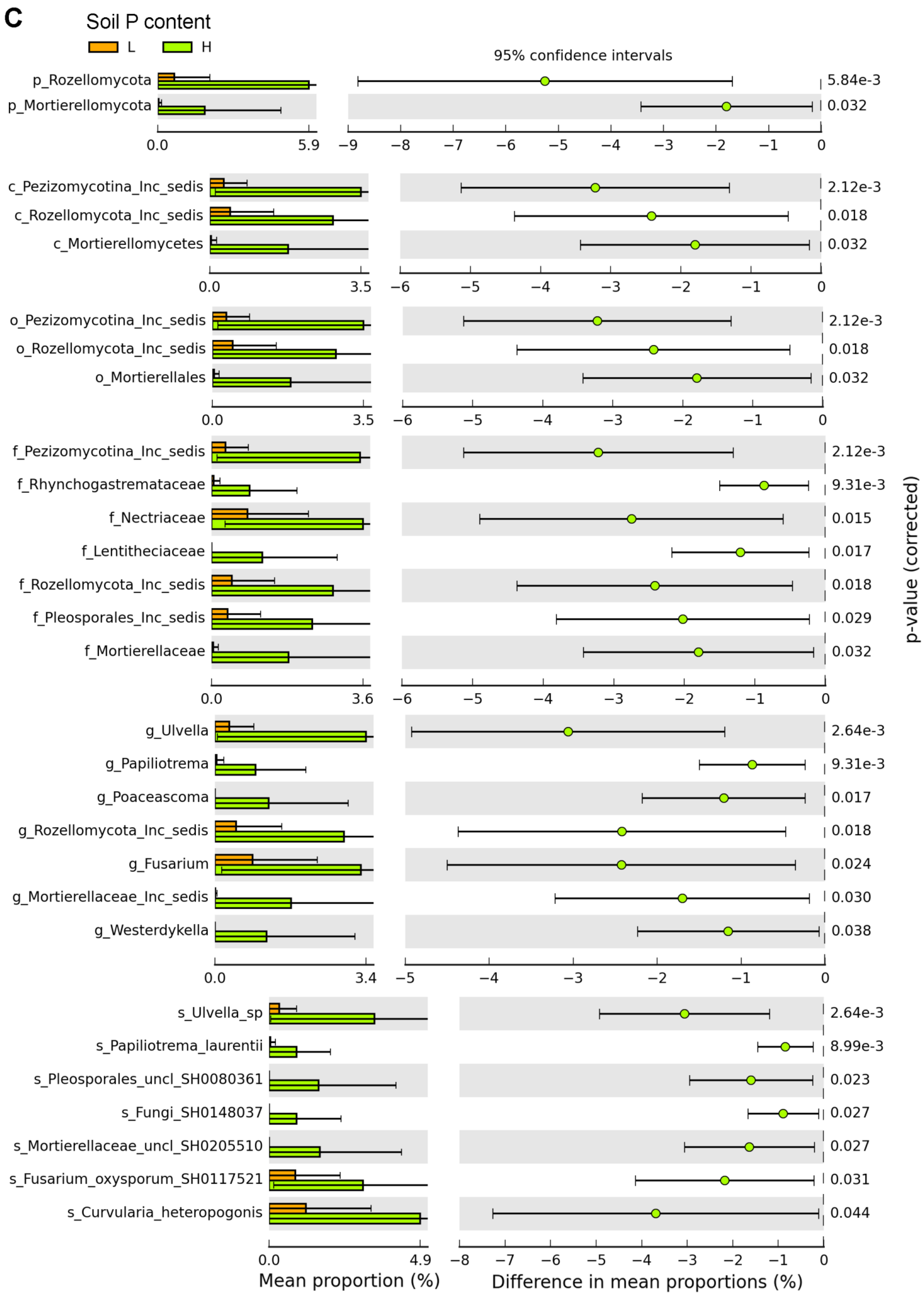
C
Soil P content
L
H
95% confidence intervals
p_Rozellomycota
p_Mortierellomycota
5.84e-3
0.032
c_Pezizomycotina_Inc_sedis
c_Rozellomycota_Inc_sedis
c_Mortierellomycetes
2.12e-3
0.018
0.032
o_Pezizomycotina_Inc_sedis
o_Rozellomycota_Inc_sedis
o_Mortierellales
2.12e-3
0.018
0.032
f_Pezizomycotina_Inc_sedis
f_Rhynchogastremataceae
f_Nectriaceae
f_Lentitheciaceae
f_Rozellomycota_Inc_sedis
f_Pleosporales_Inc_sedis
f_Mortierellaceae
2.12e-3
9.31e-3
0.015
0.017
0.018
0.029
0.032
g_Ulvella
g_Papiliotrema
g_Poaceascoma
g_Rozellomycota_Inc_sedis
g_Fusarium
g_Mortierellaceae_Inc_sedis
g_Westerdykella
2.64e-3
9.31e-3
0.017
0.018
0.024
0.030
0.038
s_Ulvella_sp
s_Papiliotrema_laurentii
s_Pleosporales_uncl_SH0080361
s_Fungi_SH0148037
s_Mortierellaceae_uncl_SH0205510
s_Fusarium_oxysporum_SH0117521
s_Curvularia_heteropogonis
2.64e-3
8.99e-3
0.023
0.027
0.027
0.031
0.044
p-value (corrected)
Mean proportion (%)
Difference in mean proportions (%)

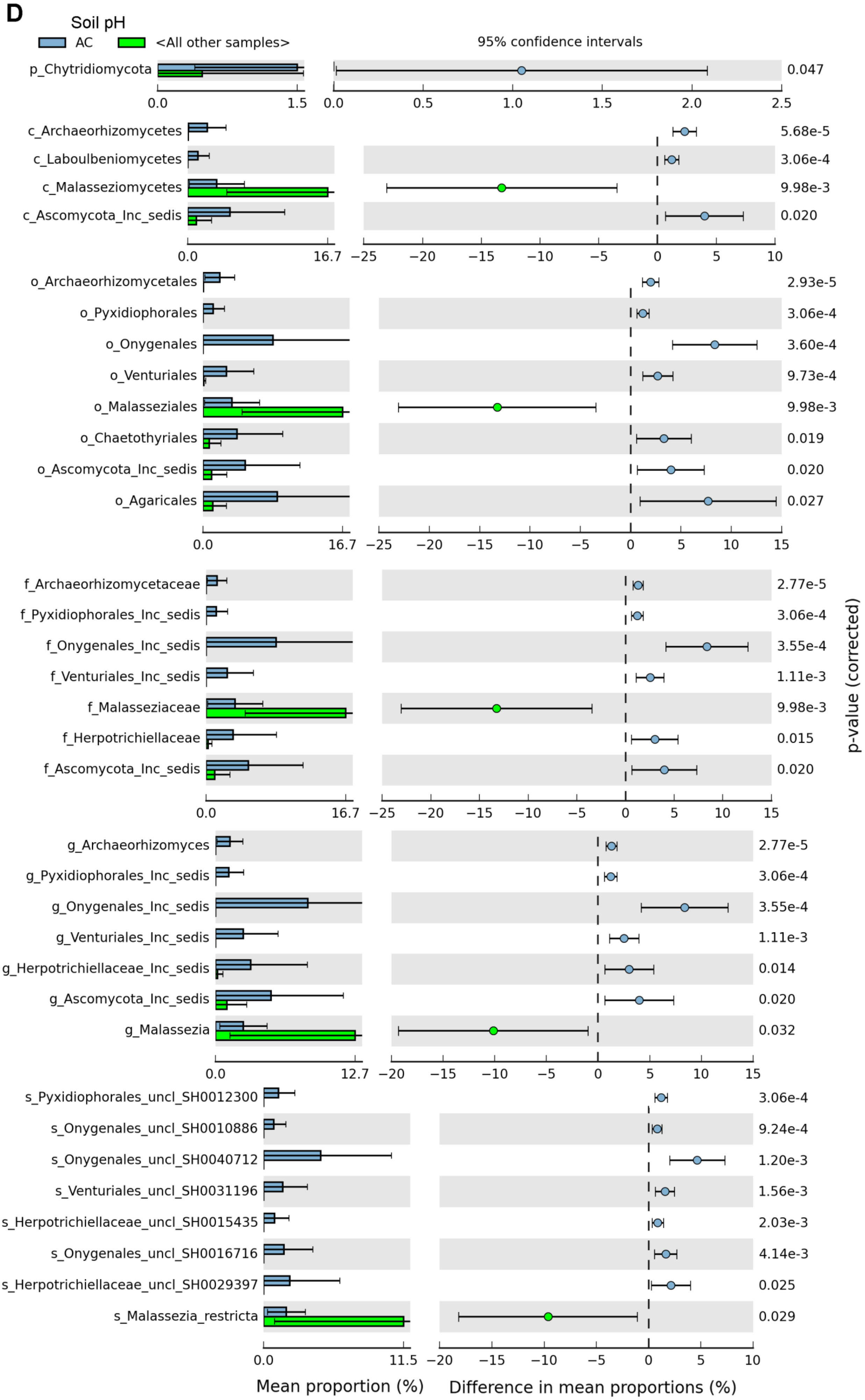

D
Soil pH
AC
<All other samples>
95% confidence intervals
p_Chytridiomycota
0.047
c_Archaeorhizomycetes
5.68e-5
c_Laboulbeniomycetes
3.06e-4
c_Malasseziomycetes
9.98e-3
c_Ascomycota_Inc_sedis
0.020
o_Archaeorhizomycetales
2.93e-5
o_Pyxidiophorales
3.06e-4
o_Onygenales
3.60e-4
o_Venturiales
9.73e-4
o_Malasseziales
9.98e-3
o_Chaetothyriales
0.019
o_Ascomycota_Inc_sedis
0.020
o_Agaricales
0.027
f_Archaeorhizomycetaceae
2.77e-5
f_Pyxidiophorales_Inc_sedis
3.06e-4
f_Onygenales_Inc_sedis
3.55e-4
f_Venturiales_Inc_sedis
1.11e-3
f_Malasseziaceae
9.98e-3
f_Herpotrichiellaceae
0.015
f_Ascomycota_Inc_sedis
0.020
g_Archaeorhizomyces
2.77e-5
g_Pyxidiophorales_Inc_sedis
3.06e-4
g_Onygenales_Inc_sedis
3.55e-4
g_Venturiales_Inc_sedis
1.11e-3
g_Herpotrichiellaceae_Inc_sedis
0.014
g_Ascomycota_Inc_sedis
0.020
g_Malassezia
0.032
s_Pyxidiophorales_uncl_SH0012300
3.06e-4
s_Onygenales_uncl_SH0010886
9.24e-4
s_Onygenales_uncl_SH0040712
1.20e-3
s_Venturiales_uncl_SH0031196
1.56e-3
s_Herpotrichiellaceae_uncl_SH0015435
2.03e-3
s_Onygenales_uncl_SH0016716
4.14e-3
s_Herpotrichiellaceae_uncl_SH0029397
0.025
s_Malassezia_restricta
0.029
Mean proportion (%)
Difference in mean proportions (%)
p-value (corrected)
