# Supplementary material for: Farming System and Nematodes Affect the Rhizosphere Microbiome of Tropical Banana Plants
Source: Environ Microbiol Rep. 2025 Jul 9;17(4):e70155. doi: 10.1111/1758-2229.70155 (PMC12241448; doi:10.1111/1758-2229.70155)

Figure S8. Fungal taxa representation obtained by comparing control samples of barbecho *vs* organic crops, at the class (A), order (B), family (C) and genus levels (D). OTUs representation obtained by comparing control samples of conventional crop with the barbecho controls at the family level (E) and, at the genus level, with the organic crop controls (F).

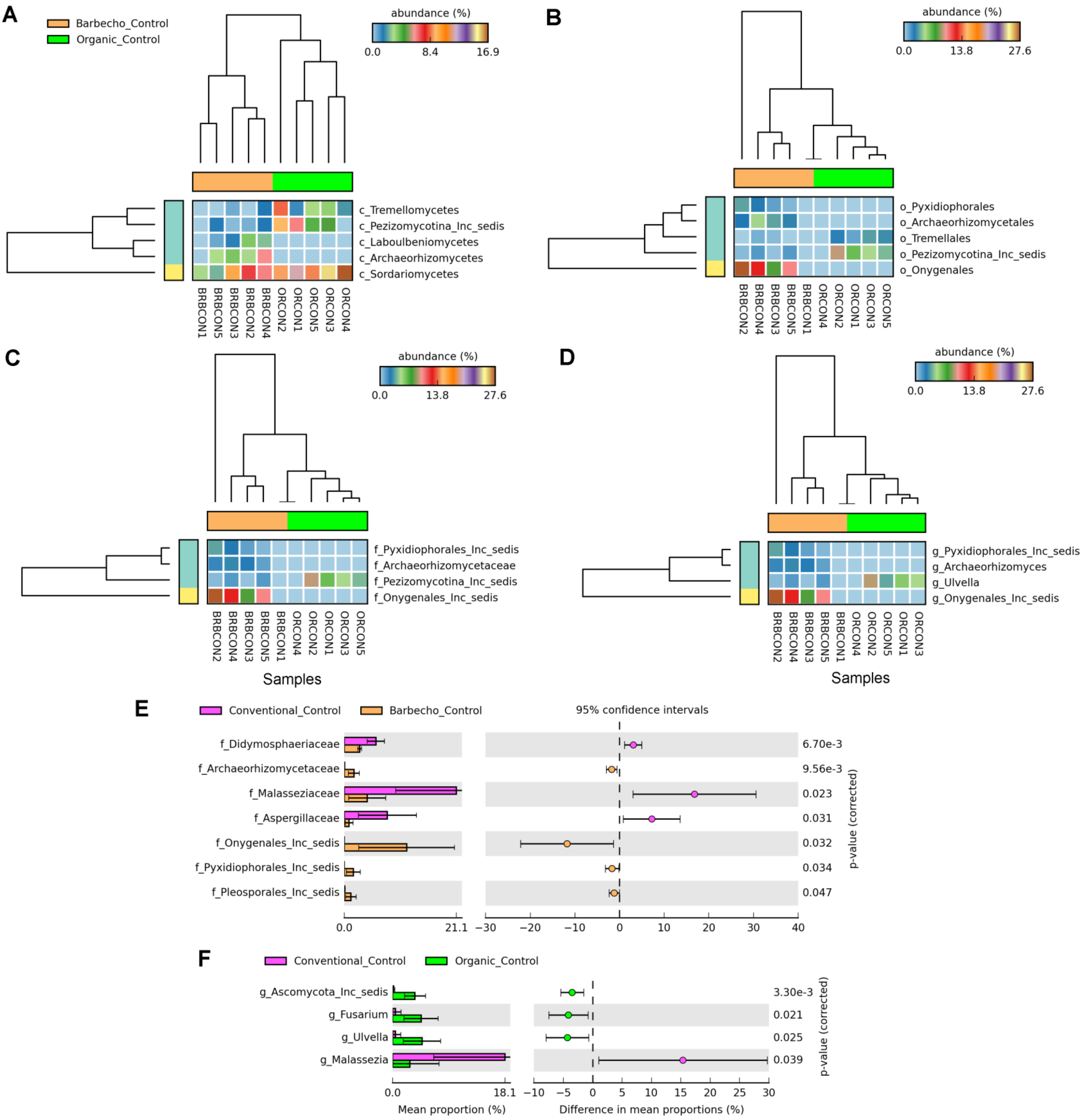

Supplement: Supplementary file 8 — Figure S8. Fungi representation comparing control of barbecho vs organic crops, at the class (A), order (B), family (C) and genus levels (D). OTUs representation by comparing conventional crop control with the barbecho (E) and the organic crop controls (F). [file EMI4-17-e70155-s017.pdf]
