# Supplementary material for: Farming System and Nematodes Affect the Rhizosphere Microbiome of Tropical Banana Plants
Source: Environ Microbiol Rep. 2025 Jul 9;17(4):e70155. doi: 10.1111/1758-2229.70155 (PMC12241448; doi:10.1111/1758-2229.70155)
Supplement: Supplementary file 9 — Figure S9. Spearman’s correlations (A) and correlations diagrams among ASVs and OTUs in the banana (B) and control samples (C). [file EMI4-17-e70155-s009.pdf]

Supplementary Figure S9. Spearman's correlations calculated among ASVs and OTUs present in more than 5 samples in the banana and control plants (A, $p < 0.05$). Correlations diagrams for the banana (B) and control samples (C). Vertical bars show coefficients p values.

A

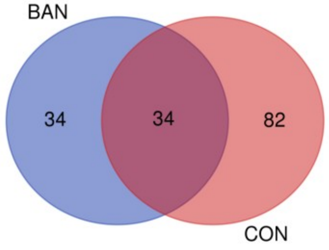

B

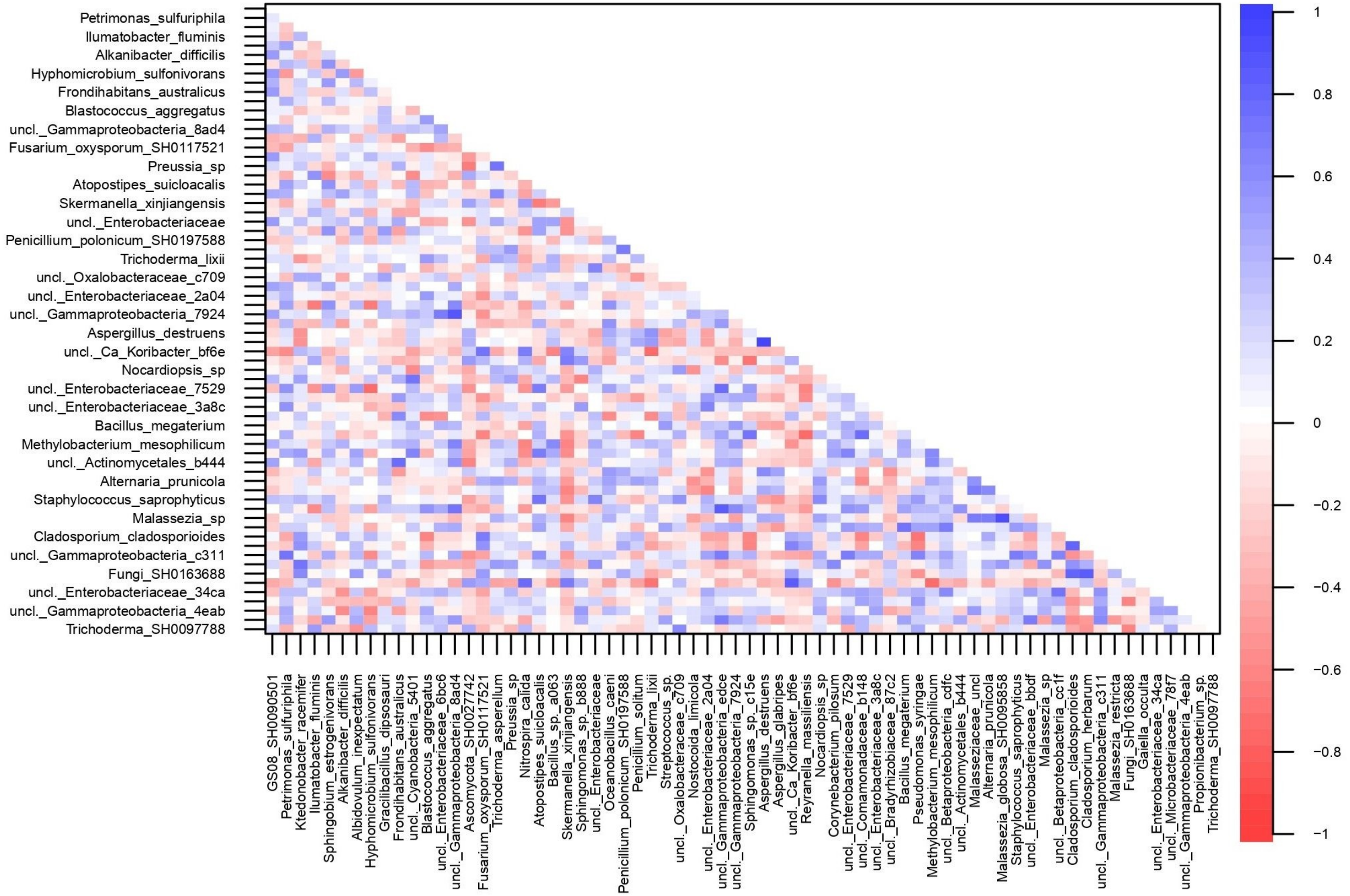

C

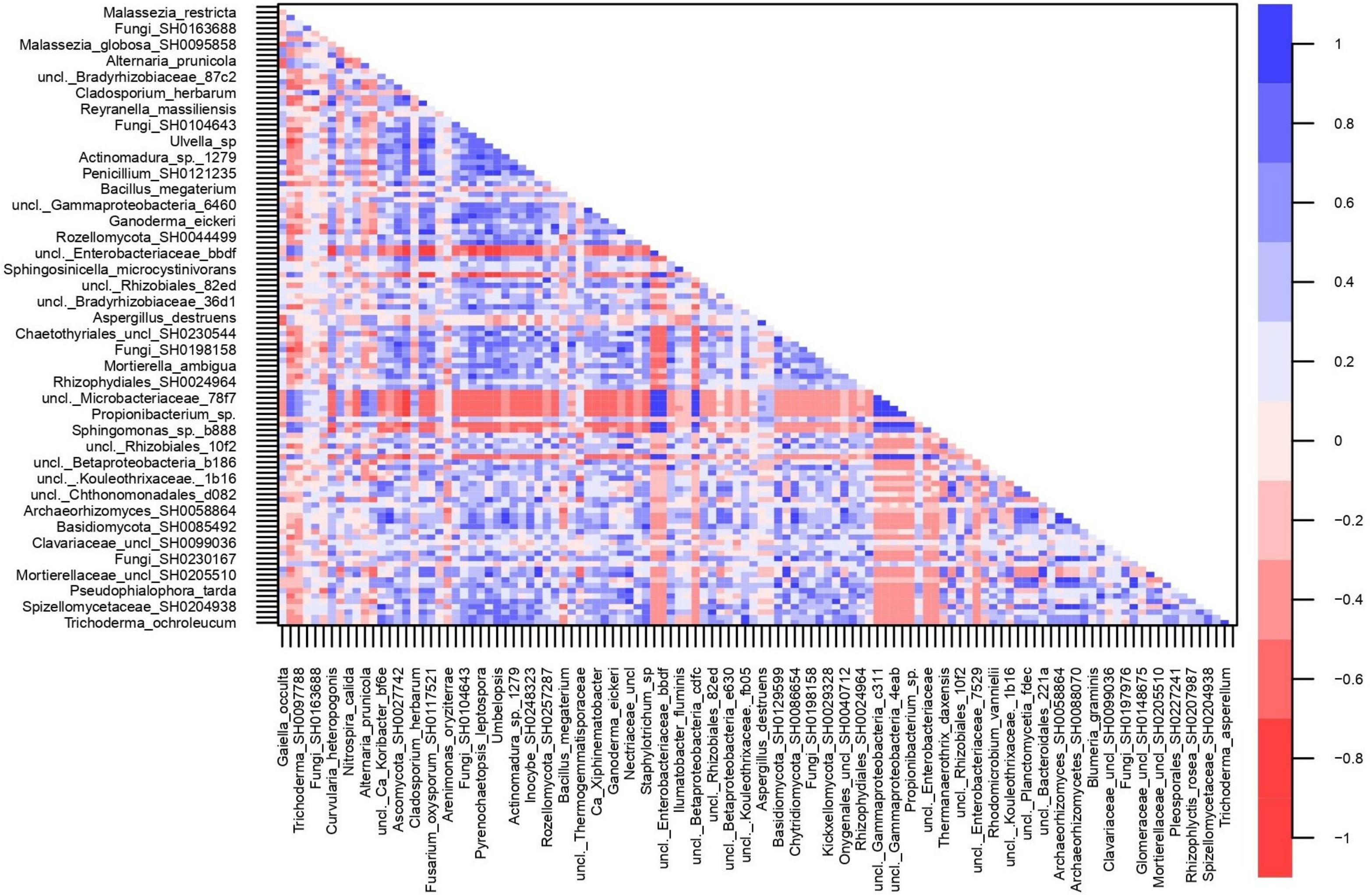
Malassezia_restricta
Fungi_SH0163688
Malassezia_globosa_SH0095858
Alternaria_prunicola
uncl._Bradyrhizobiaceae_87c2
Cladosporium_herbarum
Reyranella_massiliensis
Fungi_SH0104643
Ulvella_sp
Actinomadura_sp._1279
Penicillium_SH0121235
Bacillus_megaterium
uncl._Gammaproteobacteria_6460
Ganoderma_eickeri
Rozellomycota_SH0044499
uncl._Enterobacteriaceae_bbdf
Sphingosinicella_microcystinivorans
uncl._Rhizobiales_82ed
uncl._Bradyrhizobiaceae_36d1
Aspergillus_destruens
Chaetothyriales_uncl_SH0230544
Fungi_SH0198158
Mortierella_ambigua
Rhizophydiales_SH0024964
uncl._Microbacteriaceae_78f7
Propionibacterium_sp.
Sphingomonas_sp._b888
uncl._Rhizobiales_10f2
uncl._Betaproteobacteria_b186
uncl._.Kouleothrixaceae._1b16
uncl._Chthonomonadales_d082
Archaeorhizomyces_SH0058864
Basidiomycota_SH0085492
Clavariaceae_uncl_SH0099036
Fungi_SH0230167
Mortierellaceae_uncl_SH0205510
Pseudophialophora_tarda
Spizellomycetaceae_SH0204938
Trichoderma_ochroleucum
Gaiella_occulta
Trichoderma_SH0097788
Fungi_SH0163688
Curvularia_heteropogonis
Nitrospira_calida
Alternaria_prunicola
uncl._Ca_Koribacter_bf6e
Ascomycota_SH0027742
Cladosporium_herbarum
Fusarium_oxysporum_SH0117521
Arenimonas_oryziterrae
Fungi_SH0104643
Pyrenochaetopsis_leptospora
Umbelopsis
Actinomadura_sp._1279
Inocybe_SH0248323
Rozellomycota_SH0257287
Bacillus_megaterium
uncl._Thermogemmatisporaceae
Ca_Xiphinematobacter
Ganoderma_eickeri
Nectriaceae_uncl
Staphylotrichum_sp
uncl._Enterobacteriaceae_bbdf
Ilumatobacter_fluminis
uncl._Betaproteobacteria_cdfc
uncl._Rhizobiales_82ed
uncl._Betaproteobacteria_e630
uncl._Kouleothrixaceae._fb05
Aspergillus_destruens
Basidiomycota_SH0129599
Chytridiomycota_SH0086654
Fungi_SH0198158
Kickxellomycota_SH0029328
Onygenales_uncl_SH0040712
Rhizophydiales_SH0024964
uncl._Gammaproteobacteria_c311
uncl._Gammaproteobacteria_4eab
Propionibacterium_sp.
uncl._Enterobacteriaceae
Thermanaerothrix_daxensis
uncl._Rhizobiales_10f2
uncl._Enterobacteriaceae_7529
Rhodomicrobium_vannielii
uncl._Kouleothrixaceae._1b16
uncl._Planctomycetia_fdec
uncl._Bacteroidales_221a
Archaeorhizomyces_SH0058864
Archaeorhizomycetes_SH0088070
Blumeria_graminis
Clavariaceae_uncl_SH0099036
Fungi_SH0197976
Glomeraceae_uncl_SH0148675
Mortierellaceae_uncl_SH0205510
Pleosporales_SH0227241
Rhizophlyctis_rosea_SH0207987
Spizellomycetaceae_SH0204938
Trichoderma_asperellum
1
0.8
0.6
0.4
0.2
0
−0.2
−0.4
−0.6
−0.8
−1
