# Supplementary material for: Farming System and Nematodes Affect the Rhizosphere Microbiome of Tropical Banana Plants
Source: Environ Microbiol Rep. 2025 Jul 9;17(4):e70155. doi: 10.1111/1758-2229.70155 (PMC12241448; doi:10.1111/1758-2229.70155)
Supplement: Supplementary file 12 — Table S3. PERMANOVA pairwise comparisons of 16S rRNA gene ASV sequence profiles in samples grouped by different classification variables, based on Bray‐Curtis dissimilarity matrix. [file EMI4-17-e70155-s007.pdf]

Supplementary Table S3. Results of PERMANOVA pairwise comparisons of 16S sequence profiles in samples grouped by different classification variables (n. of samples = 30, based on Bray-Curtis dissimilarity matrix).

Var 1	Var 2	R^2	P [a]	P Bonferroni [a]	P FDR [a]
Crop					
Banana	Control	0.0571824	**0.007**	**0.007**	**0.007**
Management					
Barbecho	Conventional	0.07066627	**0.047**	0.141	0.071
Barbecho	Organic	0.06628508	0.057	0.171	0.057
Conventional	Organic	0.07528390	**0.019**	0.057	0.057
Description					
Barbecho Banana	Barbecho Control	0.1571563	0.083	1.245	0.138
Barbecho Banana	Conventional Banana	0.1045935	0.775	11.625	0.775
Barbecho Banana	Conventional Control	0.1065037	0.598	8.970	0.690
Barbecho Banana	Organic Banana	0.1074785	0.579	8.685	0.724
Barbecho Banana	Organic Control	0.1747198	**0.018**	0.270	0.067
Barbecho Control	Conventional Banana	0.1860737	**0.043**	0.645	0.092
Barbecho Control	Conventional Control	0.1691518	**0.028**	0.420	0.070
Barbecho Control	Organic Banana	0.1641659	**0.049**	0.735	0.092
Barbecho Control	Organic Control	0.1928616	**0.008**	0.120	**0.048**
Conventional Banana	Conventional Control	0.1110201	0.445	6.675	0.607
Conventional Banana	Organic Banana	0.1060280	0.747	11.205	0.800
Conventional Banana	Organic Control	0.2126020	**0.008**	0.120	**0.048**
Conventional Control	Organic Banana	0.1137160	0.379	5.685	0.569
Conventional Control	Organic Control	0.1864970	**0.019**	0.285	0.057
Organic Banana	Organic Control	0.1892929	**0.007**	0.105	0.105
Meloidogyne sp.[b]					
H	L	0.12892323	0.810	4.860	0.972
H	M	0.13086665	0.095	0.570	0.570
H	None	0.04016381	0.680	4.080	1.020
L	M	0.20749067	0.333	2.000	0.667
L	None	0.04256104	0.972	5.832	0.972
M	None	0.05221598	0.188	1.128	0.564

Omnivorous/predatory nematodes [c]					
H	L	0.09095372	**0.060**	0.360	0.120
H	M	0.09173079	0.493	2.958	0.493
H	None	0.07259395	**0.004**	**0.024**	**0.016**
L	M	0.28668793	0.400	2.400	0.480
L	None	0.10637000	**0.004**	**0.024**	**0.016**
M	None	0.07395923	0.361	2.166	0.541
Helicotylenchus spp. [d]					
H	L	0.12240467	0.294	1.764	0.441
H	M	0.10628417	0.251	1.506	0.502
H	None	0.03233493	0.971	5.826	1.165
L	M	0.47271425	1.000	6.000	1.000
L	None	0.06313696	**0.045**	0.270	0.270
M	None	0.05875460	0.162	0.972	0.486
Radopholus similis [e]					
H	None	0.0325523	0.565	0.565	0.565
Other plant parasitic nem.[f]					
H	L	0.12617041	0.149	0.447	0.223
H	None	0.06177138	**0.006**	**0.018**	**0.018**
L	None	0.04575820	0.885	2.655	0.885
Free-living nematodes [g]					
H	L	0.03863168	0.357	1.071	0.357
H	None	0.09351773	0.278	0.834	0.834
L	None	0.05025651	0.332	0.996	0.498
pH [*]					
AC	MAC	0.12341715	0.363	2.178	0.726
AC	N	0.05500107	**0.043**	0.258	0.258
AC	SAC	0.10788640	0.258	1.548	0.774
MAC	N	0.05035807	0.865	5.190	1.297
MAC	SAC	0.23186347	0.900	5.400	1.080
N	SAC	0.04450599	0.951	5.706	0.951

Phosphorous[**]					
H	L	0.07981426	**0.002**	**0.006**	**0.006**
H	M	0.09439173	0.124	0.372	0.124
L	M	0.05420458	0.103	0.309	0.154

[a] Significant values (P < 0.05) are shown in bold.
[b] Nematodes / 100 ml soil. L = low density < 90% mean; M = medium density within mean ± 10%; H = high > 110% mean. All samples mean ± SD = 18 ± 46
[c] Nematodes / 100 ml soil. L = low density < 90% mean; M = medium density within mean ± 10%; H = high > 110% mean. All samples mean ± SD = 20 ± 27
[d] Nematodes / 100 ml soil. L = low density < 90% mean; M = medium density within mean ± 10%; H = high > 110% mean. All samples mean ± SD = 11 ± 17
[e] Nematodes / 100 ml soil. L = low density < 90% mean; M = medium density within mean ± 10%; H = high > 110% mean. All samples mean ± SD = 4 ± 16
[f] Nematodes / 100 ml soil. L = low density < 90% mean; M = medium density within mean ± 10%; H = high > 110% mean. All samples mean ± SD = 7 ± 17
[g] Nematodes / 100 ml soil. L = low density < 90% mean; M = medium density within mean ± 10%; H = high > 110% mean. All samples mean ± SD = 172 ± 212
[*] AC = acidic, 5.0-5.5; M = moderately acidic, 5.5-6.0; SAC = slightly acidic, 6.0-6.5; N = neutral, 6.5-7.5.
[**] L = low, ≤ 22.9 ppm ; M = mean, 23.0 - 34.9 ppm; H = high, ≥ 35 ppm.
