# Supplementary material for: Farming System and Nematodes Affect the Rhizosphere Microbiome of Tropical Banana Plants
Source: Environ Microbiol Rep. 2025 Jul 9;17(4):e70155. doi: 10.1111/1758-2229.70155 (PMC12241448; doi:10.1111/1758-2229.70155)
Supplement: Supplementary file 13 — Table S4. Taxa summary for bacterial taxa differentially represented at the family or genus levels comparing samples grouped by the classification variables. [file EMI4-17-e70155-s011.pdf]

Table S4. Taxa summary for bacterial taxa differentially represented at the family or genus levels comparing samples grouped by the classification variables (filtered for sequence representation > 5 or 2% in at least one group, at the family or genus levels, respectively). Significant differences among the samples groups (Kruskal-Wallis t-test, $P < 0.05$), with the Bonferroni adjustment and the False discovery rate p-values, are shown in bold. Highest sequence representations in groups (%) are shown in red. Data analyzed with R library *mctoolsr.*

Taxa	p	pBon	pFDR	Crop	
				Banana	Control
f__uncl_Gammaproteobacteria	**0.000621759**	**0.001865277**	**0.001865277**	0.0509	0.0255
g__uncl_Gammaproteobacteria	**0.000621759**	**0.001865277**	**0.001865277**	0.0509	0.0255
f__Enterobacteriaceae	**0.037421866**	0.112265597	0.056132799	0.0521	0.0296
g__Enterobacteriaceae	**0.031137642**	0.093412927	**0.046706464**	0.0511	0.0282

Taxa	p	pBon	pFDR	Omnivorous/predatory nematodes*			
				H	L	M	None
f__Gemmataceae	**0.003271774**	**0.01963064**	**0.01963064**	0.0223	0.0653	0.00498	0.00893
f__Enterobacteriaceae	**0.008094431**	**0.04856659**	**0.02428329**	0.0185	0.0144	0.02669	0.06712
f__Thermogemmatisporaceae	**0.012761401**	0.07656841	**0.02552280**	0.0119	0.0573	0.01750	0.01639
f__uncl_Betaproteobacteria	**0.026835444**	0.16101266	**0.04025317**	0.0580	0.0434	0.02870	0.02607
f__Bacillaceae	**0.027075305**	0.16245183	**0.03249037**	0.0224	0.0109	0.06528	0.04268
g__uncl_TM7-1	**0.0006462674**	**0.01034028**	**0.01034028**	0.00	0.00	0.02800	0.00704
g__[Pedosphaeraceae]	**0.0036530241**	0.05844839	**0.02922419**	0.02245	0.03118	0.03352	0.00991
g__ uncl_Gemmataceae	**0.0060967353**	0.09754776	**0.03251592**	0.01761	0.06343	0.00498	0.00740
g__ uncl_Enterobacteriaceae	**0.0073901311**	0.11824210	**0.02956052**	0.01634	0.01369	0.02669	0.06643
g__ uncl_Thermogemmatisporaceae	**0.0127614014**	0.20418242	**0.04083648**	0.01197	0.05730	0.01750	0.01639
g__ uncl_Comamonadaceae	**0.0191886822**	0.30701891	0.05116982	0.03861	0.01172	0.02748	0.01795
g__uncl_Rhizobiales	**0.0213243125**	0.34118900	**0.04874129**	0.04070	0.04277	0.02975	0.02744
g__uncl_Betaproteobacteria	**0.0268354441**	0.42936710	0.05367089	0.05809	0.04348	0.02870	0.02607
g__uncl_Planctomycetia	**0.0357565806**	0.57210529	0.06356725	0.00751	0.02034	0.00	0.00483
g__ uncl_Acidobacteriaceae	**0.0440862547**	0.70538007	0.07053801	0.01026	0.02999	0.01662	0.00937

Taxa	p	pBon	pFDR	Other plant parasitic nematodes*		
				H	L	None
f__Rhodobacteraceae	**0.006573541**	**0.01314708**	**0.01314708**	0.00338	0.0511	0.0135
f__Enterobacteriaceae	**0.026740806**	**0.05348161**	**0.02674081**	0.01689	0.0845	0.0477
g__*Psychrilyobacter*	**0.000911882**	**0.01459011**	**0.01459011**	0.00	0.0230	0.00
g__Microbacteriaceae	**0.003907758**	0.06252412	**0.03126206**	0.00216	0.0252	0.0135
g__uncl_Gammaproteobacteria	**0.010289738**	0.16463581	**0.05487860**	0.02252	0.0453	0.0449
g__Rhodobacteraceae	**0.013668103**	0.21868964	**0.05467241**	0.00338	0.0418	0.0087
g__*Bacillus*	**0.015589452**	0.24943123	**0.04988625**	0.00365	0.0208	0.0139
g__Enterobacteriaceae	**0.017616477**	0.28186363	**0.04697727**	0.01512	0.0817	0.0469
g__ uncl_Bacillaceae	**0.034801628**	0.55682605	0.07954658	0.00878	0.0227	0.0206
g__uncl_Rhizobiales	**0.049847214**	0.79755542	0.09969443	0.04079	0.0155	0.0333

* Based on nematodes / 100 ml soil. L = low (≤ 90 % of all samples mean); M = medium (within mean ± 10%); H = high (≥ mean + 10%).
