# Supplementary material for: Farming System and Nematodes Affect the Rhizosphere Microbiome of Tropical Banana Plants
Source: Environ Microbiol Rep. 2025 Jul 9;17(4):e70155. doi: 10.1111/1758-2229.70155 (PMC12241448; doi:10.1111/1758-2229.70155)
Supplement: Supplementary file 14 — Table S5. Taxa summary for fungi differentially represented at the family or genus level, comparing samples grouped by the classification variables. [file EMI4-17-e70155-s003.pdf]

Supplementary Table S5. Taxa summary for fungal OTUs differentially represented at the family or genus level, comparing samples grouped by the classification variables (filtered for sequence representation < 5% in at least one group, at the family or genus levels, respectively). Significant differences among the samples groups (Kruskal-Wallis t-test, P < 0.05), with the Bonferroni adjustment and False discovery rate p-values, are shown in bold. Highest sequence representations in groups (%) are shown in red. Data analyzed with R library *mctoolsr.*

Taxa	p	pBon	pFDR	Crop (% of seq.)	
				Banana	Control
c_Sordariomycetes	**0.008828761**	0.05297257	0.05297257	0.30479549	0.14294980
o_Hypocreales	**0.006811918**	**0.04087151**	**0.04087151**	0.30374339	0.13285598
o_Capnodiales	**0.042389448**	0.25433669	0.12716834	0.10523454	0.07020117
o_Pleosporales	**0.044685326**	0.26811196	0.08937065	0.11015430	0.20561402
f_Hypocreaceae	**0.002250227**	**0.01575159**	**0.01575159**	0.29598122	0.10129580
f_Cladosporiaceae	**0.042389448**	0.29672614	0.14836307	0.10509401	0.07009411
f_ Pleosporaceae	**0.049534613**	0.34674229	0.11558076	0.04452063	0.08874210
g_*Trichoderma*	**0.002250227**	**0.01350136**	**0.01350136**	0.29597122	0.10129580
g_*Cladosporium*	**0.042389448**	0.25433669	0.12716834	0.10509401	0.07009411

Taxa	p	pBon	pFDR	Management (% of seq.)		
				Barbecho	Conventional	Organic
f_Didymosphaeriaceae	**0.001666786**	**0.01500107**	**0.01500107**	0.02947638	6.164427e-02	4.796752e-02
f_Onygenales_Inc_sedis	**0.017415216**	0.15673695	0.07836847	0.06515051	1.611322e-05	2.189765e-06
f_Clavariaceae	**0.021391251**	0.19252126	0.06417375	0.05179840	2.105485e-03	4.511628e-05
g_Didymosphaeriaceae_Inc_sedis	**0.00207265**	**0.01865385**	**0.01865385**	0.02947638	6.010935e-02	4.795803e-02
g_Onygenales_Inc_sedis	**0.01741522**	0.15673695	0.07836847	0.06515051	1.611322e-05	2.189765e-06
g_Clavariaceae_Inc_sedis	**0.02139125**	0.19252126	0.06417375	0.05012999	2.105485e-03	4.511628e-05

Taxa	p	pBon	pFDR	Management Description (% of seq.)					
				Barbecho Banana	Barbecho Control	Conventional Banana	Conventional Control	Organic Banana	Organic Control
c_Ascomycota_Inc_sedis	**0.01660870**	0.1162609	0.1162609	0.06060559	0.02191106	0.00000000	0.001075942	0.008149978	0.03605768
c_Sordariomycetes	**0.03425309**	0.2397716	0.1198858	0.37627255	0.05771614	0.21545895	0.246928816	0.336950383	0.12420444
o_Onygenales	**0.009145187**	0.1188874	0.11888743	7.483238e-06	0.11726493	2.495319e-05	7.273256e-06	0.00000000	04.238031e-04
o_Chaetothyriales	**0.015922268**	0.2069895	0.10349474	2.875105e-03	0.05448409	2.758436e-04	1.815231e-02	0.00000000	1.409744e-02
o_Ascomycota_Inc_sedis	**0.016608702**	0.2159131	0.07197104	6.060559e-02	0.02191105	0.000000e+00	1.075942e-03	0.00814997	3.605768e-02
o_Hypocreales	**0.029569700**	0.3844061	0.09610152	3.726101e-01	0.05216128	2.154589e-01	2.430448e-01	0.33693442	1.033619e-01
o_Agaricomycetes_Inc_sedis	**0.040549388**	0.5271420	0.10542841	0.000000e+00	0.00447823	0.000000e+00	2.679567e-02	0.01368389	5.017025e-02
f_Onygenales_Inc_sedis	**0.006580084**	0.08554109	0.08554109	7.483238e-06	0.117264934	2.495319e-05	7.273256e-06	0.000000000	4.379530e-06
f_Didymosphaeriaceae	**0.007998687**	0.10398293	0.05199146	3.056848e-02	0.028602709	6.408015e-02	5.920838e-02	0.066304413	2.963062e-02
f_Hypocreaceae	**0.015798891**	0.20538558	0.06846186	3.609564e-01	0.032658287	2.146839e-01	2.208800e-01	0.325298320	5.034910e-02
f_Ascomycota_Inc_sedis	**0.016608702**	0.21591312	0.05397828	6.060559e-02	0.021911059	0.000000e+00	1.075942e-03	0.008149978	3.605768e-02
f_Clavariaceae	**0.021189945**	0.27546929	0.05509386	8.912922e-05	0.093165809	4.189151e-03	2.181977e-05	0.000000000	9.023256e-05
g_Onygenales_Inc_sedis	**0.006580084**	0.09870126	0.09870126	7.483238e-06	0.117264934	2.495319e-05	7.273256e-06	0.000000e+00	4.379530e-06
g_Curvularia	**0.006861399**	0.10292098	0.05146049	0.000000e+00	0.034765439	3.537634e-05	1.536160e-02	8.939865e-05	7.162138e-02
g_Didymosphaeriaceae_Inc_sedis	**0.009983204**	0.14974806	0.04991602	3.056848e-02	0.028602709	6.408015e-02	5.613854e-02	6.630441e-02	2.961164e-02
g_Trichoderma	**0.015798891**	0.23698337	0.05924584	3.609215e-01	0.032658287	2.146839e-01	2.208800e-01	3.252983e-01	5.034910e-02
g_Ascomycota_Inc_sedis	**0.016608702**	0.24913052	0.04982610	6.060559e-02	0.021911059	0.000000e+00	1.075942e-03	8.149978e-03	3.605768e-02
g_Clavariaceae_Inc_sedis	**0.021189945**	0.31784918	0.05297486	8.912922e-05	0.090162684	4.189151e-03	2.181977e-05	0.000000e+00	9.023256e-05

Taxa	p	pBon	pFDR	*Meloidogyne* (% of seq.)*			
				H	L	M	None
p_Ascomycota	**0.03629373**	0.1088812	0.1088812	0.612335	0.54702742	0.51812202	0.69747
f_Schizoporaceae	**0.04024507**	0.4024507	0.4024507	2.771752e-03	0.16600560	0.000000e+00	0.000000
g_*Hyphodontia*	**0.00367113**	**0.0367113**	**0.0367113**	0.000000e+00	0.16600560	0.000000e+00	0.000000

Taxa	p	pBon	pFDR	Omnivorous/predatory nematodes (% of seq.)*			
				H	L	M	None
c_Eurotiomycetes	**0.04847378**	0.2908427	0.2908427	0.096778	0.247482	0.0912054	0.073601

Taxa	p	pBon	pFDR	Spiral nematodes *(% of seq.)**			
				H	L	M	None
f_Lycoperdaceae	**0.0157654**	0.189185	0.1891850	2.241366e-05	0.0595315	0.00071140	9.246484e-05
g_*Arachnion*	**0.0019768**	**0.023722**	**0.0237224**	2.241366e-05	0.0595315	0.00071140	0.0000000

Taxa	p	pBon	pFDR	*Radopholus* (% of seq.)*	
				High	None
c_Tremellomycetes	**0.0122948**	0.0860641	0.0860641	0.070762	0.012273
o_Tremellales	**0.0062215**	**0.0497723**	**0.0497723**	0.070762	0.007797
f_Phaeotremellaceae	**0.0250097**	0.2250878	0.2250878	0.059718	0.003022
g_*Hyphodontia*	**0.0032408**	**0.0291675**	**0.0291675**	0.110670	0.000000
g_*Phaeotremella*	**0.0250097**	0.2250878	0.1125439	0.059718	0.003022

Taxa	p	pBon	pFDR	Free living nematodes (% of seq.)*		
				H	L	None
o_Pleosporales	**0.0407034**	0.325627	0.325627	2.143732e-01	1.512672e-01	0.04182297

Taxa	p	pBon	pFDR	Other plant parasitic nematodes (% of seq.)*		
				H	L	None
c_Sordariomycetes	**0.00395733**	**0.02374402**	**0.02374402**	0.09210785	8.829652e-02	0.30032334
o_Onygenales	**0.00325254**	**0.02602032**	**0.02602032**	0.06534591	2.634304e-05	2.604898e-05
o_Hypocreales	**0.00612484**	**0.04899874**	**0.02449937**	0.08089880	8.829652e-02	2.966981e-01
o_Agaricales	**0.02809226**	0.22473811	0.07491270	0.07326573	4.130951e-05	1.201427e-02
f_Hypocreaceae	**0.00389267**	0.03503406	0.03503406	0.04592296	8.828155e-02	2.818502e-01
f_Onygenales_Inc_sedis	**0.00444395**	0.03999561	0.01999781	0.06515113	2.634304e-05	6.931441e-06
f_Clavariaceae	**0.00503431**	0.04530881	0.01510294	0.05181851	2.634304e-05	1.181797e-03
f_Cladosporiaceae	**0.04880605**	0.43925449	0.10981362	0.01125905	5.071485e-01	7.817219e-02
g_*Trichoderma*	**0.00389267**	**0.03503406**	**0.03503406**	0.04592296	8.828155e-02	2.818425e-01
g_Onygenales_Inc_sedis	**0.00444395**	**0.03999561**	**0.01999781**	0.06515113	2.634304e-05	6.931441e-06
g_Clavariaceae_Inc_sedis	**0.00503431**	**0.04530881**	**0.01510294**	0.05015010	2.634304e-05	1.181797e-03
g_*Cladosporium*	**0.04880605**	0.43925449	0.10981362	0.01125905	5.071485e-01	7.817219e-02

* Based on nematodes / 100 ml soil. L = low (≤ 90 % of all samples mean); M = medium (within mean ± 10%); H = high (≥ mean + 10%).
