# Supplementary material for: Farming System and Nematodes Affect the Rhizosphere Microbiome of Tropical Banana Plants
Source: Environ Microbiol Rep. 2025 Jul 9;17(4):e70155. doi: 10.1111/1758-2229.70155 (PMC12241448; doi:10.1111/1758-2229.70155)
Supplement: Supplementary file 16 — Table S7. PERMANOVA pairwise comparisons of ITS1 sequence profiles in samples grouped by different classification variables, based on Bray‐Curtis dissimilarity matrix. [file EMI4-17-e70155-s004.pdf]

Supplementary Table S7. Results of PERMANOVA pairwise comparisons of ITS1 sequence profiles in samples grouped by different classification variables (n. of samples = 29, based on Bray-Curtis dissimilarity matrix).

Var 1	Var 2	R^2	P [a]	P Bonferroni [a]	P FDR [a]
Crop					
Banana	Control	0.1029546	**0.005**	**0.005**	**0.005**
Management					
Barbecho	Conventional	0.12403210	**0.012**	**0.036**	**0.036**
Barbecho	Organic	0.07419984	0.140	0.420	0.140
Conventional	Organic	0.09788636	0.036	0.108	0.054
Description					
Barbecho Banana	Barbecho Control	0.22954157	0.068	1.020	0.113
Barbecho Banana	Conventional Banana	0.15517432	0.241	3.615	0.362
Barbecho Banana	Conventional Control	0.10778675	0.624	9.360	0.720
Barbecho Banana	Organic Banana	0.12409658	0.476	7.140	0.595
Barbecho Banana	Organic Control	0.22226488	0.061	0.915	0.114
Barbecho Control	Conventional Banana	0.30742867	**0.042**	0.630	0.090
Barbecho Control	Conventional Control	0.24377339	**0.029**	0.435	0.073
Barbecho Control	Organic Banana	0.31144830	0.021	0.315	0.063
Barbecho Control	Organic Control	0.21467323	**0.011**	0.165	0.165
Conventional Banana	Conventional Control	0.09530366	0.785	11.775	0.785
Conventional Banana	Organic Banana	0.12120540	0.376	5.640	0.513
Conventional Banana	Organic Control	0.32984256	**0.013**	0.195	0.097
Conventional Control	Organic Banana	0.09261329	0.747	11.205	0.800
Conventional Control	Organic Control	0.24461602	**0.017**	0.255	0.073
Organic Banana	Organic Control	0.31097960	**0.017**	0.255	0.073
Meloidogyne sp.[b]					
H	L	0.12606229	0.646	3.876	0.775
H	M	0.13255401	0.290	1.740	0.580
H	None	0.03789141	0.622	3.732	0.933
L	M	0.23075783	0.266	1.600	0.800
L	None	0.04044122	0.814	4.884	0.814
M	None	0.05990170	0.242	1.452	1.452

Omnivorous/predatory nematodes [c]					
H	L	0.09420354	0.181	1.086	0.362
H	M	0.06489461	0.938	5.628	0.938
H	None	0.10704905	**0.006**	**0.036**	**0.024**
L	M	0.27206901	0.400	2.400	0.600
L	None	0.18945594	**0.006**	**0.036**	**0.024**
M	None	0.05580642	0.718	4.308	0.862
Helicotylenchus spp. [d]					
H	L	0.12759948	0.377	2.262	0.754
H	M	0.09812240	0.406	2.436	0.609
H	None	0.03622360	0.481	2.886	0.577
L	M	0.42267048	1.000	6.000	1.000
L	None	0.07498466	0.305	1.830	1.830
M	None	0.05424907	0.373	2.238	1.119
Radopholus similis [e]					
H	None	0.03597866	0.422	0.422	0.422
Other plant parasitic nem. [f]					
H	L	0.16341863	0.095	0.285	0.143
H	None	0.12957344	**0.003**	**0.009**	**0.009**
L	None	0.04952939	0.449	1.347	0.449
Free-living nematodes [g]					
H	L	0.05048545	0.185	0.555	0.185
H	None	0.12702214	0.108	0.324	0.324
L	None	0.05874406	0.179	0.537	0.268

[a] Significant values ($P \leq 0.05$) are shown in bold.
[b] Nematodes / 100 ml soil. L = low density < 90% mean; M = medium density within mean ± 10%; H = high > 110% mean. All samples mean ± SD = 18 ± 46
[c] Nematodes / 100 ml soil. L = low density < 90% mean; M = medium density within mean ± 10%; H = high > 110% mean. All samples mean ± SD = 20 ± 27
[d] Nematodes / 100 ml soil. L = low density < 90% mean; M = medium density within mean ± 10%; H = high > 110% mean. All samples mean ± SD = 11 ± 17
[e] Nematodes / 100 ml soil. L = low density < 90% mean; M = medium density within mean ± 10%; H = high > 110% mean. All samples mean ± SD = 4 ± 16
[f] Nematodes / 100 ml soil. L = low density < 90% mean; M = medium density within mean ± 10%; H = high > 110% mean. All samples mean ± SD = 7 ± 17
[g] Nematodes / 100 ml soil. L = low density < 90% mean; M = medium density within mean ± 10%; H = high > 110% mean. All samples mean ± SD = 172 ± 212
